# Potent Preorganized Pyrazolidine Cyclophilin D Inhibitors Prevent Mitochondrial and Organ Injury in a Mouse Pancreatitis Disease Model

**DOI:** 10.1021/acs.jmedchem.5c01146

**Published:** 2025-11-10

**Authors:** Muhammad Awais, Christopher M. Woodley, Liqun Guo, Michael Rogers, Neil Kershaw, Thomas Zacharchenko, Emma Shore, Arjun Kattakayam, Rajarshi Mukherjee, David N. Criddle, Suet C. Leung, Heather Lee, Joshua Burgess-McCann, Konstantin Luzyanin, Neil G. Berry, Svetlana Antonyuk, Lu-Yun Lian, Robert Sutton, Paul M. O’Neill

**Affiliations:** † Department of Molecular and Clinical Cancer Medicine, Liverpool Pancreatitis Research Group, University of Liverpool, Liverpool L69 7BE, U.K.; ‡ Pharmacology and Therapeutics, Institute of Systems, Molecular and Integrative Biology, 4591 University of Liverpool, Liverpool L69 7GE, U.K.; § Robert Robinson Laboratories, University of Liverpool, Chemistry, Oxford Street, Liverpool L69 7ZD, U.K.; ∥ Liverpool University Hospitals NHS Foundation Trust, Liverpool L7 8XP, U.K.; ⊥ Wellcome Centre for Cell-Matrix Research, 5292 The University of Manchester, Michael Smith Building Dover Street, Manchester M13 9PT, U.K.

## Abstract

Cyclophilin D inhibitors that prevent opening of the mitochondrial permeability transition pore (MPTP) are potential treatments for a range of acute and chronic diseases, including acute pancreatitis. Here, we report that replacement of carbon with nitrogen in the pyrrolidine headgroup of a series of cyclophilin D inhibitors gives a dramatic enhancement in binding affinity (>40 fold), and prolyl isomerase inhibition (PPIase) activity (>200 fold), which is ascribed to a preorganization of the pyrazolidine amide headgroup. Protein–ligand X-ray crystal structures and NMR and molecular modeling demonstrate the importance of *cis*-amide geometry within the preorganized conformation, ensuring the ligand headgroup is anchored in the S1′ binding pocket, leading to potent nM PPIase inhibition and binding. Pyrazolidines potently inhibit MPTP opening and prevent pancreatic toxin-induced cell necrosis *in vitro*. *In vivo*, **18f** provided a significant improvement of acute pancreatitis biomarkers in the CER-AP mouse pancreatitis model, underlining the potential of this series.

## Graphical abstract

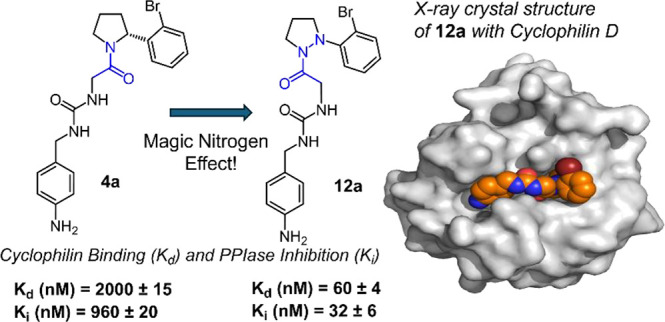

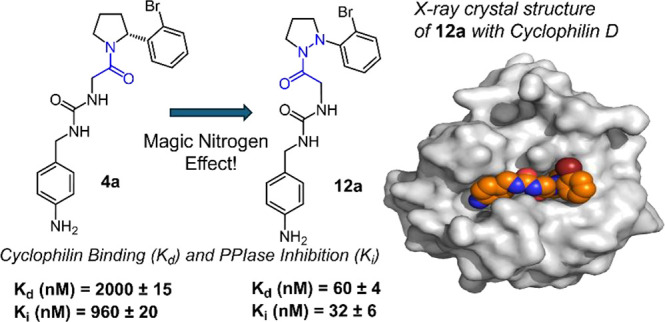

## Introduction

Mitochondrial injury and dysfunction are central to the pathogenesis of ischemia-reperfusion injury in a range of organs, notably the heart, brain, and kidney, alongside a range of diseases, including acute pancreatitis, muscular dystrophies, and neurodegeneration.^1–5^ Common to many, intracellular calcium or reactive oxygen species overload induces mitochondrial dysfunction through persistent opening of the mitochondrial permeability transition pore (MPTP). In this state, the MPTP allows uncontrolled proton flow across the inner mitochondrial membrane and an unregulated flux of water, ions, and solutes up to 1.5 kDa into and out of the mitochondrial matrix. The resultant loss of mitochondrial membrane potential (ΔΨ_m_) and consequently ATP production, coupled with disruption of calcium homeostasis, activates the necrotic cell death pathway.^2,6^

The mitochondrial matrix protein cyclophilin D (CypD) regulates opening of the MPTP, owing to its potent ability to sensitize the MPTP to calcium and oxidative stress.^1,2,4,5^ CypD belongs to the peptidyl-prolyl *cis–trans* isomerase (PPIase) cyclophilin (Cyp) family that catalyzes 180° rotation about the C–N linkage of peptide bonds preceding proline^7^ and of 17 isoforms of human Cyps, CypD is the only isoform located in the mitochondria. Cyps are so named because of their binding affinity for the lipophilic cyclic peptide cyclosporin A (CsA), which inhibits their PPIase activity and so inhibits CypD-mediated MPTP opening. Preclinical evidence demonstrates that genetic or pharmacological CypD inhibition reduces organ injury in a range of *in vivo* animal and human models of MPTP-related disease,^4^ confirming CypD as a target for drug discovery and development to treat these diseases.

Cyclosporin A (CsA) and its analogues have nanomolar binding affinity for Cyps, most notably CypA, CypB, and CypD.^8,9^ CsA is an immunosuppressant widely used in autoimmune disease and against rejection in solid organ transplantation. The interaction of CsA with cytosolic CypA generates a complex that has an ability to bind to and inhibit phosphatase calcineurin.^10,11^ As a consequence, the calcineurin substrate, phospho-nuclear factor of activated T-cells (pNFAT), is unable to translocate to the nucleus and initiate an immune response.^9,11^ Nonimmunosuppressant semisynthetic analogues of CsA, such as Debio 025 and NIM811, maintain inhibition of Cyps, but their cyclophilin complexes do not bind to calcineurin.^9^ CsA and its analogues have unfavorable drug-like characteristics, however, with high molecular weights, limited solubility, poor bioavailability, and well-documented adverse effects.^12–14^ In recent years, potent small-molecule MPTP inhibitors have been reported, but their exact target(s) are unknown.^15,16^ Therefore, small-molecule CypD inhibitors that have high affinity and solubility, exhibit no immunosuppressive activity, and possess an improved pharmacokinetic/pharmacodynamic profile to treat MPTP-related disease.

In previous work, we and others focused on the optimization of reported aniline-containing urea-based CypD inhibitors exemplified in compounds **1** and **2** (Figure 1).^17–19^ Functionalization of the α carbon (C_α_) of the pyrrolidine (Pyr) in **2** with substituted aryl rings resulted in a significant increase in binding affinity across the series. Through this work we determined that the C_α_ R-enantiomer was optimal for interaction with CypD (exemplified in **3a** and **4a**). The most potent compound of this series (compound **3a**) possessed a *K*_d_ of 0.41 μM, as determined by isothermal titration calorimetry (ITC) binding.^19^ Comparison of X-ray crystallographic structures of **3a** and CsA in complex with CypD (PDB: 4J5C and PDB: 2Z6W, respectively) showed that the (*S*)-methionine in **3a** and the butenyl-methyl-l-threonine (Bmt) residue of CsA occupy similar space.^19^ The (*S*)-methionine backbone is observed to form hydrophobic interactions with the binding pocket (PDB: 4J5C); however, this was shown not to be essential to binding, and replacement with glycine (illustrated by compound **4a**) maintained satisfactory binding.^19^

**Figure 1:**
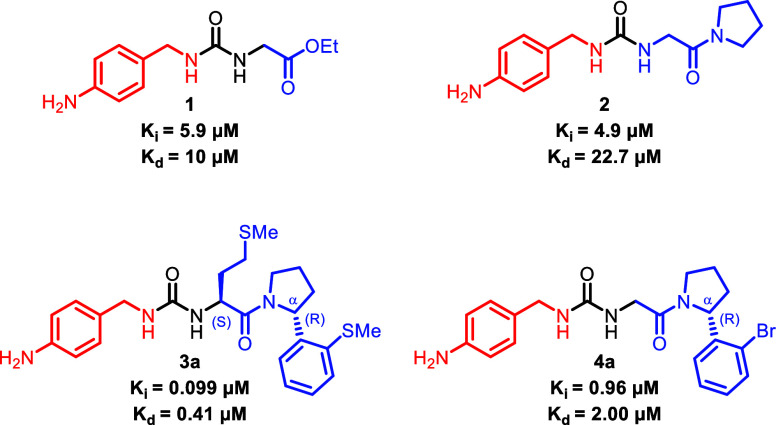
Cyclophilin D inhibitors with ITC-derived binding constants (*K*_d_) and PPIase-derived inhibition constant *K*_i_. The right-hand side (RHS) and left-hand side (LHS) of the molecule are highlighted in blue and red, respectively.

The X-ray crystal structures of **3a** and **4a** (PDB: 4J5D) in complex with CypD reveal that this series of compounds preferentially adopt a puckered *cis*-amide Pyr geometry in their bound state (Figure S1). We reasoned that replacement of the chiral Pyr moiety with a pyrazolidine (Pz) would provide a conformational *cis* bias, already established in the literature for related Aza-proline (AzaPro) peptide residues.^20^ In contrast to Pro residues, which possess a modest preference for the trans geometric isomer (Figure 2A, calculated Δ*G*° = −0.5 kcal/mol), AzaPro residues in peptide chains favor the *cis* geometric isomer (Figure 2B, calculated Δ*G*° = 2.1 kcal/mol). This is attributed to the unfavorable electrostatic repulsion of the residual lone pair of N_α_ with that of the preceding carbonyl oxygen. Figure 2C shows the calculated isosurfaces for carbonyl and N_α_ lone pair natural bonding orbitals (NBOs); in the trans geometric isomer, there exists a clear overlap between the two lone pairs, illustrating this unfavorable interaction. Peptides containing the AzaPro residues have been shown to populate the cis conformation by 75%.^20^ By inclusion of the Pz proline mimetic, we aimed to investigate the effects of this conformational preorganization on the activity of this class of Cyp inhibitors.

**Figure 2:**
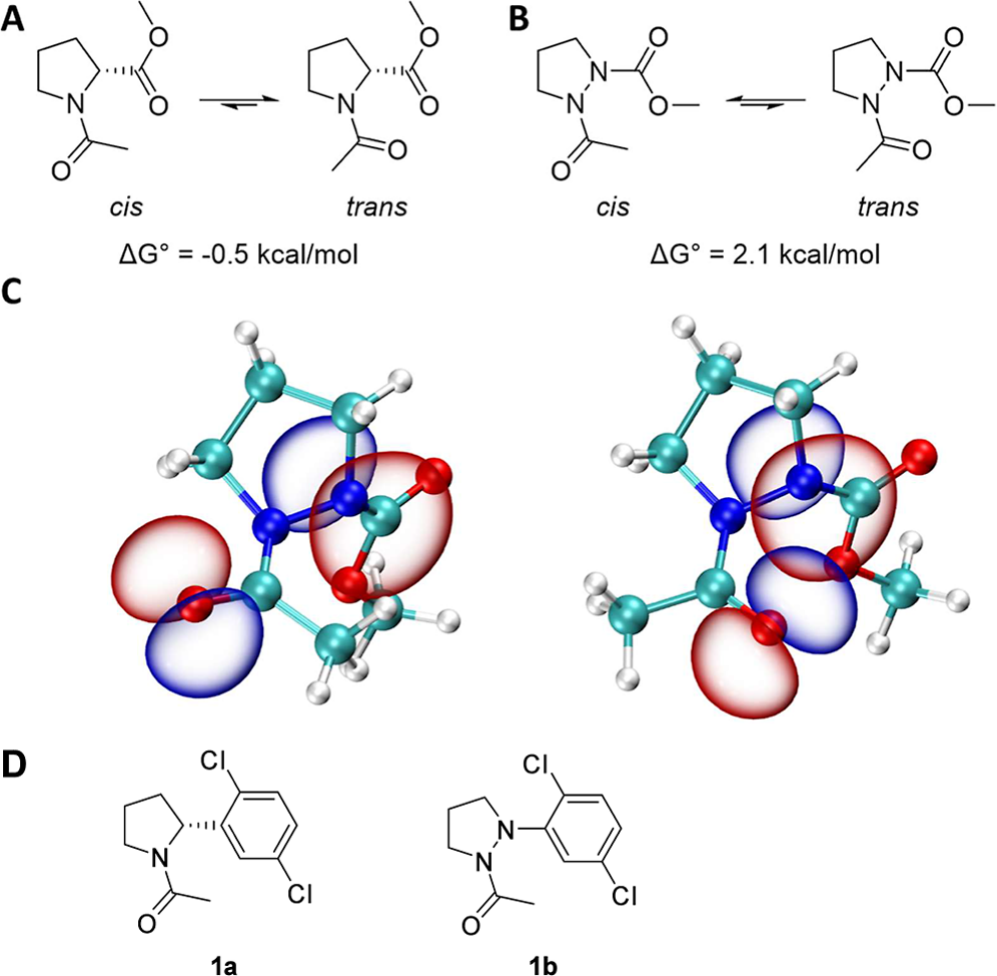
(A) Equilibrium between cis- and trans-geometric isomers of methyl acetyl-d-prolinate. Density functional theory (DFT) calculated Δ*G*° = −0.5 kcal/mol. (B) Equilibrium between *cis*- and *trans*-geometric isomers of methyl 2-acetylpyrazolidine-1-carboxylate. Calculated Δ*G*° = 2.1 kcal/mol (computational details are included in Supporting Information). (C) DFT-optimized geometries of methyl 2-acetylpyrazolidine-1-carboxylate in the lowest energy *cis* (left) and *trans* (right) geometric isomers. Isosurfaces of lone pair NBOs generated using Multiwfn^21^ and rendered in VMD.^22^ (D) Matched pair amides **1a** and **1b** used in NMR studies.

Here, we describe the synthesis of two novel series of CypD small molecule inhibitors (Series 1 and 2) containing the key Pz headgroup. Representatives from both series were characterized and compared in detail by X-ray crystallography, *in vitro* binding and inhibition assays, and *in silico* calculations, together with drug metabolism and pharmacokinetic (DMPK) testing. The ability of these compounds to inhibit MPTP opening and cell necrosis was determined using live isolated mitochondria and pancreatic acinar cells challenged with calcium and pancreatitis toxin, respectively. Pharmacokinetic (PK) assessment was undertaken on two selected molecules, one of which was tested for its therapeutic efficacy in experimental acute pancreatitis. Our results indicate that Pz urea CypD inhibitors have significant potential for further development against acute pancreatitis and other MPTP-related diseases.

## Results

Prior to target synthesis, we prepared a simplified matched pair of Pyr versus Pz headgroup probes **1a** and **1b** (Figure 2D), and we examined their solution conformational preferences using ^1^H NMR studies. We observed only one *cis* geometrical isomer at 298 K for **1b** by ^1^H NMR compared with **1a** (1.1/1 *trans*/*cis* mixture), and this provided the structural basis for our synthesis approach (See Figures S7–S12, Tables S1 and S2, Supporting Information).

Pz **12a**–**12d** (Series 1) were designed as matched-molecular pair equivalents to our previous Pyr compounds **4a**–**4d**.^19^ The RHS Pz fragment of this series was synthesized via one of two steps; cyclization or Buchwald–Hartwig coupling (Scheme 1A). The cyclization route began with the boc-protection of the relevant phenylhydrazine with treatment with boc-anhydride. This was followed by cyclization with 1,3-diiodopropane to afford the intermediate boc-protected Pz **7a**–**7c** in 43–66% over two steps. The 2-chloro-4-methoxyphenyl RHS was synthesized by methylation of 3-bromo-4-chlorophenol followed by Buchwald–Hartwig coupling of *N*-boc-pyrazolidine with 2-bromo-1-chloro-4-methoxybenzene using Pd_2_(dba)_3_ and *t*BuXPhos as a catalyst system, affording **7d** in 15% yield over two steps.

**Scheme 1:**
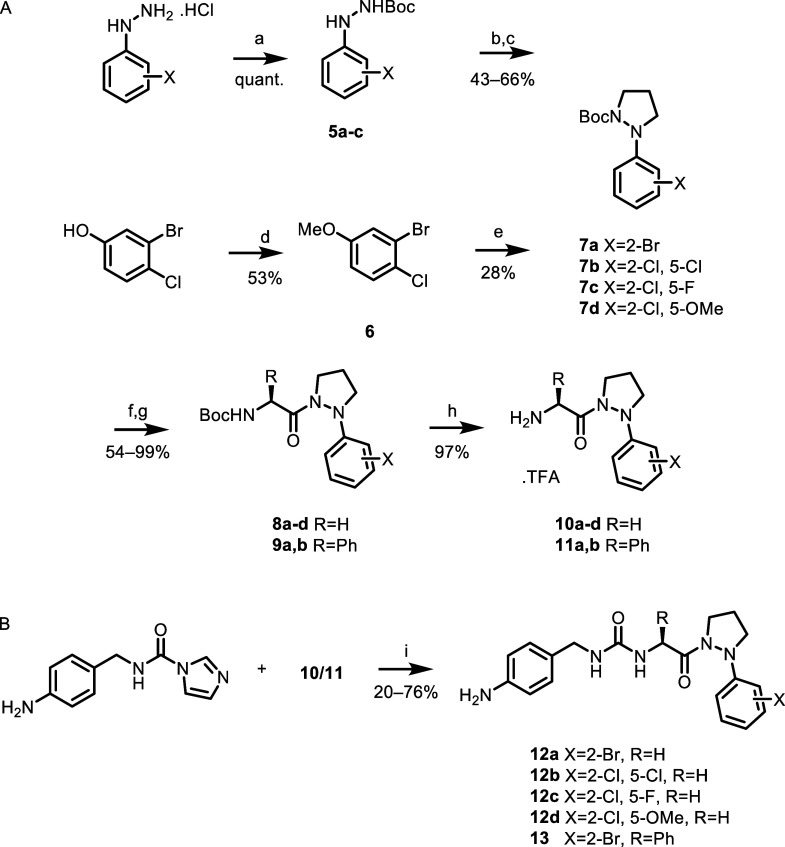
Synthesis of Series 1 Pz-based CypD Inhibitors^a^

a Reagents and conditions: (a) Boc-anhydride, NEt_3_, THF, rt, o/n. (b) *n*-BuLi, THF, −78 °C to rt, 15 min (c) 1,3-diiodopropane, THF, −78 °C, 1 h to rt, o/n. (d) Iodomethane, K_2_CO_3_, acetone, rt, o/n. (e) *tert*-Butyl pyrazolidine-1-carboxylate, Pd_2_(dba)_3_, *t*BuXPhos, Cs_2_CO_3_, toluene, reflux, o/n. (f) DCM/TFA (5:1), rt, 2 h. (g) Boc-glycine, HATU, DIPEA, DMF, rt, o/n. (h) CDI, DMAP, DCM, 0 °C to rt, o/n. (i) NEt_3_, DMAP, MeCN, rt, o/n.

To afford amines for coupling to the appropriate left-hand side (LHS), boc-protected Pz compounds were boc-deprotected and then coupled with either boc-glycine, affording compounds **8a**–**d** or boc-(*S*)-phenylglycine, producing compounds **9a** and **9b**. Boc-deprotection released amines **10a–10d** and amines **11a** and **11b** as trifluoroacetate salts (Scheme 1A). Aniline LHS-containing analogues were synthesized by coupling of the relevant amine **10a**–**10d** or **11a** with the carbonyldiimidazole adduct of 4-(aminomethyl) aniline in 20–76% yield (Scheme 1B).

Grädler and co-workers demonstrated that incorporation of a glycan-fused aniline in the LHS of a Pyr-based scaffold gave a substantial increase in drug binding affinity.^23^ Inspired by this work, Series 2 molecules **18a**–**18e** were designed to include a fused glycan in the LHS (Scheme 2). **15** was synthesized by selective boc-protection of 4-(aminomethyl)­aniline to afford **14**. This was used in a montmorillonite-catalyzed condensation with 2-deoxy-d-ribose to afford the boc-protected glycan-amine (**15**), with the hydroxyl subsequently protected by treatment with TBDMS triflate to give **16** in 60% yield. Boc-deprotection followed by treatment with CDI resulted in adduct **17**. Coupling of **17** to the appropriate RHS followed by TBDMS deprotection by treatment with TBAF in THF afforded the glycan Pz analogues **18a**–**18e**.^23^

**Scheme 2:**
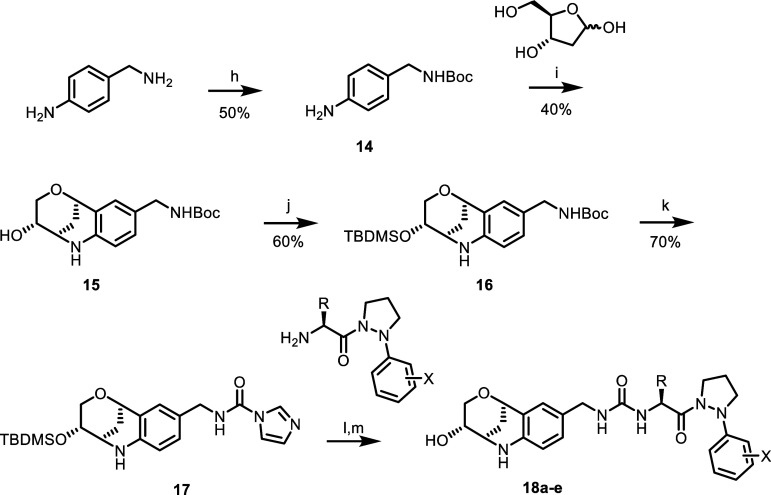
Series 2 Pz-based Cyc D Inhibitors^a^

a Reagents and conditions: (h) Boc anhydride, NEt_3_, THF, rt, 6-24 h. (i) montmorillonite, MeCN, rt, 3-5 Days. (j) TBDMS Triflate, 2,6-Lutidine, DCM, rt, 18 h. (k) (i) TFA, DCM, rt, 18 h. (ii) CDI, DMAP, DCM, rt, 24 h. (l) NEt_3_, DMAP, MeCN, rt, 16 h. (m) TBAF, THF, rt, 16 h.

For comparison, the Pyr analogue of **18b** (compound **19**) was synthesized in the same manner as **18a**–**18e** through coupling of the glycan LHS with the appropriate Pyr-containing RHS; the synthesis of chiral Pyr RHS has been previously described (Scheme S1).^19^

We were pleased to find that replacement of the Pyr ring in compound **4a** (*K*_i_ = 960 nM, *K*_d_ = 2000 nM) with the corresponding Pz ring to give **12a** resulted in a remarkable 30-fold increase in potency (**12a**, *K*_i_ = 32 nM) and 33-fold increase in affinity (*K*_d_ = 60 ± 4 nM) (Table 1). Similar effects were seen across the whole matched pair series (**4b**–**4d** Pyr/**12b**–**12d** Pz), with the Pz-modified analogues displaying much superior nanomolar PPIase inhibition (up to >200 fold **4d** cf. **12d**) and binding (up to >45 fold **4a** cf. **12a**) when compared with their Pyr counterparts. The dramatic effects seen through this “magic nitrogen effect” are ascribed to the preorganization of the prolyl mimetic Pz headgroup, which is anchored efficiently in the S1′ pocket. The rationale for the selection of the *ortho* chloro (R^1^) group in analogues **12b**–**12d** was to provide a more metabolically stable alternative for the *ortho* bromo in compound **12a**; also, it was proposed that an *o*-Cl substituent would provide a similar bond angle to the *o*-Br analogue, providing the correct geometry for binding (see for example Figure 3). The rationale for the addition of a second halogen in **12b** and **12c** at the R^2^ position was 2-fold: (i) first, we reasoned that binding may be improved through hydrophobic interactions with Leu 122 given the close proximity in the X-ray crystal structure; (ii) second, we proposed that dihalogenation would block potential P450 aromatic oxidation. Inspection of the crystal structure along with docking reveals that overall the 2,5-substitution pattern provides both hydrophobic (Leu122 and Phe59) and edge-to-face π–π stacking interactions (Phe59). With respect to metabolism, this approach failed to enhance metabolic stability for the aniline series (but had some positive impact on the glycan-fused series (Compare **18a** with **18c**/**18d**)) (vide infra, Table 2).

**Table 1:**
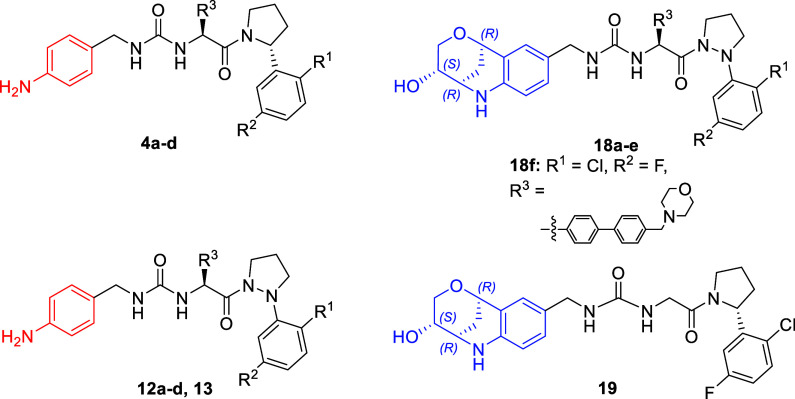
*K*_i_ and *K*_d_ Derived from PPIase and ITC Assays, Respectively, of CypD Inhibitors for Comparing Parent Pyr Compounds (**4a**–**d**) with Pz Anilines (**12a–d**, **13**) and Glycan-Fused Analogues (**18a–d**, **19**)

compound	R^1^	R^2^	R^3^	*K*_i_ (nM)^a^	*K*_d_ (nM)^b^
**4a**	Br	H	H	960 ± 20	2000 ± 15
**4b**	Cl	Cl	H	5600	ND
**4c**	Cl	F	H	3300	1300
**4d**	Cl	OMe	H	17300	ND
**12a**	Br	H	H	32 ± 6	60 ± 4
**12b**	Cl	Cl	H	460	ND^b^
**12c**	Cl	F	H	68	200 ± 30
**12d**	Cl	OMe	H	73	1100 ± 350
**13**	Br	H	Ph	5.0 ± 0.5*	19
**18a**	Br	H	H	2.5 ± 0.2*	40 ± 10
**18b**	Cl	F	H	2.4 ± 0.1*	10 ± 2
**18c**	Cl	OMe	H	1.9 ± 0.1*	40 ± 5
**18d**	Br	H	Ph	2.4 ± 0.2*	80 ± 40
**18e**	Cl	OMe	Ph	2.7 ± 0.2*	ND^b^
**18f**	Cl	F		2.4 ± 0.1*	ND^b^
**19**				6.9 ± 0.5*	20 ± 2
**CsA** ^18^				8.2 ± 0.4	10 ± 1

a *K*_i_ values measured by the functional PPIase assay * performed at Eurofins according to the method of Janowski.^38^ Values without standard error were measured in singlicate as part of the early-stage optimization; compounds with significantly improved profiles were measured in duplicate.

b *K*_d_ value measured by ITC (Figure S4). Compounds **12b**, **18e**, and **18f** were prone to precipitation during the ITC titration, rendering the data unreliable. (ND-not determined). CsA = cyclosporin A.

**Figure 3:**
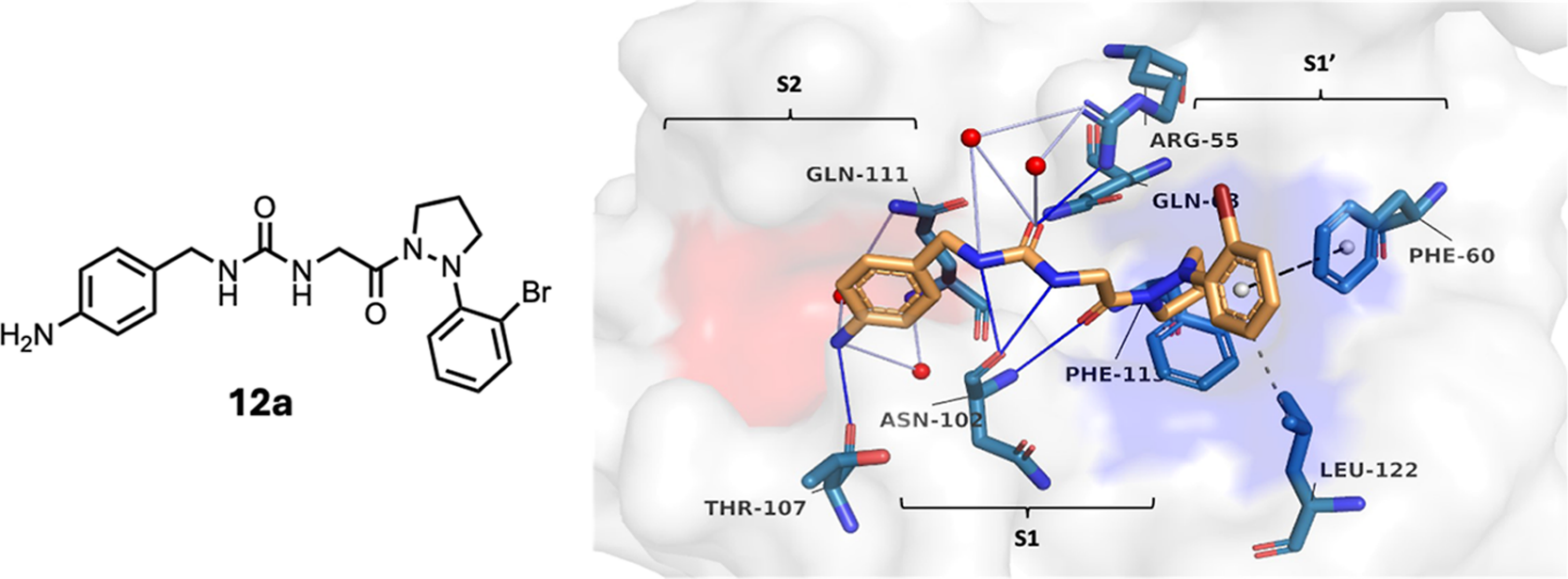
**C**ompound **12a** binding to CypD with key interactions highlighted. The compound and key protein residues are shown as sticks. Color scheme: protein—light gray surface, S1′ pocket—blue surface, S2 pocket—red surface. Amino acids: cyan—carbon, blue—nitrogen, red—oxygen. Ligand: orange—carbon, blue—nitrogen, red—oxygen, and dark red—bromine. Interactions between ligand and protein: gray dashed lines—hydrophobic interactions; blue lines—hydrogen bonding; light-blue lines—water bridges; black dashed lines—T-shaped π–π interactions. X-ray crystal structure (PDB 9H0S) was rendered in the PyMOL Molecular Graphics System, Version 1.7.4.4, Schrödinger, LLC. PLIP was used for the interaction analyses.^24^

**Table 2:** Summary of DMPK Data Comparing Pyr and Pz CypD Inhibitors^a^

compound	log *D* (pH 7.4)	solubility (μM)	H. protein binding^a^ (%)	rat heps Clint^b^ (μL/min/10^6^ cells)	H. mics Clint^c^ (μL/min/mg)
**4a**	2.1	365	98	78.5	250.1
**4c**	2.3	378	97	43.1	>300
**4d**	1.9	222	94	39.1	174.2
**12a**	1.7	431	77	45.4	106.2
**12b**	2.0	507	86	79.3	185.3
**12c**	1.6	765	77	55.3	120.3
**12d**	1.6	115	84	45.9	62.8
**13**	3.2	11	82	45.4	>300
**18a**	1.4	786	83	15.5	64.5
**18b**	1.2	941	80	12.6	57.3
**18c**	1.4	>1000	87	20.4	47.3
**18f**	3.9	97	97	18.5	43.6
**19**	1.5	901	99.3	25.3	143.5

a Human plasma protein binding.

b Rat hepatocyte intrinsic clearance.

c Human microsomal intrinsic clearance.

The crystal structure of **12a** in complex with CypD was determined (PDB accession number 9H0S (Figure 3)), and ligand–protein interactions were compared with the **4a-CypD** (PDB 4J5D) complex using PLIP.^24^ A summary of noncovalent interaction distances is given in Table S5. Superimposing the crystal structures of **4a** and **12a** over each other shows that the LHS and urea core align extremely well (Figure S2). The main interactions (Figures S1 and 3) between the *ortho* bromo-substituted aryl ring and the amino acid residues Arg55, Phe113, and Leu122 of the S1 pocket are found in both structures. In the **12a** complex, an additional hydrophobic interaction is observed between Phe60 and the aryl ring; the Pz ring pucker appears to position the aryl ring nearer to Phe60 than the chiral Pro ring in **4a**. Another subtle difference is observed in the S2 pocket; the hydrogen bond between the Arg82 side-chain and the aniline NH group in the **4a** complex is replaced by a Thr107-aniline NH_2_ hydrogen bond in the **12a** complex; the Arg82 side chain is oriented away from the ligand, widening the S2 pocket. Otherwise, all other interactions between the ligand and the binding site in the chiral Pro analogue **4a** complex are also observed in the **12a** complex.

The incorporation of (*S*)-phenylglycine into the backbone of the molecule was hypothesized to further increase the *cis*-amide preference of these Pz compounds through intramolecular π–π stacking interactions. The phenylglycine analogue of **12a** was synthesized by the use of *(S)*-boc-phenylglycine in place of boc-glycine in the general synthetic route (Scheme 1) to afford compound **13** (Table 1). Screening of compound **13** in the PPIase assay showed a *K*_i_ of 5.0 nM (Figure S6), representing a 6-fold increase in potency; the ITC data show a 2-fold increase in affinity for **13** (Table 1).

As noted earlier, for Series 2 analogues, we incorporated a glycan-fused LHS reported by Grädler *et al*.; the incorporation of the glycan-fused LHS in the Pyr scaffold has been reported to result in a substantial increase in drug binding affinity.^23^ The activity data for head-to-head comparison of glycan-Pz and aniline-Pz inhibitors are summarized in Table 1. We considered analogues with either glycine or phenylglycine modified backbones to determine if there was an additive effect on potency. Addition of the glycan moiety to glycine-containing Pz compounds **12a**, **12c,** and **12d** to give **18a**–**18c** afforded a significant increase (>15-fold) in potency as measured by both *K*_i_ and *K*_d_ values. However, incorporation of glycan into the phenylglycine analogue **13** to give **18d** (Figure 4) gave a 2-fold decrease in *K*_d_. Likewise, the incorporation of phenylglycine into the glycan **18c** to give **18e** afforded a nearly 2-fold loss in potency as measured by *K*_i_.

**Figure 4:**
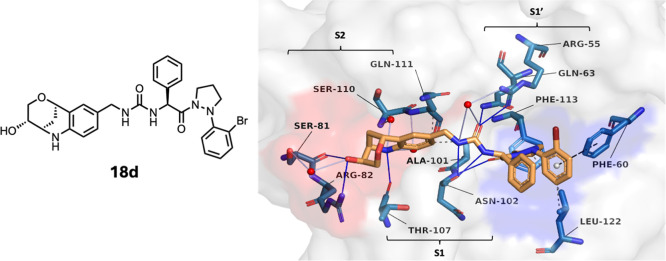
Compound **18d** complexed to CypD with key interactions highlighted. The compound and protein residues are shown as sticks. Color key: protein—light gray surface, S1′ pocket—blue surface, S2 pocket—red surface. Amino acids: cyan—carbon, blue—nitrogen, red—oxygen. Ligand: orange—carbon, blue—nitrogen, red—oxygen, and brown—bromine. Interactions between ligand and protein: gray dashed lines—hydrophobic interactions; blue lines—hydrogen bonding; light-blue lines—water bridges; and black dashed lines—T-shaped π–π interactions. X-ray crystal structure (PDB 9H0S) was rendered in PyMOL Molecular Graphics System, Version 1.7.4.4, Schrödinger, LLC. PLIP was used for the interaction analyses.^24^

Lastly, we showed the superiority of the Pz-based glycan-fused analogue compared with the Pyr equivalent. To do this, Pyr glycan **19** was prepared as a matched compound for **18b**. (Scheme S1) The chiral Pyr analogue **19** was ca. 3-fold less potent in PPIase assays with a weaker performance in the mitochondrial calcium retention capacity (CRC) assay compared to its counterpart **18b**.

To explore further expansion of the phenylglycine side chain, we synthesized the biaryl-methylmorpholine analogue **18f**. We found that extension of the phenylglycine side chain did not affect binding compared to **18d**. The extension of the phenyl glycine unit in **18f** was performed to provide a compound with additional lipophilicity and a basic nitrogen center; we proposed that this approach would (i) provide a site for potential salt formulation and (ii) provide a compound with a longer half-life *in vivo* through increasing the volume of distribution.

To understand the structural effects of a glycan moiety on interactions in the LHS pocket, the X-ray crystal structure of **18d** in complex with CypD was determined (Figure 4, PDB entry 9HTR). A summary of interaction distances is given in Table S6. This structure shows extra hydrogen bonds between the glycan alcohol and the Ser81 side-chain hydroxyl group, between the glycan ring oxygen and the Arg82 guanidino side chain, and a hydrogen bonding network via a bridging water molecule between the glycan nitrogen and Gly74, Ser110, and Gln-111. These extra hydrogen bonds explain the highly favorable enthalpic changes observed between the binding of **18c** (Δ*H* = −15.2 kcal/mol; −*T*Δ*S* = 4.65 kcal/mol) compared with that observed for the nonglycan phenylglycine analogue **13** (Δ*H* = −8.54 kcal/mol; −*T*Δ*S* = −1.95 kcal/mol). The RHS interactions between the ligand and protein appear to be preserved from the aniline series.

Favorable enthalpies (Δ*H*) dominate the Gibbs free energy (Δ*G*) of binding of these ligands to CypD (Figures S4 and S5). For **4a** and **13**, entropic changes (−*T*Δ*S* of, respectively, −3.85 and −1.95 kcal/mol) make favorable contributions to the overall Δ*G* of interactions. Comparing the data for **4a** (Δ*H* = −8.09 kcal/mol; −*T*Δ*S* = −3.49 kcal/mol) and Pz compound **12a** (Δ*H* = −17.2 kcal/mol; −*T*Δ*S* = 7.4 kcal/mol), the starting point of this ligand campaign, the switch from Pyr to Pz made Δ*H* more and −*T*Δ*S* less favorable with an overall improved binding for **12a** driven by the favorable enthalpic changes. As stated above, the crystal structures of these two complexes do not show new direct hydrogen bonds formed between **12a** and CypD compared with binding **4a** to explain the increasingly negative Δ*H*; it is likely that the change in enthalpy arises from other factors, possibly involving water molecules and internal conformational changes within the protein. The addition of the phenylglycine to compound **12a** to yield compound **13** (Δ*H* = −8.54 kcal/mol; −*T*ΔS = −1.95 kcal/mol) leads to an enthalpy–entropy compensation but with a more favorable entropy change, resulting in a 2-fold increase in affinity for **13** (|ΔΔ*H*| = +8.66 kcal/mol; |Δ*T*Δ*S*| = −9.35 kcal/mol).

Notably, the ITC data here (Figure S5) show that there is less variation in Δ*H* in the binding of the Pz compounds compared to the binding of Pyr compounds. The more rigid Pz compounds appear to be favorably anchored in the RHS pocket. The addition of a glycan moiety to compounds **12a**, **12c**, and **12d** to give **18a**–**c** resulted in improved average affinities: **12a** vs **18a** (*K*_d_ = 60 nM vs 40 nM); **12c** vs **18b** (*K*_d_ = 200 nM vs 10 nM); **12d** vs **18c** (*K*_i_ = 73 nM vs 1.9 nM; *K*_d_ = 1.1 μM vs 40 nM). Overall, the glycan-based compounds bind with consistently high average Δ*H* values, ranging from −10.1 to −16.1 kcal/mol. The extra hydrogen bonds between the glycan oxygens and the CypD side chains described above most likely explain the very notable increasingly negative Δ*H* of binding glycan-containing compounds, which leads to higher affinities for this subseries.

The situation is different when comparing the molecules with phenylglycine; the aniline compound **13** binds more strongly, with a higher inhibitory effect, than the glycan equivalent, **18d**. In this pair, although the enthalpic gain conforms to the other glycan molecules, this is negated by a decrease in Δ*S* for **18d** (unlike an increase in Δ*S* on binding **13**), leading to an overall decrease in Δ*G* and lower affinity.

Pyr and Pz analogues were submitted for drug metabolism and pharmacokinetic (DMPK) testing to determine the effects of the additional nitrogen on drug-like properties (Table 2). DMPK property data were measured once through a high-throughput platform provided by AstraZeneca UK. The methods of the five assays, including log *D* 7.4, aqueous solubility, plasma protein binding, and microsome and hepatocyte clearance measurements, have been reported previously.^25^

The compounds selected for *in vitro* DMPK measurements are shown in Table 2. Matched pairs of Pyr and Pz anilines **4a**, **c**, **d** and **12a**, **c**, **d**, respectively, showed that the addition of a nitrogen had a very slight effect on lipophilicity with a 0.1–0.3 difference in log *D* from Pro to Pz. Despite both pairs showing a reduction in log *D*, the effect on solubility was varied. From **4c** to **12c** solubility was improved, whereas between **4d** to **12d** solubility was found to decrease. It should, however, be noted that both pairs of compounds display acceptable aqueous solubility and are unlikely to cause issues in preclinical development. Metabolic stability was also observed to differ among the pairs of compounds. For all anilines, rat hepatocyte clearance was found to be moderate, with reduced stability observed for the Pz compounds. In contrast, improvements in human microsomal clearance were observed in the Pz series (for predicted sites of metabolism, see Table S3); between compounds **4d** and **12d**, an almost 3-fold improvement was seen. In terms of human plasma protein binding, matched pair analyses show a significant reduction in binding for the Pz series (**12a**, **12c**, and **12d**) compared with the corresponding Pro series (**4a**, **4c**, and **4d**).

The glycan compounds **18a**–**18c** were also screened for their DMPK properties. Comparing **18a**, **b**, **c** and their aniline counterparts **12a**, **c**, **d**, a reduction in log *D* was observed, which was also reflected in improved aqueous solubility; compound **18c** displayed the greatest solubility of all analogues tested (>1000 μM). As noted above, incorporation of the glycan moiety afforded substantial improvements in metabolic stability in both rat hepatocytes and human liver microsomes.

Comparing **18b** and its Pyr counterpart **19**, the incorporation of an extra nitrogen had the unexpected effect of increasing log *D*; however, this was not reflected in increased solubility, and both compounds possess comparable aqueous solubility values. In terms of metabolism, **18b** vs **19**, we observed a 2-fold and 3-fold reduction in rat hepatocyte and human microsomal clearance, respectively. This contrasts with similar Pz-Pyr matched pairs for the aniline series, where an increase in rat hepatocyte clearance was observed. Lastly, comparing the extended phenylglycine analogue **18f** with its glycine counterpart **18b**, the most notable effects are on log *D* and solubility, where we see an increase of 2.7 log units in log *D* and a corresponding 10-fold decrease in solubility. Despite an increased lipophilicity, the metabolic profile of **18f** is largely similar to that of **18b**, where the rat hepatocyte clearance is slightly increased but remains low and the human microsomal clearance is slightly decreased but remains moderate. For **18a**–**c** protein binding is around 80% for all analogues; as expected, increasing lipophilicity, as in **18f**, raises protein binding to 97%; the advantage of the Pz unit is again apparent from matched pair analysis of **18b** with **19**, which shows almost a 20% difference, with **18b** having a much higher unbound free fraction. Overall, the DMPK profiles of these compounds show that the incorporation of glycan-fused LHS alongside the Pz moiety has afforded improvements to the metabolic stability of our previously reported Pyr inhibitors.^19^

The inhibitory effect of Pz compounds on MPTP opening was assessed in isolated mitochondria loaded with the fluorescent calcium-sensitive dye Calcium Green 5N^26^ to measure CRC, which is lost with MPTP opening. A progressive increase in CRC was seen with the Pz compounds (Figure 5A), indicating inhibition of the MPTP broadly consonant with improvements in CypD binding and dissociation constants. The phenyl group of **13** that contributed to an increase in CypD affinity, however, did not have as marked an effect on CRC, whereas the glycan moiety improved performance of both Pz compound **18c** and, to a lesser extent, Pyr compound **19**. Marked superiority of the Pz compared to the Pyr series was shown by the ratio of the number of calcium pulses required to trigger the MPTP in the presence or absence of an inhibitor (CRC/CRC_0_) (Figure 5B). Concentration-dependent effect of inhibitors on CRC was demonstrated using **8** different concentrations of **18f** (Figure 5C). At 10 μM, **18a**–**f** increased mitochondrial CRC ×4.7, 4.5, 4.7, 4.4, 4.4, and ×5, respectively, closely approaching CsA at ×5.2 (Figure 5B); at lower concentrations, **18b**–**f**, particularly **18d** and **18f**, were more effective than CsA (Figure 5D).

**Figure 5:**
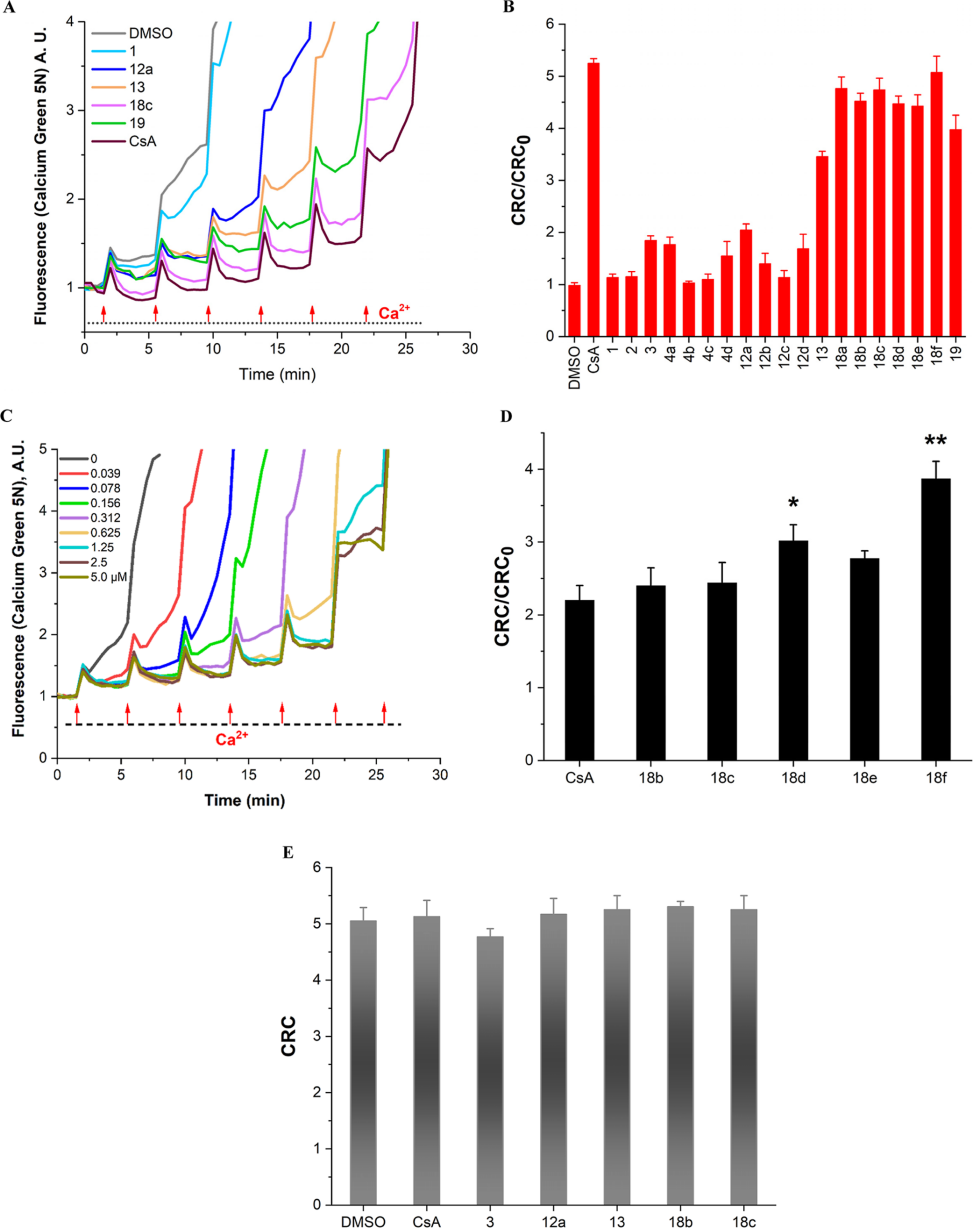
Assessment of the protection of isolated mitochondria by small molecule CypD inhibitors with the CRC assay: (A) representative changes in CRC following mitochondrial treatment with different Pyr and Pz compounds (10 μM), showing the effect of successive additions of 5 μM calcium (red arrows). Only one peak was seen with the vehicle dimethyl sulfoxide (DMSO), after which MPTP opening was observed, as shown by a sustained rise in the fluorescence of the membrane-impermeable reporter Calcium Green-5N with escape of the accumulated calcium from the mitochondrial matrix into the medium; 5 peaks were seen following treatment with CsA. (B) Bar graph showing the number of peaks obtained upon calcium addition until a sustained increase in fluorescence signal was observed by Pyr and Pz compounds (10 μM), showing marked increases in effect with progression through the Pz series. CRC_0_ represents CRC of mitochondria in the presence of DMSO; *n* ≥ 3. (C) Concentration-dependent CRC responses of **18f**, tested at 8 different concentrations from 39 nM to 5 μM. (D) CRC of isolated mitochondria examined in the absence or presence of inhibitors, each at 300 nM concentration; *n* ≥ 3; statistical notation denotes comparison to CsA*p < 0.05; **p < 0.01. (E) Specificity of CypD inhibition as a mechanism preventing MPTP opening was assessed using mitochondria isolated from CypD genetic knockout (*Ppif* ^*–/–*^) mice challenged repeatedly with calcium. *Ppif* ^*–/–*^ mitochondria, in the absence of any inhibitor, were resistant to MPTP opening; no further increase in mitochondrial CRC was observed in the presence of any inhibitor tested.

To test whether the compounds maintain mitochondrial integrity by inhibition of mitochondrial CypD, CRC assays were performed using mitochondria isolated from constitutive genetic CypD knockout mice (*Ppif* ^*–/–*^) that do not express CypD.^4^ Neither CsA nor representative Pyr (**3**) or Pz (**12a**, **13**, **18b**, and **18c**) compounds increased the CRC of isolated *Ppif* ^*–/–*^ mitochondria, as expected from CypD inhibition as the sole mechanism preventing MPTP opening by either genetic knockout (*Ppif* ^*–/–*^) or Pz compounds (Figure 5E).

The impact on necrotic cell death of Pz compounds with the greatest affinity for CypD was examined following taurolithocholic acid 3-sulfate (TLCS, 500 μM) exposure of freshly isolated murine pancreatic acinar cells (PACs), an initial site of injury in pancreatitis.^27^ Informed by our prior data, we selected **18b**, **c**, and **d** to evaluate their effects and compared them with CsA in a concentration-dependent manner (Figure 6). TLCS depolarizes mitochondria and induces necrosis of PACs from loss of ATP production.^4,28,29^ As shown in Figure 6, analogues **18b**–**d**, with all the concentrations tested, significantly reduced necrotic cell death pathway activation in PACs, including at the lowest concentration tested (0.1 μM).; Notably, compared to CsA, **18b**, **c** showed greater reduction in cell necrosis at the lowest concentration (0.1 μM). Furthermore, protective effects of Pz compounds were not restricted to bile acid-induced injury; **18c** inhibited necrotic cell death in PACs induced by palmitoleic acid, a product of ethanol nonoxidative metabolism that induces damage via Ca^2+^-dependent mitochondrial depolarization and ATP loss (data not shown).^30^

**Figure 6:**
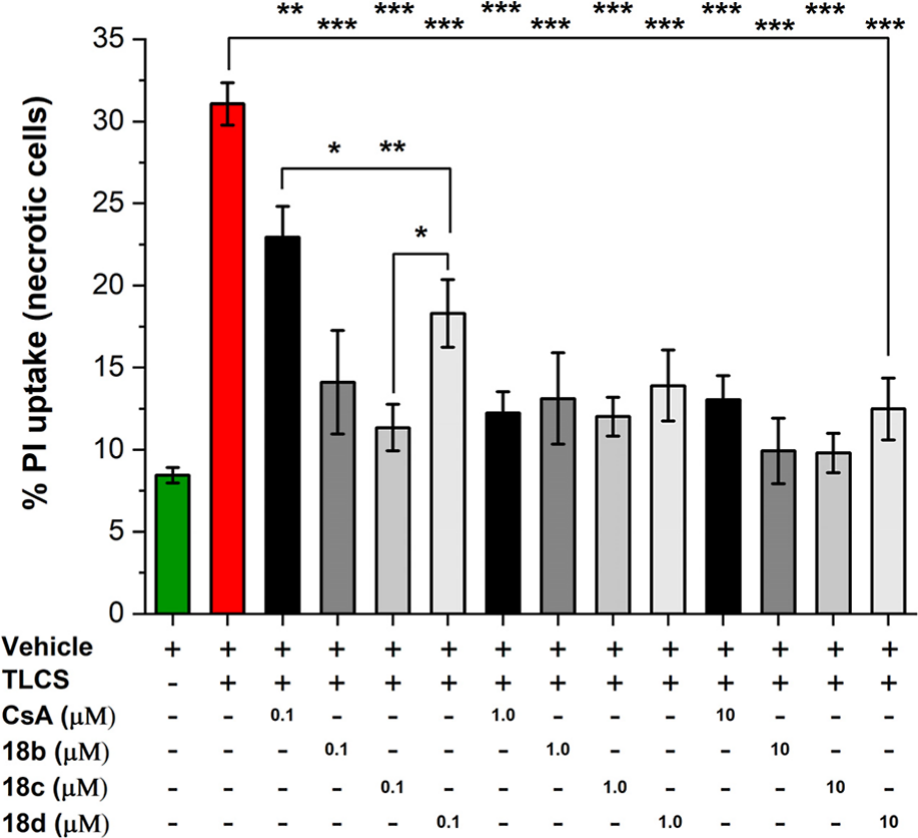
Inhibition of necrotic cell death induced by taurolithocholic acid 3-sulfate (TLCS, 500 μM) following treatment of freshly isolated murine pancreatic acinar cells with CsA, **18b**, **18c**, or **18d** (0.1–10 μM); mean ± SEM, minimum 3 experiments/group; **p < 0.01; ***p < 0.001 vs TLCS alone. Cells were loaded with propidium iodide (PI), uptake of which was assessed by confocal microscopy, to measure necrotic cell death pathway activation.

Compounds **18c** and **18f** were the most effective compounds in the CRC assay. As both were found to have favorable *in vitro* metabolic profiles with low rat hepatocyte clearance, these compounds were carried forward to *in vivo* PK assessment. Compounds were given by intraperitoneal (IP) administration as a single 20 mg/kg dose in male CD1 mice, and free plasma concentrations were monitored over a 24 h period. Experiments were performed in triplicate. The results are summarized in Figure 7.

**Figure 7:**
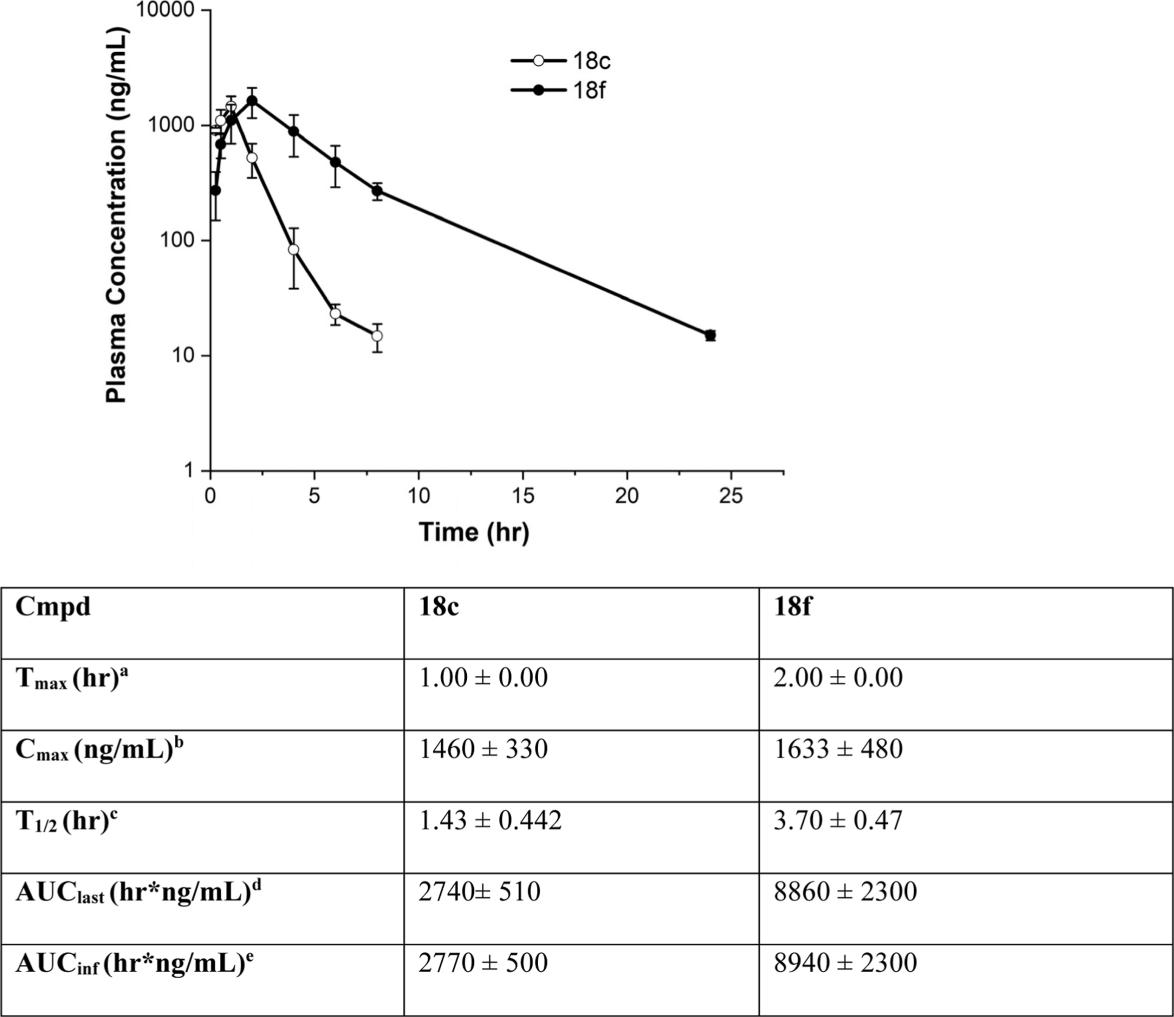
Mean plasma concentration–time profiles of **18c** and **18f** after single-dose administration in male CD1 mice and a summary of *in vivo* PK measurements. Results are for single 20 mg/kg IP dose administration in male CD1 mice. Experiments were carried out in triplicate. Results are presented as mean ± standard deviation; ^*a*^time to reach maximum plasma concentration; ^*b*^maximum plasma concentration; ^*c*^plasma elimination half-life; ^*d*^area under the curve to last measurable time; and ^*e*^area under the curve to infinity.

After IP administration, **18c** was rapidly absorbed, reaching a *C*_max_ of 1460 ng/mL at 1 h, followed by rapid elimination with a half-life of 1.43 h and dropping below detectable levels by 8 h. In contrast, **18f** was absorbed more slowly, reaching a *C*_max_ of 1633 ng/mL at 2 h, and was eliminated more slowly with a 2.5-fold lower half-life of 3.7 h. The *in vivo* pharmacokinetic profile of **18f** was supportive of further *in vivo* investigations in animal models of acute pancreatitis.

To evaluate the effects of CypD inhibition on ameliorating acute pancreatitis in mice, **18f** was administered in the caerulein model (CER-AP).^31^ The dosage was determined based on the PK data of **18f** in mice (Figure 7). CER-AP was induced by a series of 7 hourly intraperitoneal (IP) CER injections (50 μg/kg) were performed in wild-type C57BL6/J male and female mice (10–12 weeks old). The inhibitor **18f** was administered IP at a dose of 20 or 50 mg/kg immediately after the third and sixth and 2 h after the seventh CER injection (at the end of the second, fifth, and eighth hour of the experiment). Animals were humanely sacrificed 12 h after the first CER injection for collection of pancreas tissue and blood. Serum amylase and pancreatic trypsin activity, myeloperoxidase (MPO), edema, inflammation, and necrosis are elevated in acute pancreatitis and used as biomarkers to determine severity in animal models.^4^ Amylase, MPO, and overall histology score raised by CER were significantly reduced in animals treated with **18f** with three doses of 50 mg/kg (Figure 8). Trypsin also showed a marked reduction in the concentration in the presence of **18f**.

**Figure 8:**
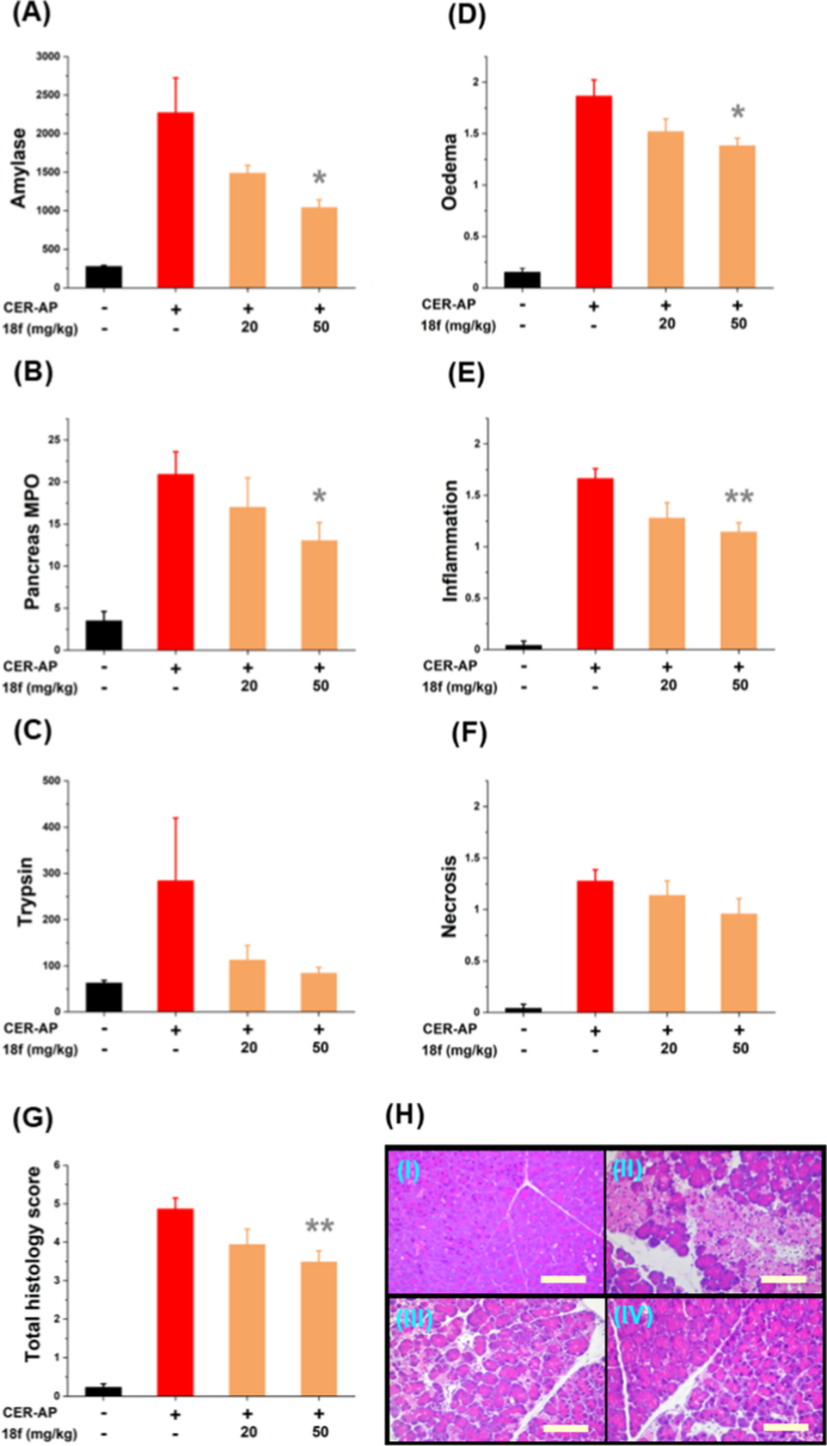
Significant reduction in the severity of CER-AP by **18f**. (A) Serum amylase (U/L), (B) pancreatic MPO activity (C) trypsin activation, (D) histology score for edema, (E) inflammation, (F) necrosis, (G) total histology score, and (H) representative histology images of the pancreas tissue of four groups of mice [(I) control, (II) CER-AP, (III) CER-AP + 20 mg/kg **18f**, and (IV) CER-AP + 50 mg/kg **18f**]; the scale bar represents 100 μm. For each group ≥6 mice were used.

## Discussion and Conclusions

Using a conformational restriction strategy, we developed potent small-molecule CypD inhibitors with low nanomolar activity and good drug-like properties. These compounds effectively prevent MPTP opening and necrotic cell death, matching or exceeding CsA. Key structural features—RHS Pz ring, backbone phenylglycine, and LHS glycan—were essential, each significantly enhancing performance through iterative optimization.

Grädler *et al.* identified low-affinity CypD-binding fragments, including a glycan-fused aniline binding in the S2 pocket with a *K*_i_ of 1.1 mM. Incorporating this glycan into the LHS of the chiral-Pyr scaffold significantly enhanced potency.^23^ Replacing the aniline to remove the potential bioactivation risk to mutagenic metabolites has often reduced activity.^17,32,33^ In the current study, glycan-fused LHS substitution in compounds **12a**, **12c**, and **12d** (yielding **18a**–**18c**) resulted in marked increases in *K*_i_ and *K*_d_ values, mirroring earlier gains seen with Pyr analogues. Additional analogues **18d** and **18e**, incorporating phenylglycine, were compared to their parent aniline and glycan-glycine counterparts (**13** and **18c**, respectively). These showed slightly reduced potency, indicating that the benefits of phenylglycine and glycan-fused LHS are not synergistic. Structural insights from the crystal structure of **18d** revealed an expanded S2 pocket that accommodates extra stabilizing interactions, specifically hydrogen bonding and hydrophobic contacts, which likely account for the observed potency enhancement (Figure S6).

We used the CRC assay to evaluate how well compounds protected murine liver mitochondria from calcium-induced MPTP opening using CsA as a reference. While Pz anilines **12a–d** showed notable improvements in PPIase inhibition and binding, they offered little CRC improvement at 10 μM. In contrast, phenylglycine compound **13** and glycan-fused analogues **18b–e**, which had nanomolar potency in PPIase and ITC assays, showed significant CRC enhancement. Notably, compounds **18d** and **18f** provided significantly superior protection at 300 nM compared to that of CsA, with **18f** being the most effective. The parallel between CypD binding affinity (*K*_i_ and *K*_d_) and CRC is consistent with MPTP inhibition occurring via CypD targeting. Additionally, CRC assays using Ppif^–/–^ mitochondria showed no change, confirming that the observed protective effects are specifically due to CypD inhibition rather than off–target interactions. Necrotic cell death is key to MPTP-related diseases;^1,2,4–6^ Glycan-fused inhibitors **18b–d** and CsA were tested for their ability to reduce necrosis in murine PACs. Compounds significantly reduced necrosis across 0.1 and 10 μM. Although compounds **18b** and **18c** provided similar protective effects in PACs, **18c** displayed significantly greater protection compared to CsA at 0.1 μM.

DMPK screening showed that incorporating glycan-fused LHS significantly improved solubility and metabolic stability. Compound **18c** exhibited the highest solubility (>1000 μM), attributed to the polar groups in the glycan, with corresponding log *D* reductions of 0.2–0.4 units. Metabolic stability improved likely due to the removal of the metabolically unstable aniline group. Cyp3A4 metabolism modeling confirmed this, predicting up to 75% metabolism for the aniline nitrogen, dropping to as low as 1% in glycan-fused analogues (Table S3). Between **18c** and **18f** the latter was prioritized for in vivo animal model of acute pancreatitis testing due to its 3-fold higher exposure.^34^

Finally, in the CER-AP model, compound **18f** was evaluated for ameliorative effects.^29,35^ We found that in all cases administration of **18f** reduced biomarkers of acute pancreatitis compared to the mean value for CER-AP positive control mice. While 20 mg/kg showed no significant benefit, administration at 50 mg/kg led to significant reductions in amylase activity and pancreatic MPO and histological scores for edema and inflammation. The total histological score was also significantly improved compared to the control. These *in vivo* findings confirm the ameliorative effects of **18f** in animal models and, alongside its mitochondrial and acinar cell protection, validate the effectiveness of the novel synthetic strategy used to develop this compound.

In conclusion, we have demonstrated that Pz-containing inhibitors have superior potency and DMPK profiles compared to their Pyr-containing counterparts, especially glycan-fused analogues **18b**–**18f**. *In vivo* profiling of **18f** showed an acceptable PK profile in mice and significant improvement of acute pancreatitis biomarkers in the CER-AP mouse model at 50 mg/kg IP dosing. These data show clear potential for the Pz-containing inhibitors, justifying the further development of this CypD inhibitor scaffold. Future work will engage in modification of current lead compounds to enhance CypD subtype selectivity, recently shown by Liu and co-workers with macrocyclic-based cyclophilin inhibitors^36^ and Michel for trivector-based ureas targeting cyclophilin B.^37^

## Experimental Section

### General Synthetic Procedures

Full details of the synthesis of analogues are included in the Supporting Information. Compounds screened were >95%, as assessed by HPLC or by CHN analyses.

#### Recombinant CypD Preparation

Recombinant CypD was expressed in *E. coli* and purified using a protocol previously described.^19^

#### Prolyl-Peptidyl Isomerization Assay

For compounds **13–19**, PPIase assays were performed by Eurofins Integrated Discovery UK Ltd. Using the method described by Jankowski et al.^38^

Assay buffer (1.5 mL of 35 mM HEPES pH 7.8 with 50 μM DTT) in a 3 mL precision glass cuvette is cooled to 10.0 ± 0.1 °C with stirring (vigorous but not so fast as to produce cavitation). The inhibitor is diluted in 100% DMSO and then added to the assay to a maximum concentration of 0.5% DMSO final concentration in the assay. A blank spectrum is obtained, and then enzyme (2 nM final concentration) and substrate (60 μM final concentration) are added. The absorbance is measured at 330 nm for 300 s. A first-order rate equation is fitted to the absorbance data, for each concentration of inhibitor, to obtain the rate constant (the first 10 to 15 s were excluded as mixing causes errors in this portion of the curve). The catalytic rate is calculated from the enzymatic rate constant minus the background rate constant. The enzymatic rate constant, determined in duplicate at each inhibitor concentration, was plotted against inhibitor concentration, and a nonlinear fit by SigmaPlot software generated the *K*_i_.

For compounds **4a**–**12d** the in-house method described below was used.

The activity of CypD was monitored spectrophotometrically using a chymotrypsin-coupled assay method. A 60 μM Suc-AAFP-pNA substrate concentration was used to determine the activity of 2 nM CypD in 50 mM HEPES pH 7.5, 25 mM NaCl at 10 °C. *K*_i_ values were determined by measuring isomerase activity in the presence of 0–10 μM of inhibitor. The typical total volume of each reaction was 250 mL, and the reaction was performed in disposable microcuvettes. Each 2 nM CypD reaction containing an appropriate concentration of the inhibitor (from a 100 mM stock in DMSO) was pre-equilibrated for 15 min at 10 °C. A freshly prepared room temperature stock of 15 mg mL^–1^ (approximately 0.50 mM) alpha-chymotrypsin in 1 mM HCl was added to give a final reaction concentration of 50 mM before absorption data collection at 390 nm was initiated. The peptide substrate Suc-AAPF-pNA was then immediately added to a final concentration of 60 mM from a 10 mM stock of the peptide dissolved in 0.47 M LiCl/TFE. The final reaction was monitored for 2.5–5 min. Data collection and exponential fitting of the absorbance reaction curve after initial mixing were performed on a Varian Cary 300 Bio UV–vis Spectrophotometer. Inhibition constants were calculated by nonlinear regression curve fitting through Origin (OriginLab, Northampton, MA).

#### X-ray Crystallography

Compounds **12a** and **18d** were solubilized in 100% ethanol and incubated with CypD (25 mg/mL) in a 2–1 molar excess. Sitting drop vapor experiments were set up at 18 °C using 400 nL drops with a 1:1 precipitant-precipitate ratio. Crystals typically appeared for all complexes in many conditions in ∼24 h, and prior to data collection, crystals were vitrified in mother liquor containing 20% ethylene glycol. X-ray data for the structure of the CypD-**12a** complex were collected using synchrotron light at the Diamond Light Source (beamline I03) to 1.45 Å resolution. X-ray data for the CypD-**18d** complex were collected at a longer wavelength at the Barkla X-ray Laboratory of Biophysics (University of Liverpool) using a Rigaku FR-E+ SuperBright rotating-anode generator and an EIGER R 4 M detector to 2.25 Å resolution. Both structures were solved by molecular replacement for **12a** PHASER5, and the template structure PDB: 5CBT was used, while for **18d**, we used MOLREP^40^ and PDB: 4O8H as a starting model. The models, improved by iterative refinement and building using Phenix^39^ (REFMAC5 ^41^) for **12a**(**18d**) and model building in COOT,^42^ clearly demonstrated the presence of the ligands in the *F*_0_–*F*_C_ map (contoured at 3.5σ) and the 2*F*_0_–*F*_C_ map (1σ), allowing unambiguous assignment of ligand binding modes. Data collection and refinement statistics are shown in Table S4, and data were deposited to the PDB with the accession codes 9H0S and 9HTR prior to publication. A summary of noncovalent interactions is provided in Tables S5 and S6 for compounds **12a** and **18d**.

#### Isothermal Titration Calorimetry

All ITC experiments were carried out at 25 °C on a PEAQ-Microcalorimeter (Malvern). The buffer used was 20 mM phosphate, 20 mM NaCl at pH 6.5. Each experiment consisted of an initial injection of 0.5 μL, followed by 15, 2.39 μL injections before a final injection of 1.89 μL. Control experiments were performed whereby each compound was titrated into buffer and buffer titrated into CypD. Heats of dilution effects were subtracted for the majority of the experiments. The titration experiments were performed in duplicate with the exception of compounds **13** and **4c**, with 50–100 μM CypD in the cell and 500–2000 μM compounds in the syringe with 1% DMSO present. For some of the titrations, data from two runs were concatenated to achieve a saturating isotherm. All data was analyzed using the MicroCal PEAQ-ITC Analysis software program (Malvern Instruments).

All animal studies described below were approved by the University of Liverpool Animal Welfare Committee and Ethical Review Body (AWERB). (Home Office Project License PDC14C46E, Pancreatic Digestive Diseases.)

#### Isolation of Mitochondria

10–12 week old CD1 mouse liver mitochondria were prepared by standard differential centrifugation.^43^ Mouse liver was removed and placed in ice-cold isolation buffer (250 M sucrose, 10 mM Tris-HCl, 0.1 mM EGTA-Tris, pH 7.4) supplemented with BSA. Liver tissue was then cut into small pieces and transferred to a prechilled Potter homogenizer with a Teflon pestle. The homogenate (∼30 mL/liver) was centrifuged at 700*g* for 10 min at 4 °C. The supernatant containing mitochondria and other organelles was transferred to new tubes and centrifuged at 6000*g* for 10 min at 4 °C. The supernatant was discarded, and the mitochondrial pellet was carefully suspended in ice-cold IB buffer and spun at 9500*g* for 5 min at 4 °C. The pellet was suspended in isolation buffer and placed on ice. Protein concentration was determined by the Biuret method.

### Cell Isolation Procedure

The PACs were isolated by the standard collagenase digestion procedure using purified collagenase (200 units mL^–1^), as described previously.^4,19^ The extracellular solution contained (mM): 140 NaCl, 4.7 KCl, 1.13 MgCl_2_, 1.0 CaCl_2_, 10 d-glucose, and 10 HEPES (adjusted to pH 7.35 using NaOH). All experiments on isolated PACs were performed no more than 4 h after isolation.

### Assessment of CRC

CRC of isolated mouse liver mitochondria was assessed as follows: First, 100 μL of assay buffer (250 mM sucrose, 10 mM MOPS-Tris, 0.01 mM EGTA-Tris, 1.0 mM phosphoric acid-Tris, 10 mM glutamate, and 5 mM malate, pH 7.4) was dispensed to a 96-well black assay plate in the absence or presence of an inhibitor. Then, 100 μL of 0.25 mg/mL mitochondria supplemented with 0.5 μM Calcium Green-5N were added to the plate. Mitochondria were challenged with the addition of Ca^2+^ (5 μM each time) at regular intervals, and fluorescence intensity (excitation 485 nm, emission 530 nm) was read on a POLARstar Omega (BMG Labtech) plate reader.

### Necrotic Cell Death Measurement

Freshly isolated PACs were treated with vehicle (0.5% DMSO), TLCS (500 μM), or TLCS together with Pz inhibitor (0.1, 1.0, or 10 μM) for 30 min at room temperature with gentle shaking. After washing, fluorescent dye propidium iodide (PI, a marker of necrosis; λ_ex_/λ_em_, 540/620 nm) was added (10 μg/mL final concentration) to the cells to assess plasma membrane rupture. Cells were distributed in 96 well glass-bottom plates (200 μL/well) and imaged using the Carl Zeiss LSM710 system with Zen software.^19^ For each group, 12 randomly selected fields of view (each field contained mostly over 100 cells) were taken of each mouse isolate, and the total number of cells displaying PI uptake was counted per field to give a percentage ratio for each field, averaged across fields, and converted to a mean and standard error of the mean for a minimum of three mice per experimental group. The experiment was performed in a blinded fashion in such a way that the observer choosing the imaging fields and the observer undertaking image analysis were unaware of the treatment groups.

### Experimental Mouse CER-AP Model

CER was originally derived from the skin of the Australian frog, Litorea caerulea, and is an analogue of cholecystokinin. The most reliable secretagogue hyperstimulation CER-AP model has been widely used in >4000 studies during the last 40 years.^29,35^ The experimental CER-AP was induced in male and female mice (10–12 weeks old) by 7 hourly intraperitoneal (IP) injections of 50 μg/kg Cer; controls received saline. **18f** dissolved in saline containing 10% Cremophor and 10% dimethyl sulfoxide was IP injected (20 or 50 mg/kg) at the end of the second, fifth, and eighth hour of the experiment. For maintaining an appropriate level of **18f** in mice during the 12 h CER-AP model, the dosage was decided based on the half-life of **18f** in the PK study data (Figure 7). Sacrifice of animals was made 12 h after the first injection of the CER.

### Histological Analysis and Biochemical Measurements for the Severity of Acute Pancreatitis

Histological analysis and biochemical measurements of acute pancreatitis in pancreatic tissues were made at 12 h after the first injection of CER. For histology, tissues were fixed in 10% formalin, embedded in paraffin, and stained with hematoxylin and eosin (H & E). Histological scoring was performed on 10 random fields by 2 blinded investigators independently grading edema, inflammatory cell infiltration, and acinar necrosis (scale, 0–3),^35^ and data presented as the mean ± SEM (≥6 mice/group), as described previously.^4,29^ Pancreatic trypsin activity was determined with an established protocol using the trypsin peptide Boc-Gln-Ala-Arg-MCA substrate (Peptides International, Louisville, KY, USA) with excitation at 380 nm and emission at 440 nm. Serum amylase was determined using a Roche Analyzer; myeloperoxidase (MPO) activity was measured according to an established protocol using a plate reader, calculated as the difference between 0 and 3 min at an absorbance wavelength of 655 nm.^4,29^

## Computational

Computational procedures are outlined in the Supporting Information section.

## Supplementary Material





## Notes

SMILES of compounds (CSV)
General chemistry methods, chemistry procedures, analytical data/ NMR/HPLC traces, compound 4a binding to CypD with key interactions highlighted, superposition of CypD X-ray crystal structures with compounds 12a and 4a showing an identical ligand binding mode, structure of compound 13 docked into CypD (PDB 4J5D) by the molecular docking package GOLD, ITC profiles for the binding of selected compounds to CypD, thermodynamic signatures, Cyclophilin D (CypD) peptidyl-prolyl isomerase (PPIase) assay, additional NMR studies, synthesis of Pyr analogues 4a-d, synthesis of biaryl-methylmorpholine analogue 18f, and calculated energies of conformers of the model Pyr 1a (PDF)

## Abbreviations

Aza-pro: Aza-proline
k_d_: Binding constant
CER-AP: caerulein acute pancreatitis
CsA: Cyclosporin A
CypD: Cyclophilin D
CRC: calcium retention capacity
IP: intraperitoneal
ITC: Isothermal calorimetry
*K*_i_: inhibition constant
LHS: left-hand side
Log D: Distribution constant
MPTP: Mitochondrial Permeability Pore
MPO: myeloperoxidase
NBO: Natural bonding orbitals
PACS: pancreatic acinar cells
PPIase: Peptidyl Prolyl Isomerase
PK: pharmacokinetics
Pyr: Pyrrolidine
Pz: Pyrazolidine
Rat Heps Clint: rat hepatocyte clearance
RHS: right-hand side
H. Mics. Clint.: human microsomal clearance
