# Supplementary material for: Potent Preorganized Pyrazolidine Cyclophilin D Inhibitors Prevent Mitochondrial and Organ Injury in a Mouse Pancreatitis Disease Model
Source: J Med Chem. 2025 Nov 10;68(22):23910–24. doi: 10.1021/acs.jmedchem.5c01146 (PMC12670411; doi:10.1021/acs.jmedchem.5c01146)
Supplement: Supplementary file 2 [file jm5c01146_si_002.pdf]

Supporting Information for:

Potent pre-organised pyrazolidine cyclophilin D inhibitors prevent mitochondrial and organ injury in a mouse pancreatitis disease model

Muhammad Awais,[a] Christopher M. Woodley,[b] Liqun Guo,[b] Michael Rogers,[b] Neil Kershaw,[b] Thomas Zacharchenko,[d] Emma Shore,[b] Arjun Kattakayam,[c] Rajarshi Mukherjee,[c] David N Criddle,[a] Suet C. Leung,[b] Heather Lee,[b] Joshua Burgess-McCann,[b] Konstantin Luzyanin,[b] Neil G. Berry,[b] Svetlana Antonyuk,[a] Lu-Yun Lian,[a] Robert Sutton[c]* and Paul M. O'Neill[b]*

[a]Institute of Systems, Molecular and Integrative Biology, University of Liverpool, Liverpool, UK, L69 7BE

[b]University of Liverpool, Chemistry, Robert Robinson Laboratories, Oxford Street

Liverpool, UK L69 7ZD

[c]Liverpool Pancreatitis Research Group, Liverpool University Hospitals NHS Foundation Trust, Liverpool, UK, L7 8XP

[d]Wellcome Centre for Cell-Matrix Research, The University of Manchester, Michael Smith Building | Dover Street | M13 9PT

Corresponding author e-mail; pmoneill@liverpool.ac.uk

Table of Contents

1. General Chemistry Methods Page S2
2. Chemistry Procedures Page S3
3. Analytical Data/ NMR/HPLC traces Page S37
4. Supporting Figures Page S65
5. Additional NMR Studies Page S73
6. Supporting Schemes Page S80
7. Supporting Tables Page S81
8. Supporting Methods Page S89
9. References Page S90

1. General Chemistry Methods

Unless stated, all starting materials were purchased from commercial sources (Sigma Aldrich, Fluorochem, Thermo Scientific Chemicals, LCC or Apollo) and used without any further treatment. Flash column chromatography (FCC) was performed using silica gel (Aldrich 40–63 µm, 230–400 mesh). Thin layer chromatography (TLC) was performed using aluminium backed 60F254 silica plates. Visualization was achieved by UV fluorescence, $KMnO_4$ solution and heat or 4 wt.% ninhydrin solution in ethanol and heat. Proton nuclear magnetic resonance (NMR) spectra were recorded at 400 MHz or 500 MHz at 298K unless stated otherwise. ^{13}C NMR spectra were recorded at 100 MHz or 125 MHz at 298K unless stated otherwise. Chemical shifts (δ) are given in parts per million (ppm) and are listed downfield with tetramethylsilane as a reference. Peaks are described as singlets (s), doublets (d), triplets (t), quartets (q), quintets (quint), multiplets (m) and broad (br.). Coupling constants (*J*) are quoted to the nearest 0.1 Hz. Reported NMR chemical shifts, integration and coupling constants are based on 1D NMR data. Assignments of major and minor isomeric forms are informed by NOE experiments and *J* coupling values.

Mass spectra were recorded on Agilent QTOF 7200 and Micromass LCT mass spectrometers. For chemical and electrospray ionisation modes ammonia or methanol were used as solvent systems respectively. Microanalyses (%C, %H, %N) were carried out in the University of Liverpool Microanalysis Laboratory. Purity determination was performed by HPLC analysis using Agilent 1200 solvent delivery system. The HPLC methods used the following conditions: ZORBAX Eclipse Plus C18 (4.6 mm × 100 mm, 3.5 µm) at 25 °C with 1.0 mL/min flow rate. Solvent – A) water containing 0.1% formic acid and B) acetonitrile containing 0.1% formic acid; Method (Acidic, 2-98%): Run time: 15 min, gradient: 2% B holds to 1 min, 2-98% B in 11 min, then holds at 98% B to 15min. Enantiomeric purity for compound **13** was determined by chiral HPLC using the following conditions: DAICEL CHIRALCEL OD-H (4.6 mm x 250 mm, 5 µm) at 25°C with 1.0 mL/min flow rate. Solvent – A) hexane and B) isopropanol: Run time: 22 min, gradient: 70-90% B in 10 min, then holds at 90% B for 12 min. Melting points were determined on a Gallenkamp melting point apparatus in degrees Celsius and are uncorrected.

Compound **4a** was prepared according to Shore et al.[1].

2. Chemistry Procedures

(*R*)-1-(2-(2,5-Dichlorophenyl)pyrrolidin-1-yl)ethan-1-one **(1a)**

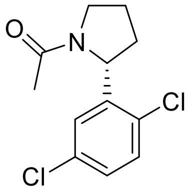

To a solution of (*R*)-2-(2,5-dichlorophenyl)pyrrolidin-1-ium chloride (75.00 mg, 0.30 mmol, 1.00 eq) in DCM (0.2 M, 1.50 ml), pyridine (0.05 ml, 0.60 mmol, 2.00 eq) was added followed by addition of acetic anhydride (0.05 ml, 0.60 mmol, 2.00 eq). The reaction mixture was allowed to stir at room temperature overnight before being concentrated in vacuo and taken up in EtOAc (5 ml), washed with 1M HCl (10 ml) and sat. Na_2CO_3 solution (10 ml) then dried over then dried over MgSO4. The crude product was purified by FCC (SiO_2; 50:50 EtOAc:hexane) to yield a white solid (66.1 mg, 86%). With the yield consisting of a mixture of rotamers in ratio 1.1/1 trans/cis. ^{1}H NMR (400MHz; CD_2Cl_2; Me_4Si; ppm; 213K) δ 7.36 (minor, d, J = 8.5 Hz, 1H), 7.33 (major, d, J = 8.5 Hz, 1H), 7.25 (major, dd, J = 8.5, 2.5 Hz, 1H), 7.20 (minor, dd, J = 8.5, 2.5 Hz, 1H), 7.13 (minor, d, J = 2.2 Hz, 1H), 7.00 (major, d, J = 2.0 Hz, 1H), 5.24 (major, ap: d, J = 8.3 Hz, 1H), 5.15 (minor, ap: d, J = 8.1 Hz, 1H), 3.83-3.72 (major/minor, m, 4H), 3.70-3.3.60 (major/minor, m, 4H), 2.48-2.19 (major, m, 2H), 2.14 (major, s, 3H), 2.01-1.66 (minor, m, 2H), 1.80 (minor, s, 3H). ^{13}C NMR (100 MHz; $CDCl_3$; Me_4Si) δ 170.1, 169.3, 142.2, 142.1, 133.4, 132.7, 131.1, 131.0, 130.3, 129.9, 128.8, 128.0, 126.5, 126.0, 59.6, 58.3, 48.7, 47.1, 34.0, 32.2, 23.6, 22.8, 22.4, 21.8. HRMS (ES+) 280.0266 $[M+Na]^+$ $C_{12}H_{13}NO^{35}Cl_2Na$ requires 280.0266 (Diff -2.29 ppm).

1-(2-(2,5-Dichlorophenyl)pyrazolidin-1-yl)ethan-1-one **(1b)**

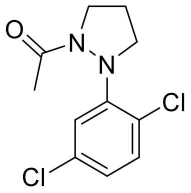

Tert-butyl-2-(2,5-dichlorophenyl)pyrazolidine-1-carboxylate (203.00 mg, 0.64 mmol, 1.00 eq) was dissolved in 5:1 DCM:TFA and the reaction mixture was allowed to stir at room temperature until consumption of the starting material was observed by TLC. The solvent was removed *in vacuo* to afford 2-(2,5-dichlorophenyl)pyrazolidin-1-ium 2,2,2-trifluoroacetate. The crude product was carried through to the next step.

To a solution of 2-(2,5-dichlorophenyl)pyrazolidin-1-ium 2,2,2-trifluoroacetate (212.00 mg, 0.64 mmol, 1.00 eq) in DCM (0.20 M, 1.50 ml), pyridine (0.06 ml, 0.80 mmol, 1.02 eq) was added

followed by addition of acetic anhydride (0.10 ml, 1.00 mmol, 1.60 eq) and DMAP (8.00 mg, 0.06 mmol, 0.10 eq). The reaction mixture was allowed to stir at room temperature overnight before being concentrated in vacuo and taken up in EtOAc (5 ml), washed with 1M HCl (10 ml) and sat. Na_2CO_3 solution (10 ml) then dried over then dried over $MgSO_4$. The crude product was purified by FCC (SiO_2; 20:80 EtOAc:hexane) to yield a brown solid (70 mg, 42%). ^{1}H NMR (400MHz; CD_2Cl_2; Me_4Si; ppm; 213 K) δ 7.38 (d, J = 9.1 Hz, 1H), 7.10-7.06 (m, 2H), 3.87 (ap: q, J = 10.4 Hz, 1H), 3.87 (ap: t, J = 10.2 Hz, 1H), 3.59 (ap: dd, J = 11.7, 7.0 Hz, 1H), 3.38 (ap: td, J = 12.0, 5.9 Hz, 1H), 2.21-2.09 (m, 1H), 2.06-1.94 (m, 1H), 2.00 (s, 3H). 13C NMR (100 MHz; $CDCl_3$; Me_4Si) δ 172.7, 148.9, 133.3, 131.8, 125.0, 123.5, 119.3, 55.2, 44.4, 24.0, 21.2. HRMS (CI) 259.0402 $[M+H]^+$ $C_{11}H_{13}$N2O $^{35}Cl_2$ requires 259.0399 (Diff -1.12 ppm).

Compounds **4b-4d**

Compounds **4b-4d** were prepared from chiral pyrolidines, (*R*)-2-(2,5-dichlorophenyl)pyrrolidine hydrochloride, (*R*)-2-(2-chloro-5-methoxyphenyl)pyrrolidine hydrochloride and (*R*)-2-(2-chloro-5-fluorophenyl)pyrrolidine hydrochloride. The first was purchased from Fluorochem (95%ee) and the latter two were from LCC (>95% ee). Chiral HPLC traces from the starting materials supplied by LCC are shown in the HPLC traces section.

N-(4-Aminobenzyl)-1*H*-imidazole-1-carboxamide

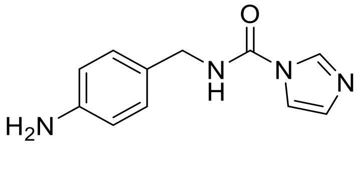

4-Aminobenzylamine (0.46 ml, 4.09 mmol, 1 eq) was dissolved in DCM (41 mL) and the reaction mixture was allowed to cool to 0 °C. CDI (730 mg, 4.50 mmol, 1.1 eq) and DMAP (50 mg, 0.41 mmol, 0.1 eq) were added and the reaction mixture was allowed to stir at r.t. overnight. After this time, the reaction mixture was diluted with EtOAc (10 mL) and washed with water (30 mL). The organic layer was dried over $MgSO_4$, filtered and concentrated *in vacuo* to yield a yellow solid that was used without further purification (849 mg, 3.93 mmol, 96%). ^{1}H NMR (400 MHz; CD_3OD; Me_4Si; ppm) δ 8.21 (1H, s), 7.57 (1H, s), 7.07 (2H, d, *J* = 8.3), 6.99 (1H, s), 6.65 (2H, d, *J* = 8.3), 4.34 (2H, s); ^{13}C NMR (100 MHz; CD_3OD; Me_4Si) δ 149.2, 148.4, 136.4, 130.0, 129.0, 125.7, 117.0, 114.2, 43.8.

Spectroscopic data are in agreement with the literature.[1]

***tert*-Butyl (*R*)-(2-(2-(2,5-dichlorophenyl)pyrrolidin-1-yl)-2-oxoethyl)carbamate**

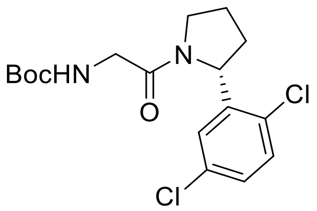

To a solution of (R)-2-(2,5-dichlorophenyl)pyrrolidine hydrochloride (0.10 g, 0.40 mmol) in anhydrous DMF (7.5 mL) was added boc-glycine (83 mg, 0.48 mmol, 1.2 eq), DIPEA (0.17 mL, 1 mmol, 2.5 eq) and HATU (0.20 g, 0.52 mmol, 1.3 eq). The reaction mixture was allowed to stir at room temperature overnight under N_2 (followed by TLC). Upon completion, the solution was diluted with EtOAc and washed with sat. aq. $NaHCO_3$, water and brine. The organic layer was dried over $MgSO_4$ and concentrated *in vacuo*. The crude product was purified by FCC (SiO_2; 20:80 EtOAc:Hexane) to afford the title compound as a brown foam (0.13 g, 85%). With the yield consisting of a mixture of rotamers in ratio 1.2:1. ^{1}H NMR (400MHz; $CDCl_3$; Me_4Si; ppm) δ 7.33 (minor, d, *J* = 8.6 Hz, 1H), 7.29 (major, d, *J* = 8.4 Hz, 1H), 7.22 (major, dd, *J* = 8.4, 2.1 Hz, 1H), 7.15 (minor, dd, *J* = 8.6, 2.0 Hz, 1H),7.14 (major, dd, *J* = 8.8 Hz, 1H), 7.03 (minor, d, *J* = 2.0 Hz, 1H), 6.93 (major, d, *J* = 2.1 Hz, 1H), 5.47 (br s, NH) 5.40 (major, dd, *J* = 8.4, 2.8 Hz, 1H), 5.20 (minor, app d, *J* = 6.8 Hz, 1H), 4.03 (major, s, 2H), 4.02 (minor, s, 2H), 3.99-3.29 (major & minor, m, 2H), 2.50-2.27 (major, m, 2H), 2.1-1.8 (minor, m, 4H), 1.44 (major, s, 9H), 1.41 (minor, s, 9H); ^{13}C NMR (100 MHz; $CDCl_3$; Me_4Si) δ 167.1, 156.0, 141.5, 133.0, 131.1, 129.1, 128.3, 126.0, 80.0, 58.9, 58.2, 50.9, 47.6, 46.8, 43.2, 34.3, 28.4 (3C), 23.6. ;HRMS (ES+) 395.0892 $[M+Na]^+$ $C_{17}H_{22}N_2O_3{}^{35}Cl_2Na$ requires 395.0905 (Diff -3.3 ppm).

(*R*)-1-(4-Aminobenzyl)-3-(2-(2-(2,5-dichlorophenyl)pyrrolidin-1-yl)-2-oxoethyl)urea (**4b**)

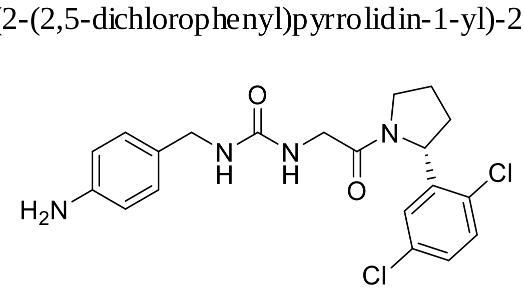

Tert-Butyl (*R*)-(2-(2-(2,5-dichlorophenyl)pyrrolidin-1-yl)-2-oxoethyl)carbamate (0.13 g, 0.33 mmol, 1 eq) was dissolved in a mixture of DCM:TFA (5:1, 8 mL) and the reaction mixture was allowed to stir at room temperature until TLC showed disappearance of starting material. The reaction mixture was concentrated *in vacuo* to afford the free amine as a trifluoroacetate salt and was used directly without further purification.

To a solution of the trifluoroacetate salt (0.33 mmol, 1 eq) in acetonitrile (8 mL), was added *N*-(4-Aminobenzyl)-1*H*-imidazole-1-carboxamide (79 mg, 0.37 mmol, 1.1 eq), triethylamine (0.10 mL, 0.73 mmol, 2.2 eq) and DMAP (3.8 mg, 0.03 mmol, 0.1 eq) and was allowed to stir at room temperature overnight under N_2 (followed by TLC). Upon completion, the solution was diluted with DCM and washed with distilled water. The organic phase was dried over $MgSO_4$ and concentrated *in vacuo*. The crude product was purified by flash column chromatography eluting ethyl acetate to yield a pale brown foam (70 mg, 50%) with the yield consisting of a mixture of rotamers in ratio 1.04:1. ^{1}H NMR (400 MHz, $CDCl_3$) δ 7.29 (rotamer 1, d, *J* = 8.5 Hz, 1H), 7.22 – 7.15 (rotamer 1 + 2, m, 1H), 7.07 (rotamer 2, dd, *J* = 8.5, 2.5 Hz, 1H), 7.04 – 6.95 (m, 2H), 6.92 – 6.86 (m, 1H), 6.66 – 6.52 (m, 2H), 5.95 (rotamer 1, dt, *J* = 61.2, 4.5 Hz, 1H), 5.61 (rotamer 2, dt, *J* = 68.5, 5.5 Hz, 1H), 5.28 (rotamer 1, dd, *J* = 8.3, 2.7 Hz, 1H), 5.16 (rotamer 2, dd, *J* = 8.1, 2.0 Hz, 1H), 4.35 – 3.96 (m, 4H), 3.90 – 3.43 (m, 3H), 3.38 – 3.14 (m, 2H), 2.38 – 2.14 (m, 1H), 2.01 – 1.55 (m, 3H); ^{13}C NMR (101 MHz, $CDCl_3$) δ 169.6, 168.9, 158.3, 158.1, 145.6, 145.6, 141.5, 141.0, 133.4, 132.9, 131.5, 131.1, 130.3, 130.2, 129.5, 129.3, 129.1, 128.9, 128.3, 126.0, 126.0, 115.2, 115.1, 58.9, 58.4, 47.4, 47.0, 44.1, 43.9, 43.2, 42.8, 33.9, 32.0, 23.5, 21.1. IR ν_{max}/cm^{-1}: 3338 (N-H), 1619 (urea C=O), 1514 (N-H), 813 (C-Cl). ; HRMS (ES+) 421.1185 $[M+H]^+$ $C_{20}H_{23}N_4O_2{}^{35}Cl_2$ requires 421.1198 (Diff -1.3 ppm); Anal. Cal. (mass %) for $C_{20}H_{22}Cl_2N_4O_2$: C, 57.02; H, 5.26; N, 13.30. Found: C, 56.77; H, 5.01; N, 13.05. m.p. 80-84°C.

Synthesis of Compound 4c

***Tert*-butyl*l*(R)-(2-(2-(2-chloro-5-fluorophenyl)pyrrolidin-1-yl)-2-oxoethyl)carbamate**

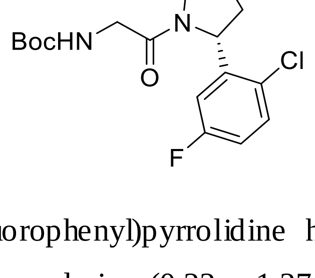

To a solution of (*R*)-2-(2-chloro-5-fluorophenyl)pyrrolidine hydrochloride (0.25 g, 1.06 mmol) in anhydrous DMF (7.5 mL) was added boc-glycine (0.22 g, 1.27 mmol, 1.2 eq), DIPEA (0.55 mL, 3.18 mmol, 3 eq) and HATU (0.48 g, 1.27 mmol, 1.2 eq). The reaction mixture was allowed to stir at room temperature overnight under N_2 (followed by TLC). Upon completion, the solution was diluted with

EtOAc and washed with sat. aq. $NaHCO_3$, water and brine. The organic layer was dried over $MgSO_4$ and concentrated *in vacuo*. The crude product was purified by FCC (SiO_2; 30:70 EtOAc:Hexane) to afford the title compound as a white foam (0.33 g, 87%). With the yield consisting of a mixture of rotamers in ratio 1.1:1. ^{1}H NMR (400 MHz, $CDCl_3$) δ 7.35 (dd, *J* = 8.6, 5.1 Hz, 1H, minor), 7.31 (dd, *J* = 8.7, 5.1 Hz, 1H, major), 6.98 – 6.92 (m, 1H, minor), 6.91 – 6.85 (m, 1H, major), 6.78 (d, *J* = 9.1 Hz, 1H, minor), 6.68 (dd, *J* = 9.1, 3.0 Hz, 1H, major), 5.39 (m, 2H), 5.19 (dd, *J* = 7.8, 1.2 Hz, 1H), 4.03 – 4.00 (m, 1H, minor), 3.95 (dd, *J* = 10.9, 6.3 Hz, 1H, major), 3.87 – 3.74 (m, 1H, minor), 3.79 – 3.71 (m, 2H, major), 3.63 – 3.54 (m, 1H), 3.34 – 3.26 (m, 1H), 2.50 – 2.25 (m, 3H, minor), 1.97 (m, 3H, major), 1.45 – 1.38 (s, 9H). HRMS (ES+) m/z 379.1200 $[M+Na]^+$ $C_{17}H_{22}N_2O_3FClNa$ requires 379.1195 (Diff = 1.32 ppm).

(*R*)-1-(4-Aminobenzyl)-3-(2-(2-(2-chloro-5-fluorophenyl)pyrrolidin-1-yl)-2-oxoethyl)urea (**4c**)

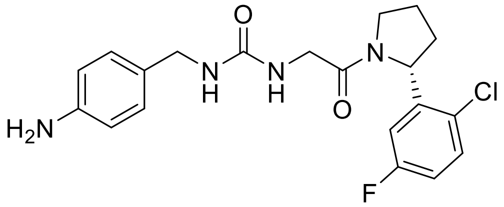

The title compound was obtained following a similar procedure as described for *(R)-1-(4-aminobenzyl)-3-(2-(2-(2,5-dichlorophenyl)pyrrolidin-1-yl)-2-oxoethyl)urea* (**4b**) starting from *tert*-butyl (*R*)-(2-(2-(2-chloro-5-fluorophenyl)pyrrolidin-1-yl)-2-oxoethyl)carbamate (0.12 g, 0.35 mmol, 1.00 eq). The crude product was purified by FCC eluting ethyl acetate to afford the title compound as a white foam (59.4 mg, 42%) with the yield consisting of a mixture of rotamers in ratio 1:1.

^{1}H NMR (400 MHz, $CDCl_3$) δ 7.32 (rotamer 1, dd, *J* = 8.7, 4.9 Hz, 1H), 7.21 (rotamer 2, dd, *J* = 8.7, 5.0 Hz, 1H), 7.05 – 6.95 (m, 2H), 6.94 – 6.87 (rotamer 1, m, 1H), 6.86 – 6.75 (rotamer 2, m, 1H), 5.99 (rotamer 1, dt, *J* = 49.5, 4.6 Hz, 1H), 5.69 (rotamer 2, dt, *J* = 47.4, 5.5 Hz, 1H), 5.32 – 5.22 (rotamer 1, m, 1H), 5.15 (rotamer 2, d, *J* = 8.0 Hz, 1H), 4.26 – 3.97 (m, 4H), 3.83 – 3.46 (m, 3H), 3.39 – 3.15 (m, 1H), 2.36 – 2.15 (m, 1H), 1.99 – 1.57 (m, 3H); ^{13}C NMR (101 MHz, $CDCl_3$) δ 169.7, 169.0, 161.6 (d, *J* = 247.8 Hz), 161.5 (d, *J* = 246.4 Hz), 158.3, 158.2, 145.61, 145.56, 142.0 (d, *J* = 6.5 Hz), 141.4 (d, *J* = 6.5 Hz), 131.7 (d, *J* = 8.2 Hz), 131.2 (d, *J* = 8.3 Hz), 129.5, 129.3, 128.8, 126.7 (d, *J* = 3.2 Hz), 126.6 (d, *J* = 3.0 Hz), 116.0 (d, *J* = 22.9 Hz), 115.2 (d, *J* = 22.9 Hz), 115.14, 115.09, 113.3 (d, *J* = 24.5 Hz), 113.2 (d, *J* = 24.3 Hz), 59.0, 58.5, 47.3, 47.0, 44.0, 43.8, 43.1, 42.8, 33.9, 32.0, 23.4, 21.1. IR ν_{max}/cm^{-1}: 3336 (N-H), 1621 (urea C=O), 1516 (N-H), 1262 (C-F), 813 (C-Cl). ; HRMS (ES+) 427.1301 $[M+Na]^+$ $C_{20}H_{22}N_4O_2{}^{35}ClFNa$ requires 427.1313 (Diff -2.8 ppm); m.p. 76-82°C. Purity HPLC 95.9%, Rt = 6.27 min (UV254 nm).

Synthesis of Compound 4d

***tert*-Butyl (*R*)-(2-(2-(2-chloro-5-methoxyphenyl)pyrrolidin-1-yl)-2-oxoethyl)carbamate**

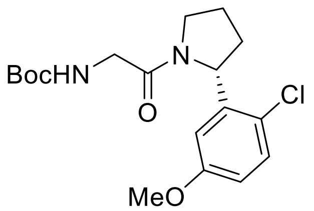

The title compound was obtained following a similar procedure as described for *tert-butyl (R)-(2-(2-(2,5-dichlorophenyl)pyrrolidin-1-yl)-2-oxoethyl)carbamate* starting from (*R*)-2-(2-Chloro-5-methoxyphenyl)pyrrolidine hydrochloride (0.10 g, 0.40 mmol, 1.0 eq). The crude product was purified by FCC (SiO_2; 20:80 EtOAc:Hexane) to afford the title compound as a white foam (0.13 g, 90%) with the yield consisting of a mixture of rotamers in ratio 1.6:1. ^{1}H NMR (400MHz; $CDCl_3$; Me_4Si; ppm) δ 7.32-7.23 (major & minor, m, 2H), 6.75 (major, m, 1H), 6.70 (minor, m, 1H), 6.58 (major, br s, 1H), 6.50 (minor, br s, 1H), 5.45-5.37 (major, m, 1H), 5.20-5.16 (minor, m, 1H), 3.85-3.78 (major & minor, m, 10H), 3.60-3.54 (minor, m, 2H), 3.36-3.30 (major, m, 2H), 2.48-2.25 (minor, m, 2H), 2.08-1.75 (major & minor, m, 6H), 1.44 (minor, s, 9H), 1.41 (major, s, 9H); ^{13}C NMR (100 MHz; $CDCl_3$; Me_4Si) δ 168.4, 167.9, 158.3, 158.2, 152.5, 151.8, 140.7, 140.7, 131.0, 130.6, 126.3, 126.3, 113.4, 113.4, 112.5, 112.5, 79.9, 79.5, 59.1, 58.4, 55.5, 55.4, 47.6, 46.8, 32.0, 31.6, 28.3 (3C), 23.5, 21.2; HRMS (ES+) 391.1393 $[M+Na]^+$ $C_{18}H_{25}N_2O_4{}^{35}ClNa$ requires 391.1401 (Diff -1.9 ppm).

(*R*)-1-(4-Aminobenzyl)-3-(2-(2-(2-chloro-5-methoxyphenyl)pyrrolidin-1-yl)-2-oxoethyl)urea (**4d**)

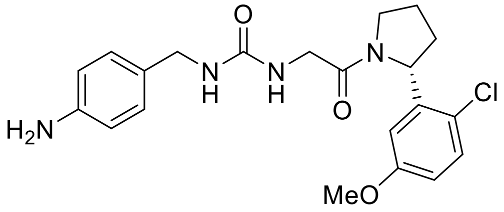

The title compound was obtained following a similar procedure as described for *(R)-1-(4-aminobenzyl)-3-(2-(2-(2,5-dichlorophenyl)pyrrolidin-1-yl)-2-oxoethyl)urea* (**4b**) starting from *tert*-butyl (*R*)-(2-(2-(2-chloro-5-methoxyphenyl)pyrrolidin-1-yl)-2-oxoethyl)carbamate (0.18 g, 0.50 mmol, 1 eq). The crude product was purified by FCC eluting ethyl acetate to afford the title compound as a brown foam (50.5 mg, 25%) with the yield consisting of a mixture of rotamers in ratio 1.1:1. ^{1}H NMR (400 MHz, $CDCl_3$) δ 7.28 (d, J = 9.0 Hz, 1H, rotamer 1), 7.22 (d, J = 8.7 Hz, 1H, rotamer 2), 7.08 – 7.00 (m, 2H), 6.74 (dd, J = 8.7, 3.0 Hz, 1H, rotamer 1), 6.66 (dd, J = 8.7, 3.0 Hz, 1H, rotamer

2), 6.61 (dt, J = 8.4, 1.7 Hz, 2H), 6.50 (d, J = 3.0 Hz, 1H, rotamer 1), 6.48 (d, J = 3.0 Hz, 1H, rotamer 2), 5.70 – 5.62 (m, 1H), 5.40 – 5.04 (m, 2H), 4.27 – 4.03 (m, 4H), 3.76 (s, 3H, rotamer 1), 3.72 (s, 3H, rotamer 2), 3.67 – 3.24 (m, 2H), 2.43 – 2.17 (m, 1H), 2.01 – 1.86 (m, 2H), 1.84 – 1.67 (m, 2H); ^{13}C NMR (101 MHz, $CDCl_3$) δ 169.4, 168.4, 158.8, 158.5, 158.1, 158.0, 145.7, 140.3, 131.1, 130.7, 128.97, 128.95, 123.32, 123.28, 115.30, 115.27, 113.2, 112.9, 112.7, 59.2, 58.6, 55.7, 55.6, 47.6, 47.0, 44.1, 43.3, 42.9, 34.1, 23.5, 21.3. HRMS (ES+) 439.1499 $[M+Na]^+$ $C_{21}H_{25}N_4O_3{}^{35}ClNa$ requires 439.1513 (Diff -3.2 ppm); Purity HPLC 95.7%, Rt = 6.58 min (UV254 nm).

Tert-butyl2-(2-bromophenyl)hydrazinecarboxylate **(5a)**

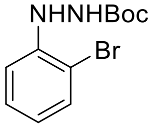

2-Bromophenylhydrazine hydrochloride (1.75 g, 7.83 mmol, 1 eq) was dissolved in THF (7.8 mL). Boc anhydride (1.71 g, 7.83 mmol, 1 eq) and NEt_3 (1.31 ml, 9.40 mmol, 1.2 eq) were added and the reaction mixture was allowed to stir at r.t. overnight. After this time, the reaction mixture was concentrated *in vacuo*. The crude product was purified by flash column chromatography eluting with 100% hexane to 2% EtOAc in hexane to yield an orange solid (2.41 g, 8.43 mmol, Quantitative). ^{1}H NMR (400 MHz; $CDCl_3$; Me_4Si; ppm) δ 7.43 (dd, J = 7.9, 1.3, 1H), 7.22 (ap: t, J = 7.9, 1H), 6.93 (d, J = 7.9, 1H), 6.75 (ap: td, J = 7.9, 1.3, 1H), 6.39 (br s, 1H), 6.20 (s, 1H), 1.47 (s, 9H); ^{13}C NMR (100 MHz; $CDCl_3$; Me_4Si) δ 155.7, 145.1, 132.6, 128.4, 121.5, 113.4, 108.4, 81.6, 28.2; LRMS (CI+) m/z 287 $[M+H]^+$ $C_{11}H_{16}{}^{79}BrN_2O_2$ requires 287, m/z 289 $[M+H]^+$ $C_{11}H_{16}{}^{81}BrN_2O_2$ requires 289.

Tert-butyl2-(2,5-dichlorophenyl)hydrazine-1-carboxylate **(5b)**

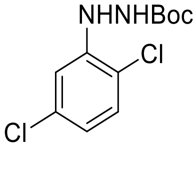

The title compound was obtained following a similar procedure as described for 2-Bromophenylhydrazine hydrochloride starting from (2,5-Dichlorophenyl)hydrazine (1.00 g, 5.67 mmol, 1 eq). The crude product was purified by FCC (SiO_2; 20:80 EtOAc:hexane) to yield a yellow powder (1.50 g, 95%). ^{1}H NMR (400MHz; $CDCl_3$; Me4Si; ppm) δ 7.18 (d, J = 8.4 Hz, 1H), 6.95 (unresolved d, 1H), 6.79 (dd, J = 8.4, 2.4 Hz, 1H), 1.49 (s, 9H). Hydrazine NH peaks not observed.; ^{13}C NMR (100 MHz; $CDCl_3$; Me_4Si) δ 155.7, 145.3, 133.6, 130.2, 120.7, 116.9, 113.9, 82.0, 28.2 (3C); HRMS (ES+) 299.0322 $[M+Na]^+$ $C_{11}H_{14}N_2O_2{}^{35}Cl_2Na$ requires 299.0330 (Diff -2.7 ppm).

Tert-butyl2-(2-bromophenyl)pyrazolidine-1-carboxylate **(7a)**

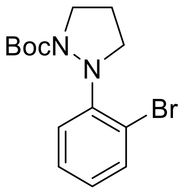

A flame-dried flask was charged with *tert*-butyl2-(2-bromophenyl)hydrazinecarboxylate (2.21 g, 7.73 mmol, 1 eq) and THF (30 mL). The reaction mixture was purged with argon and cooled to -78 °C. *n*-BuLi (1.6 M in hexanes) (9.66 mL, 15.46 mmol, 2 eq) was added dropwise and the reaction mixture was allowed to stir for 15 mins at -78 °C before being allowed to warm to 0 °C and allowed to stir for a further 15 mins. After this time, 1,3-diiodopropane (0.89 mL, 7.73 mmol, 1 eq) was added and the reaction mixture was allowed to stir for 1 hr at -78 °C before warming to r.t. and allowed to stir overnight. After this time, the reaction mixture was quenched with NH_4Cl (60 mL) and extracted with EtOAc (2 x 60 mL). The combined organic layers were dried over $MgSO_4$, filtered and concentrated *in vacuo*. The crude product was purified by flash column chromatography eluting with 5% EtOAc in hexane to yield an orange solid (1.66 g, 5.09 mmol, 66%). ^{1}H NMR (400 MHz; $CDCl_3$; Me_4Si; ppm) δ 7.54 (dd, *J* = 7.9, 1.3, 1H), 7.21 (ap: td, *J* = 7.9, 1.3, 1H), 7.04 (dd, *J* = 7.9, 1.3, 1H), 6.92 (ap: td, *J* = 7.9, 1.3, 1H), 3.76 (t, *J* = 7.4, 2H), 3.52 (t, *J* = 6.6, 2H), 2.05 (ap: quintet, *J* = 7.4, 6.6, 2H), 1.37 (s, 9H); ^{13}C NMR (100 MHz; $CDCl_3$; Me_4Si) δ 147.2, 143.1, 126.8, 120.6, 117.9, 112.6, 108.5, 73.6, 48.5, 39.2, 21.3, 17.3; HRMS (ES+) m/z 349.0524 $[M+Na]^+$ $C_{14}H_{19}{}^{79}BrN_2O_2Na$ requires 349.0528 (Diff = -1.15 ppm), m/z 351.0500 $[M+Na]^+$ $C_{14}H_{19}{}^{81}BrN_2O_2Na$ requires 351.0507 (Diff = -1.99 ppm).

N-*tert*-butyl-2,5-chloro-phenylazapyrrolidine **(7b)**

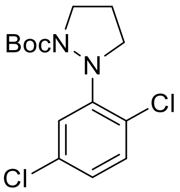

The title compound was obtained following similar procedures as described for *tert*-butyl2-(2-bromophenyl)pyrazolidine-1-carboxylate **(7a)** starting from *tert*-butyl2-(2,5-dichlorophenyl)hydrazine-1-carboxylate (1.00 g, 3.69 mmol, 1.00 eq). The crude product was

purified by FCC (SiO_2; 20:80 EtOAc:hexane) to yield an orange/red solid (0.33 g, 29%). ^{1}H NMR (400MHz; $CDCl_3$; Me_4Si; ppm) δ 7.25 (d, *J* = 7.2 Hz, 1H), 7.06 (d, *J* = 2.4 Hz, 1H), 6.94 (dd, *J* = 8.4, 2.4 Hz, 1H), 3.74 (t, *J* = 7.2 Hz, 2H), 3.54 (t, *J* = 7.2 Hz, 2H), 2.09- 2.02 (m, 2H), 1.41 (s, 9H); ^{13}C NMR (100 MHz; $CDCl_3$; Me_4Si) δ 151.5, 149.8, 131.5, 124.0, 123.2, 119.2, 108.2, 81.0, 54.9, 45.0, 31.5, 28.3 (3C); HRMS (ES+) 339.0636 $[M+Na]^+$ $C_{14}H_{18}N_2O_2{}^{35}Cl_2Na$ requires 339.0643 (Diff -2.1 ppm).

Tert-butyl 2-(2-chloro-5-fluorophenyl)pyrazolidine-1-carboxylate **(7c)**

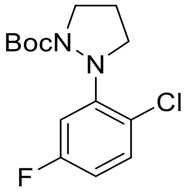

The title compound was obtained following similar procedures as described for *tert*-butyl2-(2-bromophenyl)pyrazolidine-1-carboxylate (**7a**) starting from *tert*-butyl2-(2-chloro-5-fluorophenyl) hydrazine-1-carboxylate (2.5 g, 9.6 mmol). The crude product was purified by FCC (SiO_2; 20:80 EtOAc:Hexane) to afford the title compound as a red solid (1.4 g, 48%). ^{1}H NMR (400 MHz; $CDCl_3$; ppm) δ 7.30-7.26 (m, 1H), 6.82 (dd, *J* = 10.4, 2.8 Hz, 1H), 6.71-6.66 (m, 1H), 3.73 (t, *J* = 6.9 Hz, 2H), 3.55 (t, *J* = 6.9 Hz, 2H), 2.08-2.01 (m, 2H), 1.41 (s, 9H). ^{13}C NMR (101 MHz; $CDCl_3$) δ 161.8 (d, *J* = 246.1 Hz), 154.4, 150.5 (d, *J* = 7.5 Hz), 131.5 (d, *J* = 9.3 Hz), 119.7 (d, *J* = 3.3 Hz), 110.9 (d, *J* = 24.2 Hz), 106.6 (d, *J* = 26.9 Hz), 81.0, 54.9, 46.0, 31.6, 28.7 (3C). HRMS (ES+) 323.0920 $[M+Na]^+$ $C_{14}H_{18}N_2O_2{}^{35}ClFNa$ requires 323.0939 (Diff -2.6 ppm).

1-Benzyl 2-(*tert*-butyl) pyrazolidine-1,2-dicarboxylate

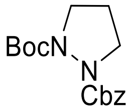

A solution of 1-benzyl 2-(*tert*-butyl) hydrazine-1,2-dicarboxylate (1.03 g, 3.88 mmol, 1 eq) in DMF (7.8 mL) was cooled to 0°C, sodium hydride (0.31 g, 7.76 mmol, 2 eq) was added. The reaction mixture was left to stir for 1 hour then warm to room temperature for 2 hours before addition of 1,3-dibromopropane (0.40 ml, 3.88 mmol, 1 eq). The reaction mixture was allowed to stir at room temperature overnight before addition of EtOAc (50 ml) to the reaction mixture which was then washed with sat. $NaHCO_3$ (3 x 100 mL) followed by distilled water (50 mL) and brine (50 mL). The organic phases were dried over $MgSO_4$ and purified by FCC (SiO_2; 50:50 EtOAc:Hexane) to yield

an orange/brown oil (0.53 g, 45%). ^{1}H NMR (400 MHz; $CDCl_3$; ppm) δ 7.41-7.26 (m, 5H), 5.19 (s, 2H), 3.99-3.85 (unresolved, 2H), 3.35-3.15 (m, 2H), 2.07-1.98 (m, 2H), 1.42 (s, 9H). ^{13}C NMR (101 MHz; $CDCl_3$) δ 156.1, 141.1, 128.9 (2C), 128.5 (2C), 127.7, 125.2, 81.0, 65.2, 46.3 (2C), 31.6, 28.2 (3C). HRMS (ES+) m/z 329.1368 $[M+Na]^+$ $C_{16}H_{22}N_2O_4Na$ requires 329.1477 (Diff -2.8 ppm).

***Tert*-butylpyrazolidine-1-carboxylate**

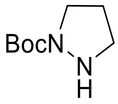

To solution of 1-Benzyl 2-(*tert*-butyl) pyrazolidine-1,2-dicarboxylate (0.73 g, 2.39 mmol, 1 eq) in ethyl acetate (16 ml) was added 10 wt.% Pd/C (0.98 g) under N_2, after which the reaction was allowed to stir at room temperature under an H_2 atmosphere for 16 hrs. After the complete consumption of starting material, the reaction mixture was filtered through celite and the filtrate was concentrated in vacuo to yield the crude product 2.56 (550 mg, 98%) as a colourless oil. No further purification is needed R_f = 0.30, 25% ethyl acetate in hexane. ^{1}H NMR (400 MHz; $CDCl_3$; ppm) 3.92 (s, 1H), 3.46 (t, J = 7.3 Hz, 2H), 3.06 (t, J = 7.3 Hz, 2H), 2.10-1.98 (m, 2H), 1.49 (s, 9H). ^{13}C NMR (101 MHz, $CDCl_3$) δ 156.1, 81.0, 46.3 (2C), 31.6, 28.2 (3C); LRMS (CI+) m/z 71.1 $[M-Boc]^+$ requires $C_3H_7N_2$ 71.1.

2-Bromo-1-chloro-4-methoxybenzene **(6)**

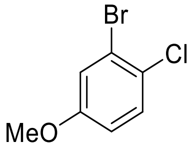

To a solution of 3-bromo-4-chlorophenol (0.40 g, 1.93 mmol, 1 eq) and potassium carbonate (0.43 g, 3.09 mmol, 1.6 eq) in acetone (0.2 M, 7.7 mL), iodomethane (0.18 mL, 2.89 mmol, 1.5 eq) was added dropwise and reaction mixture was allowed to stir at room temperature overnight. EtOAc (10 ml) was added to the reaction mixture and washed with sat. aq. $NaHCO_3$ (3 x 10 mL), distilled water (10 mL) and brine (10 ml) then dried over $MgSO_4$. The crude product was purified by FCC (SiO_2; 20:80 EtOAc:Hexane) to afford the title compound as a clear yellow oil (0.23 g, 53%). ^{1}H NMR (400 MHz; $CDCl_3$; ppm) δ 7.33 (d, J = 9.0 Hz, 1H) 7.16 (d, J = 3.0 Hz, 1H), 6.80 (dd, J = 9.0, 3.0 Hz, 1H), 3.79 (s, 3H). ^{13}C NMR (101 MHz; $CDCl_3$) δ 158.6, 130.5, 122.9, 122.6, 118.8, 114.8, 55.8. LRMS (CI+) m/z 220.9 $[M+H]^+$ $C_7H_6{}^{79}BrO^{35}ClH$ requires 220.9.

Tert-butyl2-(2-chloro-5-methoxyphenyl)pyrazolidine-1-carboxylate **(7d)**

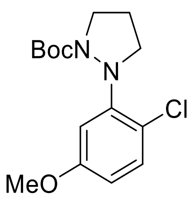

To a solution of *tert*-butylpyrazolidine-1-carboxylate (0.22 g, 1.24 mmol, 1.1 eq) in toluene (10 mL), was added Cs_2CO_3 (1.29 g, 3.39 mmol, 3.5 eq), *t*BuXPhos (48 mg, 0.11 mmol, 0.1 eq) and 2-Bromo-1-chloro-4-methoxybenzene (0.25 g, 1.13 mmol, 1 eq). The reaction mixture was evacuated and flushed with N_2 twice before $Pd_2(dba)_3$ (52 mg, 0.056 mmol, 0.05 eq) was added and the reaction mixture was allowed to reflux overnight. After this time, the reaction mixture was cooled to room temperature and extracted with EtOAc (x 2), washed with brine, dried over $MgSO_4$, filtered and concentrated *in vacuo* to yield the crude product. The crude product was purified was purified by FCC (SiO_2; 20:80 EtOAc:Hexane) to afford the title compound as a yellow oil (0.10 g, 28%). ^{1}H NMR (400 MHz; $CDCl_3$; ppm) δ 7.16 (d, *J* = 8.2 Hz, 1H), 6.56 (d, *J* = 2.8 Hz, 1H), 6.45 (dd, *J* = 8.2, 2.8 Hz, 1H), 3.68 (s, 3H), 3.66 (t, *J* = 7.0 Hz, 2H), 3.47 (t, *J* = 7.0 Hz, 2H), 2.01-1.93 (m, 2H), 1.34 (s, 9H). ^{13}C NMR (101 MHz; $CDCl_3$) δ 158.8, 149.6, 131.1, 120.0, 116.6, 109.1, 105.5, 80.8, 55.5, 55.0, 46.0, 30.9, 28.3 (3C). LRMS (ES+) m/z 335.1 $[M+Na]^+$ $C_{15}H_{21}O_3N_2ClH$ requires 335.1.

Tert-butyl(2-(2-(2-bromophenyl)pyrazolidin-1-yl)-2-oxoethyl)carbamate (**8a**)

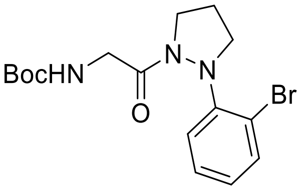

Tert-butyl2-(2-bromophenyl)pyrazolidine-1-carboxylate (1.56 g, 4.78 mmol, 1 eq) was dissolved in a mixture of DCM:TFA (5:1, 48 mL, ~ 10mL/mmol) and the reaction mixture was allowed to stir at room temperature until TLC showed disappearance of starting material. The reaction mixture was concentrated *in vacuo* to afford the free amine as a trifluoroacetate salt and was used directly without further purification.

To a solution of the trifluoroacetate salt of the amine (105 mg, 0.31 mmol) in anhydrous DMF (5 mL), was added boc-glycine (65 mg, 0.92 mmol, 1.2 eq), DIPEA (0.13 mL, 0.77 mmol, 2.5 eq) and HATU (0.15 g, 0.40 mmol, 1.3 eq). The reaction mixture was allowed to stir at room temperature overnight under N_2 (followed by TLC). Upon completion, the solution was diluted with EtOAc and washed with sat. aq. $NaHCO_3$ (x 3), water and brine. The organic layer was dried over $MgSO_4$ and concentrated *in vacuo*. The crude product was purified by flash column chromatography eluting with

35% EtOAc in hexane to 45% EtOAc in hexane to yield a yellow oil (125 mg, 0.33 mmol, Quantitative). ^{1}H NMR (400 MHz; $CDCl_3$; Me_4Si; ppm) δ 7.57 (d, *J* = 7.6, 1H), 7.22 (ap: t, *J* = 7.6, 1H), 7.00 – 6.95 (m, 2H), 5.22 (s, 1H), 4.36 (br s,1H), 3.87 (br s, 2H), 3.48 (br s, 3H), 2.07 (br s, 2H), 1.42 (m, 9H); ^{13}C NMR (100 MHz; $CDCl_3$; Me_4Si) δ 170.7, 155.8, 148.3, 134.3, 128.1, 126.0, 119.2, 115.8, 79.4, 55.9, 45.4, 42.6, 28.4, 23.2; HRMS (ES+) m/z 406.0739 $[M+Na]^+$ $C_{16}H_{22}{}^{79}BrN_3O_3Na$ requires 406.0742 (Diff = -0.74 ppm), m/z 408.0726 $[M+Na]^+$ $C_{16}H_{22}{}^{81}BrN_3O_3Na$ requires 408.0722 (Diff = 0.98 ppm).

Tert-butyl(2-(2-(2,5-dichlorophenyl)pyrazolidin-1-yl)-2-oxoethyl)carbamate **(8b)**

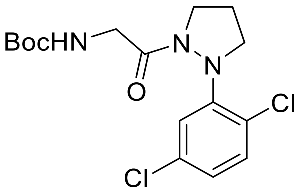

The title compound was obtained following similar procedures as described for *tert*-butyl(2-(2-(2-bromophenyl)pyrazolidin-1-yl)-2-oxoethyl)carbamate **(8a)** starting from *tert*-butyl2-(2,5-dichlorophenyl)pyrazolidine-1-carboxylate (0.33 g, 1.05 mmol, 1 eq). The crude product was purified by FCC (SiO_2; 20:80 EtOAc:hexane) to yield an orange/brown oil (0.24 g, 59%).

^{1}H NMR (400MHz; $CDCl_3$; Me_4Si; ppm) δ 7.30 (d, *J* = 8.4 Hz, 1H), 7.02 (dd, *J* = 8.4, 2.4 Hz, 1H), 6.98 (d, *J* = 2.0 Hz, 1H), 4.03 (s, 2H), 3.8-3.3 (m, 4H), 2.09 (m, 2H), 1.42 (s, 9H). Carbamate NH not observed.; ^{13}C NMR (100 MHz; $CDCl_3$; Me_4Si) δ 171.1, 155.8, 148.2, 133.3, 132.0, 125.4, 123.8, 119.0, 79.6, 55.4, 45.0, 42.5, 28.3 (3C) – one carbon environment missing; HRMS (ES+) 396.0849 $[M+Na]^+$ $C_{16}H_{21}N_3O_3{}^{35}Cl_2Na$ requires 396.0858 (Diff -2.2 ppm).

Tert-butyl(2-(2-(2-chloro-5-fluorophenyl)pyrazolidin-1-yl)-2-oxoethyl)carbamate **(8c)**

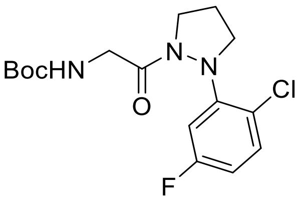

The title compound was obtained following a similar procedure as described for *tert*-butyl(2-(2-(2-bromophenyl)pyrazolidin-1-yl)-2-oxoethyl)carbamate (**8a**) starting from *tert*-butyl2-(2-chloro-5-fluorophenyl)pyrazolidine-1-carboxylate (0.094 g, 0.31 mmol, 1.00 eq). The crude product was purified by FCC (SiO_2; 20:50 EtOAc:Hexane) to afford the title compound as an orange solid (64.3

mg, 57%). ^{1}H NMR (400 MHz; $CDCl_3$; ppm) δ 7.33 (dd, *J* = 8.4, 6.0 Hz, 1H), 6.80-6.74 (m, 2H), 4.02 (s, 2H), 4.0-3.4 (unresolved m, 4H), 2.15-2.00 (unresolved m, 2H), 1.42 (s, 9H). Carbamate NH not observed.; ^{13}C NMR (101 MHz; $CDCl_3$) δ 171.1, 161.8 (d, *J* = 248.8 Hz), 160.5, 155.8, 148.7 (d, *J* = 8.2 Hz), 132.1 (d, *J* = 9.2 Hz), 118.9 (d, *J* = 1.5 Hz), 112.3 (d, *J* = 23.2 Hz), 106.4 (d, *J* = 27.1 Hz), 79.5, 55.3, 45.0, 31.6, 28.3 (3C). HRMS (ES+) 380.1150 $[M+Na]^+$ $C_{16}H_{21}N_3O_3{}^{35}ClFNa$ requires 380.1153 (Diff -0.8 ppm).

Tert-butyl2-(2-chloro-5-methoxyphenyl)pyrazolidine-1-carboxylate **(8d)**

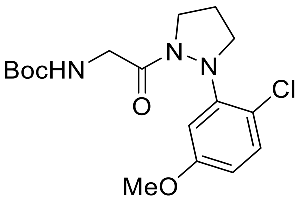

The title compound was obtained following similar procedures as described for *tert*-butyl(2-(2-(2-bromophenyl)pyrazolidin-1-yl)-2-oxoethyl)carbamate (**8a**) starting from *tert*-butyl2-(2-chloro-5-methoxyphenyl)pyrazolidine-1-carboxylate (0.24 g, 0.76 mmol, 1 eq). The crude product was purified by FCC (SiO_2; 20:80 EtOAc:Hexane) to afford the title compound as an orange/brown oil (0.24 g, 84%). ^{1}H NMR (400 MHz; $CDCl_3$; ppm) δ 7.26 (d, *J* = 8.7 Hz, 1H), 6.57 (dd, *J* = 8.7, 2.8 Hz, 1H), 6.52 (dd, *J* = 2.8 Hz, 1H), 5.22 (br s, 1H), 4.05-3.78 (m, 2H), 3.75 (s, 3H), 3.68-3.40 (m, 4H), 2.06 (s, 2H), 1.41 (s, 9H); ^{13}C NMR (101 MHz; $CDCl_3$) δ 170.8, 159.0, 155.8, 147.9, 131.6, 116.9, 109.8, 105.5, 79.5, 60.4, 55.6, 55.4, 45.0, 42.5, 28.4 (3C). HRMS (ES+) m/z 392.1345 $[M+Na]^+$ $C_{17}H_{24}N_3O_4Na$ requires 392.1353 (Diff -2.1 ppm).

Tert-butyl(*S*)-(2-(2-(2-bromophenyl)pyrazolidin-1-yl)-2-oxo-1-phenylethyl)carbamate **(9a)**

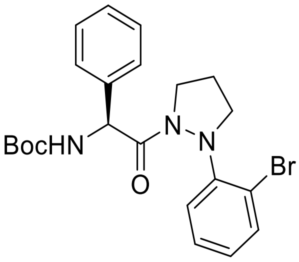

Tert-butyl2-(2-bromophenyl)pyrazolidine-1-carboxylate (0.58 g, 1.77 mmol, 1 eq) was dissolved in a mixture of DCM:TFA (5:1, 18 mL, ~ 10mL/mmol) and the reaction mixture was allowed to stir at room temperature until TLC showed disappearance of starting material. The reaction mixture was

concentrated *in vacuo* to afford the free amine as a trifluoroacetate salt and was used directly without further purification.

To a solution of the trifluoroacetate salt of 1-(2-bromophenyl)pyrazolidine (0.60 mg, 1.76 mmol) in anhydrous DMF (20 mL), was added Boc-phenylglycine-OH (0.53 mg, 2.11 mmol, 1.2 eq), DIPEA (0.77 mL, 4.40 mmol, 2.5 eq) and HATU (0.87 g, 2.29 mmol, 1.3 eq). The reaction mixture was allowed to stir at room temperature overnight under N_2 (followed by TLC). Upon completion, the solution was diluted with EtOAc and washed with sat. aq. $NaHCO_3$ (x 3), water and brine. The organic layer was dried over $MgSO_4$ and concentrated *in vacuo*. The crude product was purified by FCC (SiO_2; 20:80 to 50:50 EtOAc:Hexane) to afford the title compound as a yellow oil (0.65 g, 80%). With the yield consisting of a mixture of rotamers in ratio 2/1. ^{1}H NMR (400 MHz; $CDCl_3$; ppm) δ 7.56-7.35 (m, 1H), 7.35-7.07 (m, 3H), 7.07-6.69 (m, 4H), 6.69-6.48 (m, 1H), 6.12 (major, s, 1H), 5.81 (major, s, 1H), 5.74 (minor, s, 1H), 5.33 (minor, s, 1H), 4.05-3.98 (minor, m, 2H), 3.86-3.74 (minor, m, 2H), 3.56-3.44 (major, m, 2H), 3.36-3.26 (major, m, 2H), 1.99-1.90 (minor, m, 2H), 1.84-70 (major, m, 2H), 1.30 (major, s, 9H), 1.25 (minor, s, 9H). ^{13}C NMR (101 MHz; $CDCl_3$) δ 172.6, 171.8, 154.7, 154.5, 148.2, 147.5, 137.8, 137.0, 134.5, 133.4, 128.3 (2C), 128.1 (2C), 127.7 (2C), 127.2 (2C), 125.9, 124.9, 119.1, 118.9, 115.0, 114.3, 79.5, 79.3, 56.2, 56.1, 55.1, 54.9, 45.7, 45.4, 36.9, 35.9, 28.4 (3C), 28.0 (3C). HRMS (ES+) 482.1063 $[M+Na]^+$ $C_{22}H_{26}N_3O_3{}^{79}BrNa$ requires 482.1050 (Diff -2.67 ppm).

Tert-butyl(S)-(2-(2-(2-chloro-5-methoxyphenyl) pyrazolidin-1-yl)-2-oxo-1-phenylethyl)Carbamate **(9b)**

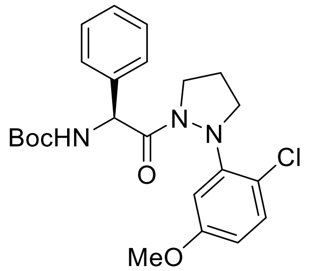

The title compound was obtained following a similar procedure as described for *tert*-butyl(*S*)-(2-(2-(2-bromophenyl)pyrazolidin-1-yl)-2-oxo-1-phenylethyl)carbamate (**9a**) starting from *tert*-butyl2-(2-chloro-5-methoxyphenyl)pyrazolidine-1-carboxylate (450 mg, 1.44 mmol). The crude product was purified by FCC (SiO_2; 50:50 EtOAc:Hexane) to give the title compound as an orange oil (0.38 g, 60%). The product was present as a mixture of rotamers in ratio 3/1. ^{1}H NMR (400 MHz; $CDCl_3$) δ 7.46-7.45 (m, 1H), 7.42-7.40 (m, 1H), 7.36-7.34 (m, 1H), 7.32-7.28 (m, 1H), 7.13-7.06 (m, 2H), 6.92-6.88 (m, 2H), 6.59 (major, s, 1H), 6.30 (major, s, 1H), 5.84 (minor, s, 1H), 5.66 (minor, s, 1H), 4.07-

4.05 (minor, m, 2H), 3.94-3.83 (minor, s, 2H), 3.76 (major, s, 2H), 3.62-3.52 (major, m, 2H), 3.44 (s, 3H), 2.07 (minor, s, 2H), 1.87 (major, br s, 2H), 1.44 (minor, s, 9H), 1.42 (major, s, 9H). ^{13}C NMR (101 MHz; $CDCl_3$) δ 172.6, 172.0, 170.0, 159.0, 158.2, 155.0, 154.7, 154.5, 147.8, 147.1, 137.9, 137.7, 137.1, 131.63, 130.6, 128.9, 128.2, 128.1, 127.9, 127.7, 127.7, 127.1, 116.1, 115.8, 110.3, 110.0, 105.3, 104.6, 79.5, 79.3, 56.5, 56.2, 55.5, 55.1, 54.3, 45.4, 45.0, 35.9, 28.3 (3C), 23.6, 23.1 (3C), 22.6. HRMS (ES+) m/z $C_{23}H_{28}ClN_3O_4Na$ $[M+ Na]^+$ requires 468.1661, found 468.1670 (Diff = -1.92 ppm).

1-(4-Aminobenzyl)-3-(2-(2-(2-bromophenyl)pyrazolidin-1-yl)-2-oxoethyl)urea **(12a)**

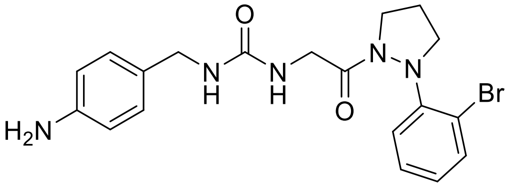

Tert-butyl(2-(2-(2-bromophenyl)pyrazolidin-1-yl)-2-oxoethyl)carbamate (**8a**) (0.25 g, 0.65 mmol, 1 eq) was dissolved in a mixture of DCM:TFA (5:1, 8 mL) and the reaction mixture was allowed to stir at room temperature until TLC showed disappearance of starting material. The reaction mixture was concentrated *in vacuo* to afford the free amine as a trifluoroacetate salt and was used directly without further purification.

To a solution of the trifluoroacetate salt (0.25 g, 0.63 mmol, 1 eq) in acetonitrile (8 mL), was added *N*-(4-Aminobenzyl)-1*H*-imidazole-1-carboxamide (0.15 g, 0.69 mmol, 1.1 eq), triethylamine (0.19 mL, 1.38 mmol, 2.2 eq) and DMAP (7.7 mg, 0.06 mmol, 0.1 eq) and was allowed to stir at room temperature overnight under N_2 (followed by TLC). Upon completion, the solution was diluted with DCM and washed with distilled water. The organic phase was dried over $MgSO_4$ and concentrated *in vacuo*. The crude product was purified by flash column chromatography eluting with 100% EtOAc to 2% MeOH in EtOAc to yield a yellow foam (207 mg, 0.48 mmol, 76%). M.p: 132 – 135 °C; IR ν_{max} (cm^{-1}) 753 (C-Br), 1517 (N-H), 1542 (N-H), 1621 (amide C=O), 3388 (N-H); ^{1}H NMR (400 MHz; $CDCl_3$; Me_4Si; ppm) δ 7.56 (dd, *J* = 8.0, 1.3, 1H), 7.19 (ap: td, *J* = 8.0, 1.3, 1H), 7.09 (s, 2H), 7.06 (d, *J* = 8.4, 2H), 6.97 (ap: td, *J* = 8.0, 1.3, 1H), 6.85 (dd, *J* = 8.0, 1.3, 1H), 6.61 (d, *J* = 8.4, 2H) 5.57 (t, *J* = 4.4, 1H), 5.36 (t, *J* = 5.1, 1H), 5.25 (br s, 2H), 4.23 (d, *J* = 5.1, 2H), 3.64 (br s, 2H), 3.42 (br s, 2H), 1.98 (m, 2H); ^{13}C NMR (100 MHz; $CDCl_3$; Me_4Si) δ 171.3, 158.1, 148.1, 145.6, 135.1, 134.3, 128.9, 128.1, 126.1, 121.9, 119.1, 115.1, 55.8, 45.3, 44.1, 42.4, 23.0; HRMS (ES+) m/z 454.0851 $[M+Na]^+$ $C_{19}H_{22}{}^{79}BrN_5O_2Na$ requires 454.0855 (Diff = -0.88 ppm), m/z 456.0837 $[M+Na]^+$

$C_{19}H_{22}{}^{81}BrN_5O_2Na$ requires 456.0834 (Diff = 0.66 ppm). Anal. Cal. (mass %) for $C_{19}H_{22}BrN_5O_2$: C, 59.73; H, 5.13; N, 16.20. Found: C, 59.55; H, 5.03; N, 16.01.

1-(4-Aminobenzyl)-3-(2-(2-(2,5-dichlorophenyl)pyrazolidin-1-yl)-2-oxoethyl)urea **(12b)**

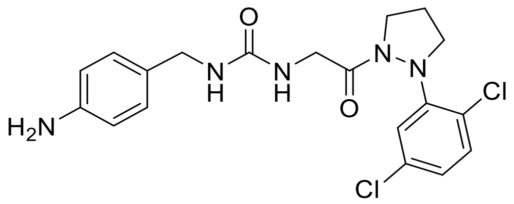

The title compound was obtained following a similar procedure as described for 1-(4-Aminobenzyl)-3-(2-(2-(2-bromophenyl)pyrazolidin-1-yl)-2-oxoethyl)urea (**12a**) starting from *tert*-butyl2-(2,5-dichlorophenyl)pyrazolidine-1-carboxylate (**8b**) (0.22 g, 0.59 mmol, 1.00 eq) The crude product was purified by FCC (SiO_2; EtOAc) to yield a pale white powder (92.70 mg, 37%). M.p. 182.0-188.0°C; ^1H NMR (400 MHz, $CDCl_3$) δ 7.29 (d, *J* = 8.5 Hz, 1H), 7.07 (d, *J* = 8.3 Hz, 2H), 7.01 (dd, *J* = 8.5, 2.4 Hz, 1H), 6.85 (d, *J* = 2.4 Hz, 1H), 6.61 (d, *J* = 8.3 Hz, 2H), 5.69 (t, *J* = 4.6 Hz, 1H), 5.46 (t, *J* = 5.4 Hz, 1H), 4.67 – 4.34 (m, 1H), 4.22 (d, *J* = 5.3 Hz, 2H), 3.87 – 3.03 (m, 7H), 2.01 – 1.82 (m, 2H); ^{13}C NMR (101 MHz, $CDCl_3$) δ 172.1, 158.1, 148.2, 145.8, 133.3, 132.2, 129.3, 129.2, 125.5, 124.1, 119.0, 115.2, 55.4, 45.0, 44.3, 42.4, 23.4. IR *v*max/cm-1: 3354 (N-H), 1603 (urea C=O), 809 (C-Cl); HRMS (ES+) 444.0965 [M+Na]+ $C_{19}H_{21}N_5O_2{}^{35}Cl_2Na$ requires 444.0970 (Diff -1.1 ppm). Anal. Cal. (mass %) for $C_{19}H_{21}Cl_2N_5O_2$: C, 54.04; H, 5.01; N, 16.58. Found C, 53.88; H, 4.99; N, 16.32.

1-(4-Aminobenzyl)-3-(2-(2-(2-chloro-5-fluorophenyl)pyrazolidin-1-yl)-2-oxoethyl)urea **(12c)**

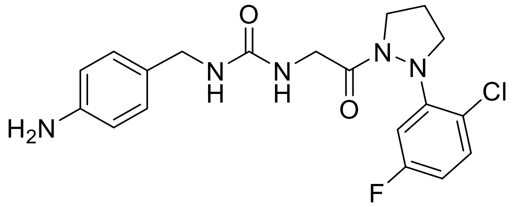

The title compound was obtained following a similar procedure as described for 1-(4-Aminobenzyl)-3-(2-(2-(2-bromophenyl)pyrazolidin-1-yl)-2-oxoethyl)urea (**12a**) starting from *tert*-butyl(2-(2-(2-chloro-5-fluorophenyl)pyrazolidin-1-yl)-2-oxoethyl)carbamate (**8c**) (0.11 g, 0.31 mmol). The crude product was purified by FCC (SiO_2; EtOAc) to afford the title compound as an orange solid (25.4 mg, 20%). M.p. 123.5-125.0°C; ^1H NMR (400 MHz, MeOD) δ 7.45 (dd, *J* = 8.2, 5.6 Hz, 1H), 7.02 (d, *J* = 8.3 Hz, 2H), 6.94 – 6.84 (m, 2H), 6.67 (d, *J* = 8.3 Hz, 2H), 4.49 – 4.15 (m, 1H), 4.14 (s, 2H), 4.00 – 3.43 (m, 5H), 2.19 – 1.95 (m, 2H). ^{13}C NMR (101 MHz, DMSO) δ 171.3, 161.2 (d, *J* = 245.1 Hz), 157.8, 149.3 (d, *J* = 8.0 Hz), 147.3, 132.0 (d, *J* = 8.9 Hz), 128.0, 127.4, 119.7 (d, *J* = 3.2 Hz),

113.7, 112.1 (d, J = 23.1 Hz), 107.1 (d, J = 26.1 Hz), 55.0, 44.5, 42.7, 41.4, 23.1. HRMS (ES+) 428.1255 $[M+Na]^+$ $C_{19}H_{21}N_5O_2{}^{35}ClFNa$ requires 428.1266 (Diff -2.5 ppm). Purity HPLC 95.7%, Rt = 6.48 min (UV254 nm).

1-(4-Aminobenzyl)-3-(2-(2-(2-chloro-5-methoxyphenyl)pyrazolidin-1-yl)-2- oxoethyl)urea **(12d)**

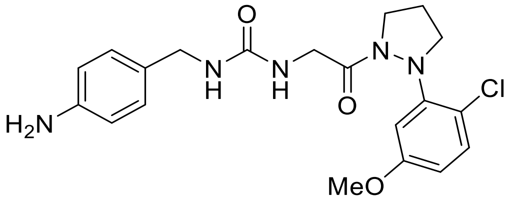

The title compound was obtained following a similar procedure as described for 1-(4-Aminobenzyl)-3-(2-(2-(2-bromophenyl)pyrazolidin-1-yl)-2-oxoethyl)urea (**12a**) starting from *tert*-butyl2-(2-chloro-5-methoxyphenyl)pyrazolidine-1- carboxylate (**8d**) (0.11 g, 0.30 mmol, 1.00 eq). The crude product was purified by FCC (SiO_2; EtOAc) to yield a pale white powder (0.12 g, 96%). M.p. 194.0–196.0°C; ^{1}H NMR (400 MHz, $CDCl_3$) δ 7.30 – 7.23 (m, 1H), 7.11 – 7.04 (m, 2H), 6.66 – 6.59 (m, 2H), 6.57 (dd, J = 8.8, 2.9 Hz, 1H), 6.49 (d, J = 2.9 Hz, 1H), 5.26 – 5.14 (m, 1H), 4.82 (t, J = 5.5 Hz, 1H), 4.54 – 4.24 (m, 1H), 4.23 (d, J = 5.5 Hz, 2H), 3.86 – 3.22 (m, 10H), 2.04 – 1.96 (m, 2H); ^{13}C NMR (101 MHz, DMSO) δ 171.0, 158.7, 157.7, 148.3, 147.3, 131.2, 128.0, 127.4, 115.7, 113.7, 110.2, 105.6, 55.5, 55.1, 44.6, 42.7, 41.4, 23.0. IR *v*max/cm-1: 3350 (N-H), 1630 (urea C=O), 1568 (N-H), 824 (C-Cl); HRMS (ES+) m/z 440.1457 $[M+Na]^+$ $C_{20}H_{24}ClN_5O_3Na$ requires 440.1465 (Diff -1.9 ppm); Purity HPLC 95.1%, Rt = 6.58 min (UV254 nm).

(*S***)**-1-(4-Aminobenzyl)-3-(2-(2-(2-bromophenyl)pyrazolidin-1-yl)-2-oxo-1-phenylethyl)urea **(13)**

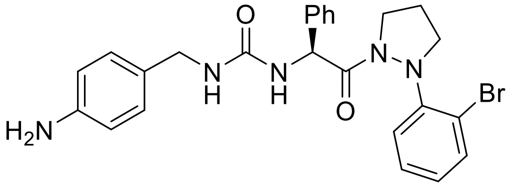

Tert-butyl(*S*)-(2-(2-(2-bromophenyl)pyrazolidin-1-yl)-2-oxo-1-phenylethyl)carbamate (**9a**) (0.30 g, 0.65 mmol, 1.00 eq) was dissolved in a mixture of DCM:TFA (5:1, 8 mL) and the reaction mixture was allowed to stir at room temperature until TLC showed disappearance of starting material. The reaction mixture was concentrated *in vacuo* to afford the free amine as a trifluoroacetate salt and was used directly without further purification.

To a solution of the trifluoroacetate salt (0.31 g, 0.65 mmol, 1 eq) in acetonitrile (8 mL), was added *N*-(4-Aminobenzyl)-1*H*-imidazole-1-carboxamide (0.15 g, 0.70 mmol, 1.1 eq), triethylamine (0.20 mL, 1.41 mmol, 2.2 eq) and DMAP (7.9 mg, 0.06 mmol, 0.1 eq) and was allowed to stir at room

temperature overnight under N_2 (followed by TLC). Upon completion, the solution was diluted with DCM and washed with distilled water. The organic phase was dried over $MgSO_4$ and concentrated *in vacuo*. The crude product was purified by FCC (SiO_2; EtOAc) to afford the title compound as a yellow solid (0.17 g, 50%). M.p. 103-104 °C; IR ν_{max} (cm^{-1}) 3336 (N-H), 1619 (urea C=O), 1515 (N-H), 696 (C-Br); ^{1}H NMR (400 MHz, DMSO) δ 7.71 – 7.01 (m, 6H), 6.97 – 6.72 (m, 6H), 6.63 – 6.39 (m, 4H), 6.32 (t, *J* = 5.6 Hz, 1H), 6.04 – 5.39 (m, 1H), 4.90 (s, 2H), 4.00 – 3.56 (m, 4H), 1.96 – 1.67 (m, 2H). ^{13}C NMR (101 MHz, MeOD) δ 175.1, 159.9, 149.2, 147.7, 137.9, 135.4, 134.5, 130.2, 129.4, 129.2, 128.9, 128.6, 128.6, 126.4, 120.8, 116.6, 116.3, 57.2, 46.8, 44.5, 42.2, 23.6. HRMS (ES+) 530.1156 $[M+Na]^+$ $C_{25}H_{26}N_5O_2{}^{79}BrNa$ requires 530.1168 (Diff -2.2 ppm), m/z 532.1151 $[M+Na]^+$ $C_{25}H_{26}N_5O_2{}^{81}BrNa$ requires 532.1147 (Diff 0.70 ppm). $[\alpha]^{20}_D$ = 10.0 (*c* 1.0, CH_3OH); Purity HPLC 95.6%, Rt = 7.62 min (UV254 nm). Chiral HPLC >95% e.e., Rt = 8.53 min.

Tert-butyl(4-aminobenzyl)carbamate (**14**)

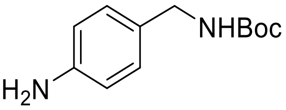

To a solution of 4-(aminomethyl)aniline (4.70 g, 38.50 mmol, 1 eq) in THF (1M) followed by addition of Boc_2O (9.20 g, 42.40 mmol, 1.1 eq) and dropwise addition of NEt_3 (16 mL, 115.50 mmol, 3 eq). The reaction mixture was allowed to stir overnight. EtOAc (100 mL) was added to the reaction mixture and washed with 1M HCl (50 mL), distilled water (100 mL), brine (100 mL). The organic phases were then combined, dried over $MgSO_4$ and concentrated *in vacuo* to afford a crude product. The crude product was purified by FCC (SiO_2; 20:80 EtOAc:Hexane) to afford the title compound as a yellow solid (4.30 g, 50%). ^1H NMR (500 MHz; $CDCl_3$; ppm) δ 7.10 (d, J = 7.8 Hz, 2H), 6.63 (d, J = 7.8 Hz, 2H), 4.89 (br s, 1H), 4.17 (s, 2H), 3.70 (br s, 2H), 1.45 (s, 9H). ^{13}C NMR (126 MHz; $CDCl_3$) δ 155.9, 145.7, 128.9, 128.8 (2C), 115.2 (2C), 80.5, 44.4, 28.4 (3C). HRMS (ES+) m/z 245.1263 $[M+Na]^+$ $C_{12}H_{18}N_2O_2Na$ requires 245.126 (Diff -0.98 ppm).

Tert-butyl(((2*S*,3*S*,6*S*)-3-hydroxy-1,3,4,6-tetrahydro-2H-2,6-methanobenzo[c][1,5]oxazocin-8-yl)methyl)carbamate and *tert*-butyl(((2*R*,3*S*,6*R*)-3-hydroxy-1,3,4,6-tetrahydro-2H-2,6-methanobenzo[c][1,5]oxazocin-8-yl)methyl)carbamate (**15**)

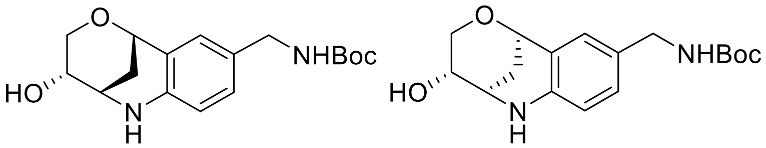

A mixture of 2-deoxy-*D*-ribose (2 g, 14.90 mmol, 1 eq), *tert*-butyl(4-aminobenzyl)carbamate (**14**) (4.97 g, 22.4 mmol, 1.5 eq) and montmorillonite (14.5 g) in MeCN (150 mL) was stirred at room temperature for 5 days. The reaction mixture was filtered through celite and concentrated *in vacuo*. The crude product was purified by FCC (SiO_2; 20:80 to 40:60 Acetone:Hexane) to afford the title diastereoisomers.

Second eluting: *tert*-butyl(((2*R*,3*S*,6*R*)-3-hydroxy-1,3,4,6-tetrahydro-2H-2,6-methanobenzo[c][1,5]oxazocin-8-yl)methyl)carbamate (**15**) as a yellow solid (0.96 g, 38%). ^1H NMR (500 MHz; $CDCl_3$; ppm) δ 7.04 (d, J = 8.0 Hz, 1H), 7.00 (br s, 1H), 6.51 (d, J = 8.0 Hz, 1H), 5.00 (br s, 1H), 4.69 (br s, 1H), 4.65 (s, 1H), 4.19-4.12 (m, 2H), 3.63-3.50 (m, 2H), 3.48-3.39 (m, 3H), 2.57 (d, J = 12.5 Hz, 1H), 1.44 (s, 9H). Glycan OH not observed. ^{13}C NMR (126 MHz; $CDCl_3$) δ 156.0, 144.2, 130.0, 129.4, 126.7, 118.3, 113.1, 79.4, 77.4, 69.8, 68.8, 63.4, 47.7, 44.2, 28.5 (3C). HRMS (ES+) m/z 343.1630 $[M+Na]^+$ $C_{17}H_{24}N_2O_4Na$ requires 343.1628 (Diff -0.46 ppm). $[\alpha]^{20}_D$ = 5 (c 1.0, CH_3OH). R_f = 0.34 (50:50 Acetone:Hexane).

Spectroscopic data are in agreement with the literature.[2]

Tert-butyl(((2*R*,3*S*,6*R*)-3-((*tert*-butyldimethylsilyl)oxy)-1,3,4,6-tetrahydro-2H-2,6-methanobenzo[c][1,5]oxazocin-8-yl)methyl)carbamate (**16**)

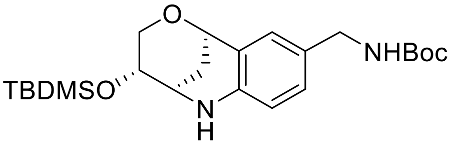

A solution of *tert*-butyl(((2*R*,3*S*,6*R*)-3-hydroxy-1,3,4,6-tetrahydro-2H-2,6-methanobenzo[c][1,5]oxazocin-8-yl)methyl)carbamate (**15**) (0.95 g, 2.98 mmol, 1 eq) and pyridine (0.72 ml, 8.90 mmol, 3 eq) in DCM (20 mL) was cooled to 0°C followed by the dropwise addition of *tert*-butyldimethylsilyl trifluoromethanesulfonate (1.02 mL, 4.45 mmol, 1.5 eq). The reaction mixture was stirred at room temperature until TLC showed disappearance of starting material. The reaction mixture was diluted with DCM (20 mL) and washed with 1M HCl (10 mL). The organic and aqueous phases were separated, and the aqueous phase was neutralised and extracted with DCM (2 x 20 mL). The organic phases were then combined, dried over $MgSO_4$ and concentrated *in vacuo*. The crude product was purified by FCC (SiO_2; 30:70 EtOAc:Hexane) to afford the title compound as a yellow solid (0.72 g, 56%). ^{1}H NMR (500 MHz; $CDCl_3$; ppm) δ 7.07-6.98 (m, 2H), 6.48 (d, *J* = 8.0 Hz, 1H), 5.07 (br s, 1H), 4.89 (br s, 1H), 4.65 (s, 1H), 4.60 (s, 1H), 4.19-4.13 (m, 2H), 3.50-3.43 (m, 3H), 3.36-3.33 (m, 1H), 2.65 (d, *J* = 12.5 Hz, 1H), 1.45 (s, 9H), 0.93 (s, 9H), 0.09 (s, 3H), 0.08 (s, 3H). ^{13}C NMR (126 MHz; $CDCl_3$) δ 155.9, 144.0, 130.0, 129.4, 126.7, 118.9, 113.0, 112.9, 79.1, 70.7, 68.5, 63.9, 48.6, 44.3, 28.5 (3C), 25.9 (3C), 18.2, -4.7, -4.8. HRMS (ES+) m/z 457.2488 $[M+Na]^+$ $C_{23}H_{38}N_2O_4SiNa$ requires 457.2493 (Diff 1.16 ppm).

Spectroscopic data are in agreement with the literature.[2]

N-(((2*R*,3*S*,6*R*)-3-((*tert*-butyldimethylsilyl)oxy)-1,3,4,6-tetrahydro-2H-2,6-methanobenzo[c][1,5]oxazocin-8-yl)methyl)-1H-imidazole-1-carboxamide (**17**)

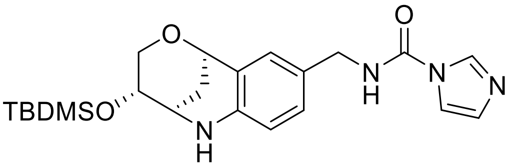

Tert-butyl(((2*R*,3*S*,6*R*)-3-((*tert*-butyldimethylsilyl)oxy)-1,3,4,6-tetrahydro-2H-2,6-methanobenzo[c][1,5]oxazocin-8-yl)methyl)carbamate (**16**) (0.15 g, 0.35 mmol, 1.00 eq) was

dissolved in a mixture of DCM:TFA (5:1, 5 mL) and the reaction mixture was allowed to stir at room temperature. The reaction was monitored by TLC. Upon completion, the reaction mixture diluted with DCM (10 ml) and neutralised with sat. $NaHCO_3$ (10 ml) and brine (10 ml) and extracted with DCM (3 x 20 ml) then dried over $MgSO_4$ and concentrated *in vacuo* to afford the free amine as a white solid (0.10 g, 94%) and it was used directly without further purification.

A solution of the free amine (0.10 g, 0.30 mmol, 1 eq) and DMAP (3.7 mg, 0.03 mmol, 0.1 eq) in DCM (2.5 mL) was cooled to 0°C, followed by slow addition of CDI (54 mg, 0.33 mmol. 1.1 eq). The reaction mixture was allowed to stir at room temperature overnight (followed by TLC). The reaction mixture was concentrated *in vacuo* and redissolved in EtOAc (20 mL) and washed with distilled water (15 mL), brine (10 mL) then dried over $MgSO_4$ and concentrated *in vacuo*. The crude product was purified by FCC (SiO_2; 50:50 to 70:30 EtOAc:Hexane) to afford the title compound as a yellow solid (0.06 g, 47%). ^{1}H NMR (400 MHz; MeOD) δ 8.21 (s, 1H), 7.57 (t, *J* =1.3 Hz, 1H), 7.07 (dd, *J* =8.3, 2.0 Hz, 1H), 7.02 (d, *J* =1.9 Hz, 1H), 6.98 (s, 1H), 6.51 (d, *J* =8.3 Hz, 1H), 4.59 (s, 1H), 4.33 (d, *J* =1.5 Hz, 2H), 3.46-3.17 (m, 4H), 2.61-2.52 (m, 1H), 1.35(d, *J* =12.6 Hz, 1H), 0.90 (s, 9H), 0.06 (s, 6H). ^{13}C NMR (100 MHz; $CDCl_3$) δ 148.88, 144.63, 135.97, 130.92, 130.48, 130.15, 125.12, 119.27, 116.19, 113.34, 70.67, 68.59, 64.04, 48.73, 44.84, 25.97 (3C), 23.48, 18.31, -4.56, -4.68; HRMS (ES+) m/z 429.2318 $[M+Na]^+$ $C_{22}H_{32}N_4O_3SiNa$ requires 429.2316 (Diff = 0.47 ppm).

1-(2-(2-(2-bromophenyl)pyrazolidin-1-yl)-2-oxoethyl)-3-(((2R,3S,6R)-3-((*tert*-butyldimethylsilyl)oxy)-1,3,4,6-tetrahydro-2H-2,6-methanobenzo[c][1,5]oxazocin-8-yl)methyl)urea

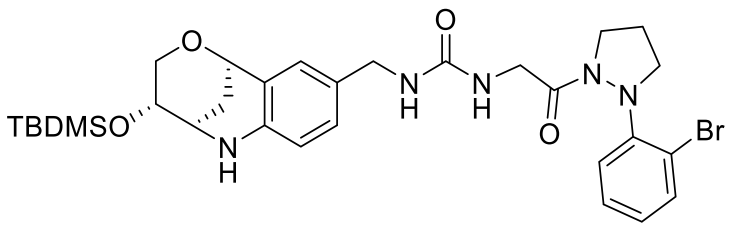

Tert-butyl(2-(2-(2-bromophenyl)pyrazolidin-1-yl)-2-oxoethyl)carbamate (**8a**) (383 mg, 0.43 mmol) was dissolved in a mixture of DCM:TFA (5:1, 10 mL) and the reaction mixture was allowed to stir at room temperature until TLC showed disappearance of starting material. The reaction mixture was

concentrated *in vacuo* to afford the free amine as a trifluoroacetate salt and was used directly without further purification.

To a solution of the trifluoroacetate salt (0.056 g, 0.12 mmol, 1.00 eq) in anhydrous acetonitrile (4.3 mL), was added *N*-(((2*R*,3*S*,6*R*)-3-((*tert*-butyldimethylsilyl)oxy)-1,3,4,6-tetrahydro-2H-2,6-methanobenzo[c][1,5]oxazocin-8-yl)methyl)-1H-imidazole-1-carboxamide (200 mg, 0.43 mmol, 1.1 eq), triethylamine (0.25 mL, 1.71 mmol, 2.2 eq) and DMAP (5.2 mg, 0.043 mmol, 0.1 eq) and was allowed to stir at room temperature overnight under N_2 (followed by TLC). Upon completion, the solution was extracted with ethyl acetate, washed with distilled water and brine, dried over $MgSO_4$ and concentrated *in vacuo*. The crude product was purified by FCC (SiO_2; DCM:MeOH = 20:1) to yield a yellow solid (200 mg, 73%).

^{1}H NMR (400 MHz; $CDCl_3$) δ 7.54 (dd, *J* =7.9, 1.2 Hz, 1H), 7.2-7.12 (m, 1H), 7.05(dd, *J* =8.2, 1.9 Hz, 1H), 7.01(d, *J* = 0.7 Hz, 1H), 6.95 (td, *J* =7.7, 1.4 Hz, 1H), 6.85 (dd, *J* =8.0, 1.2 Hz, 1H), 6.45 (d, *J* =4.6 Hz, 1H), 5.61 (t, *J* =4.6 Hz, 1H), 5.43 (t, *J* =5.4, 2.0 Hz, 1H), 4.63 (s, 1H), 4.38 (d, *J* =2.7 Hz, 1H), 4.20 (d, *J* =5.4 Hz, 2H), 3.71-3.37 (m, 9H), 3.32 (dd, *J* =12.7, 1.4 Hz, 1H), 2.62 (dd, *J* =8.2, 2.6 Hz, 1H), 2.05-1.88 (m, 2H), 1.39 (d, *J* =13.4 Hz, 1H), 0.91 (s, 9H), 0.06 (s, 6H) . ^{13}C NMR (100 MHz) δ171.08, 158.13, 148.27, 143.82, 134.34, 130.12, 129.45, 128.12, 127.82, 126.06, 119.24, 118.96, 115.90, 113.03, 70.82, 68.54, 63.92, 55.90, 48.71, 45.42, 43.99, 42.48, 25.9 (3C), 23.64, 23.09, 18.25, -4.61, -4.74; HRMS (ES+) $C_{30}H_{42}{}^{79}BrN_5O_4Na$ $[M+Na]^+$ requires 666.2082, found 666.2083 (Diff 0.15 ppm).

1-(2-(2-(2-Bromophenyl)pyrazolidin-1-yl)-2-oxoethyl)-3-(((2R,3S,6R)-3-hydroxy-1,3,4,6-tetrahydro-2H-2,6-methanobenzo[c][1,5]oxazocin-8-yl)methyl)urea **(18a)**

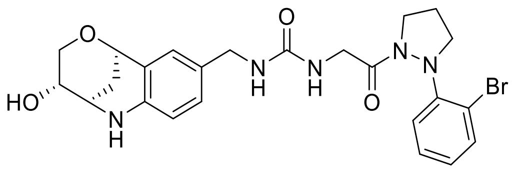

To a solution of 1-(2-(2-(2-bromophenyl)pyrazolidin-1-yl)-2-oxoethyl)-3-(((2R,3S,6R)-3-((*tert*-butyldimethylsilyl)oxy)-1,3,4,6-tetrahydro-2H-2,6-methanobenzo[c][1,5]oxazocin-8-yl)methyl)urea (0.15 g, 0.23 mmol, 1.00 eq) in THF (2.3 ml), *tetra-n*-butylammonium fluoride in 1M THF (0.46ml, 0.46 mmol, 2.00 eq) was added dropwise. The reaction mixture was allowed to stir at room temperature overnight. EtOAc (5 ml) was added to the reaction mixture and washed with distilled water (10ml), brine (10 ml) then dried over MgSO4 and concentrated *in vacuo* to afford the crude product. The crude product was purified by FCC (SiO_2; 95:5 EtOAc:MeOH) to yield a white solid (97 mg, 80%) M.p.: 137.2-139°C; ^{1}H NMR (400 MHz, $CDCl_3$) δ 7.54 (dd, *J* = 7.9, 1.2 Hz, 1H), 7.23

– 7.13 (m, 1H), 7.06 – 6.99 (m, 1H), 6.99 – 6.91 (m, 2H), 6.89 (dd, *J* = 8.0, 1.1 Hz, 1H), 6.45 (d, *J* = 8.2 Hz, 1H), 5.66 – 5.58 (m, 1H), 5.57 – 5.43 (m, 1H), 4.84 – 4.56 (m, 1H), 4.56 (s, 1H), 4.17 (d, *J* = 4.3 Hz, 2H), 3.92 – 3.60 (m, 3H), 3.57 – 3.52 (m, 1H), 3.50 – 3.39 (m, 4H), 3.33 (d, *J* = 12.2 Hz, 1H), 2.64 – 2.44 (m, 1H), 2.06 – 1.92 (m, 2H), 1.48 – 1.33 (m, 1H). ^{13}C NMR (101 MHz, $CDCl_3$) δ 171.9, 158.3, 148.3, 144.2, 134.4, 130.1, 129.6, 128.2, 127.5, 126.1, 119.3, 118.3, 115.9, 113.2, 69.9, 68.9, 63.6, 56.0, 47.8, 45.6, 44.0, 42.4, 23.8, 23.1.; HRMS (ES+) $C_{24}H_{28}{}^{79}BrN_5O_4Na$ [M+Na]$^+$ requires 552.1217, found 552.1220 (Diff 0.54 ppm); Purity HPLC 95.1%, Rt = 7.60 min (UV254 nm).

1-(((2*R*,3*S*,6*R*)-3-((*tert*-butyldimethylsilyl)oxy)-1,3,4,6-tetrahydro-2H-2,6-methanobenzo [c][1,5]oxazocin-8-yl)methyl)-3-(2-(2-(2-chloro-5-fluorophenyl)pyrazolidin-1-yl)-2-oxoethyl)urea

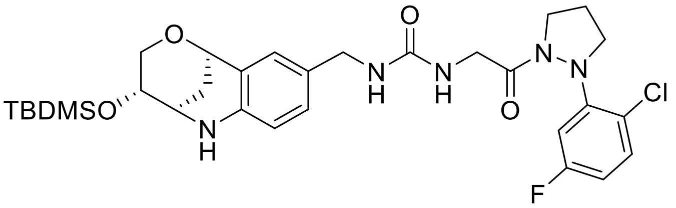

The title compound was obtained following a similar procedure as described for 1-(2-(2-(2-bromophenyl)pyrazolidin-1-yl)-2-oxoethyl)-3-(((2R,3S,6R)-3-((*tert*-butyldimethylsilyl)oxy)-1,3,4,6-tetrahydro-2H-2,6-methanobenzo[c][1,5]oxazocin-8-yl)methyl)urea starting from *tert*-butyl(2-(2-(2-chloro-5-fluorophenyl)pyrazolidin-1-yl)-2-oxoethyl)carbamate (**8c**) (120 mg, 0.33 mmol, 1 eq). Purification by column chromatography (DCM: MeOH= 20:1) gave the title compound (152 mg, 73%) as yellow solid. R_f = 0.60, 10% ethyl acetate in hexane. The crude product was purified by FCC (SiO_2; 95:5 DCM:MeOH) to give the title compound as a yellow solid (285 mg, 55%). ^{1}H NMR (400 MHz; $CDCl_3$) δ 7.31 (dt, *J* =12.3, 6.2 Hz, 1H), 7.11-7.01(m, 2H), 6.80-6.63(m, 2H) 6.47 (d, *J* =15.0 Hz, 1H), 5.34 (t, *J* =4.4 Hz, 1H), 5.05 (t, *J* =5.4 Hz, 1H), 4.66 (s, 1H), 4.32 (d, *J* =3.4 Hz, 1H), 4.22 (d, *J* =5.4 Hz, 2H), 3.54-3.40 (m, 10H), 3.34 (dd, *J* =12.9,1.6, 1H), 2.65 (d, *J* =12.9 Hz, 1H), 2.11-1.99 (m, 2H), 1.42 (d, *J* =12.6 Hz, 1H), 0.92(s, 9H), 0.08 (s, 3H), 0.07 (s, 3H). Glycan NH not observed.; ^{13}C NMR (101 MHz; $CDCl_3$) δ 172.1 , 161.8 (d, *J* =247.6 Hz) 157.9, 148.8, 143.9, 132.3 (d, *J* =9.0 Hz), 130.2, 129.5, 127.6, 120.5, 119.1, 112.3 (d, *J* = 23.1 Hz), 106.5 (d, *J* = 26.4 Hz), 77.5, 70.9, 68.6, 64.0, 55.4, 48.8, 45.1, 44.2, 42.5, 26.0 (3C), 23.7, 23.6, 18.3, -4.6, -4.7. HRMS (ES+) m/z 640.2484 [M+Na]$^+$ $C_{30}H_{42}ClFN_5O_4Na$ requires 640.2493 (Diff = -1.41 ppm).

1-(2-(2-(2-chloro-5-fluorophenyl)pyrazolidin-1-yl)-2-oxoethyl)-3-(((2*R*,3*S*,6*R*)-3-hydroxy-1,3,4,6-tetrahydro-2H-2,6-methanobenzo[c][1,5]oxazocin-8-yl)methyl)urea **(18b)**

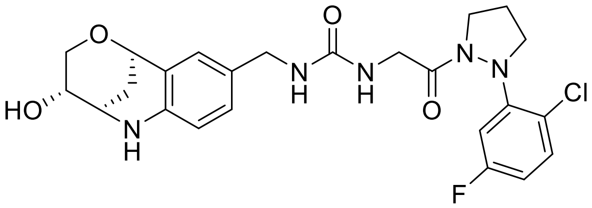

The title compound was obtained following a similar procedure to 1-(2-(2-(2-Bromophenyl)pyrazolidin-1-yl)-2-oxoethyl)-3-(((2R,3S,6R)-3-hydroxy-1,3,4,6-tetrahydro-2H-2,6-methanobenzo[c][1,5]oxazocin-8-yl)methyl)urea (**18a**) starting from 1-(((2*R*,3*S*,6*R*)-3-((*tert*-butyldimethylsilyl)oxy)-1,3,4,6-tetrahydro-2H-2,6-methanobenzo [c][1,5]oxazocin-8-yl)methyl)-3-(2-(2-(2-chloro-5-fluorophenyl)pyrazolidin-1-yl)-2-oxoethyl)urea (500 mg, 0.81 mmol). The crude product was purified by FCC (SiO_2; 95:5 EtOAc:MeOH) to give the title compound as a yellow solid (346 mg, 85%). M.p.: 145-146°C; ^{1}H NMR (400 MHz, $CDCl_3$) δ 7.32 (dd, *J* = 8.6, 5.6 Hz, 1H), 7.05 (d, *J* = 8.2 Hz, 1H), 6.97 (s, 1H), 6.80 – 6.72 (m, 1H), 6.70 (d, *J* = 9.7 Hz, 1H), 6.49 (d, *J* = 8.2 Hz, 1H), 5.63 – 5.50 (m, 1H), 5.47 – 5.40 (m, 1H), 4.59 (s, 1H), 4.49 – 4.30 (m, 1H), 4.19 (s, 2H), 3.96 – 3.55 (m, 5H), 3.53 – 3.40 (m, 3H), 3.35 (d, *J* = 12.6 Hz, 1H), 2.61 – 2.43 (m, 1H), 2.11 – 1.93 (m, 2H), 1.47 – 1.39 (m, 1H). ^{13}C NMR (101 MHz, $CDCl_3$) δ 172.3, 161.8 (d, *J* = 247.6 Hz), 158.2, 148.7 (d, *J* = 7.3 Hz), 144.1, 132.3 (d, *J* = 9.2 Hz), 130.1, 129.6, 127.6, 120.5 (d, *J* = 3.5 Hz), 118.3, 113.3, 112.4 (d, *J* = 23.2 Hz), 106.6 (d, *J* = 26.3 Hz), 70.0, 68.9, 63.7, 55.5, 47.8, 45.1, 44.1, 42.4, 23.8, 23.5.. HRMS (ES+) m/z 526.1623 $[M+Na]^+$ $C_{24}H_{27}ClFN_5O_4Na$ requires 526.1628 (Diff = -0.95 ppm); Purity HPLC 95.6%, Rt = 7.64 (UV254 nm).

1-(((2R,3S,6R)-3-((*tert*-butyldimethylsilyl)oxy)-1,3,4,6-tetrahydro-2H-2,6-methanobenzo[c][1,5]oxazocin-8-yl)methyl)-3-(2-(2-(2-chloro-5-methoxyphenyl)pyrazolidin-1-yl)-2-oxoethyl)urea

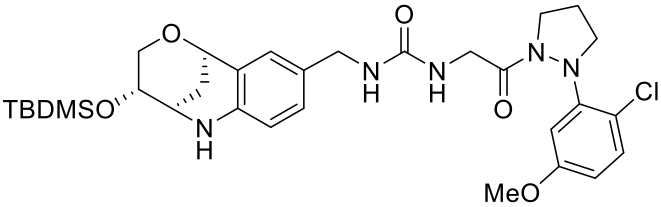

The title compound was obtained following a similar procedure as described for 1-(2-(2-(2-bromophenyl)pyrazolidin-1-yl)-2-oxoethyl)-3-(((2R,3S,6R)-3-((*tert*-butyldimethylsilyl)oxy)-1,3,4,6-tetrahydro-2H-2,6-methanobenzo[c][1,5]oxazocin-8-yl)methyl)urea starting from *tert*-butyl2-(2-chloro-5-methoxyphenyl)pyrazolidine-1-carboxylate (**8d**) (0.5 g, 1.35 mmol). The crude

product was purified by FCC (SiO_2; 95:5 DCM:MeOH) to give the title compound as a white solid (0.6 g, 67%). ^{1}H NMR (400 MHz; $CDCl_3$) δ 7.26 (d, *J* = 8.7 Hz, 1H), 7.07(dd, *J* = 8.2, 2.0 Hz, 1H), 7.04 (d, *J* = 1.9 Hz, 1H) 6.56 (dd, *J* = 8.7, 2.9 Hz, 1H), 6.50 (d, *J* = 2.9 Hz, 1H), 6.48 (d, *J* = 8.2 Hz, 1H), 5.13 (t, *J* =4.4 Hz, 1H), 4.76 (t, *J* = 4.5 Hz, 1H), 4.67 (s, 1H), 4.31 (s, 1H,), 4.22 (d, *J* = 5.5 Hz, 2H), 3.74 (s, 3H), 3.73-3.41 (m, 9H), 3.36 (dd, *J* = 13,1.6 Hz, 1H), 2.68-2.62 (m, 1H), 2.06-2.03 (br s, 2H), 1.44 (d, *J* = 13.5 Hz, 1H), 0.92 (s, 9H), 0.08 (s, 3H), 0.07 (s, 3H). ^{13}C NMR (100 MHz; $CDCl_3$) δ 171.6, 159.1, 157.0, 148.0, 144.0, 131.8, 130.2, 129.4, 127.4, 119.2, 117.1, 113.1, 109.9, 105.7, 70.8, 68.6, 64.0, 55.7, 55.5, 48.8, 45.2, 44.4, 42.5, 26.0 (3C), 23.7, 23.5, 18.3, -4.5, -4.7; HRMS (ES+) m/z 630.2873 $[M+H]^+$ $C_{31}H_{45}ClN_5O_5Si$ requires 630.2873 (Diff = -0.07 ppm).

1-(2-(2-(2-chloro-5-methoxyphenyl)pyrazolidin-1-yl)-2-oxoethyl)-3-(((2*R*,3*S*,6*R*)-3-hydroxy-1,3,4,6-tetrahydro-2H-2,6-methanobenzo[c][1,5]oxazocin-8-yl)methyl)urea (**18c**)

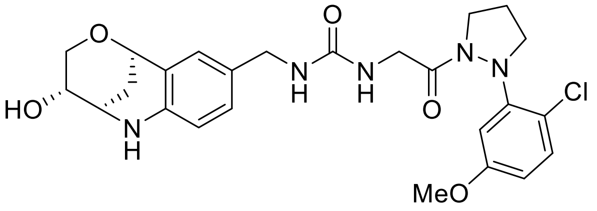

The title compound was obtained following a similar procedure to 1-(2-(2-(2-Bromophenyl)pyrazolidin-1-yl)-2-oxoethyl)-3-(((2R,3S,6R)-3-hydroxy-1,3,4,6-tetrahydro-2H-2,6-methanobenzo[c][1,5]oxazocin-8-yl)methyl)urea (18a) starting from 1-(((2R,3S,6R)-3-((*tert*-butyldimethylsilyl)oxy)-1,3,4,6-tetrahydro-2H-2,6-methanobenzo[c][1,5]oxazocin-8-yl)methyl)-3-(2-(2-(2-chloro-5-methoxyphenyl)pyrazolidin-1-yl)-2-oxoethyl)urea (0.5 g, 0.80 mmol). The crude product was purified by FCC (SiO_2; 96:4 DCM:MeOH) to give the title compound as a yellow solid (0.3 g, 75%). M.p. 153-154°C; ^{1}H NMR (400 MHz, MeOD) δ 7.33 (d, *J* = 8.8 Hz, 1H), 7.03 (dd, *J* = 8.3, 2.1 Hz, 1H), 6.98 (d, *J* = 1.9 Hz, 1H), 6.69 (dd, *J* = 8.8, 2.8 Hz, 1H), 6.59 (d, *J* = 2.8 Hz, 1H), 6.53 (d, *J* = 8.2 Hz, 1H), 4.71 – 4.59 (m, 1H), 4.20 – 4.08 (m, 2H), 3.98 – 3.34 (m, 13H), 2.67 – 2.53 (m, 1H), 2.14 – 2.02 (m, 2H), 1.49 – 1.38 (m, 1H). ^{13}C NMR (101 MHz, DMSO) δ 171.1, 158.8, 157.8, 148.3, 144.6, 131.3, 129.2, 128.5, 126.6, 117.8, 115.7, 112.2, 110.2, 105.6, 68.8, 67.9, 63.0, 55.5, 55.1, 47.1, 44.6, 42.6, 41.4, 23.3, 23.1.HRMS (ES+) m/z 538.1827 $[M+Na]^+$ $C_{25}H_{30}ClN_5O_5Na$ requires 538.1828 (Diff = 0.19 ppm). Purity HPLC 97.3%, Rt = 7.68 min (UV254 nm).

1-((*S*)-2-(2-(2-Bromophenyl)pyrazolidin-1-yl)-2-oxo-1-phenylethyl)-3-(((2*R*, 3*S*, 6*R*)- 3-((*tert*-butyldimethylsilyl)oxy)-1,3,4,6-tetrahydro-2H-2,6- methanobenzo[c][1,5]oxazocin-8-yl)methyl)urea

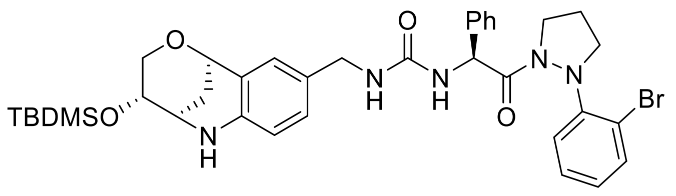

Tert-butyl(*S*)-(2-(2-(2-bromophenyl)pyrazolidin-1-yl)-2-oxo-1- phenylethyl)carbamate (0.054 g, 0.12 mmol, 1.00 eq) was dissolved in a mixture of DCM:TFA (5:1, 2.5 mL) and the reaction mixture was allowed to stir at room temperature until TLC showed disappearance of starting material. The reaction mixture was concentrated *in vacuo* to afford the free amine as a trifluoroacetate salt and was used directly without further purification. To a solution of the trifluoroacetate salt (0.056 g, 0.12 mmol, 1.00 eq) in anhydrous acetonitrile (3 mL), was added *N*-(((2*R*,3*S*,6*R*)-3-((*tert*-butyldimethylsilyl)oxy)-1,3,4,6-tetrahydro-2H-2,6-methanobenzo[c][1,5]oxazocin-8-yl)methyl)-1H-imidazole-1-carboxamide (56 mg, 0.13 mmol, 1.1 eq), triethylamine (0.04 mL, 0.26 mmol, 2.2 eq) and DMAP (1.6 mg, 0.01 mmol, 0.1 eq) and was allowed to stir at room temperature overnight under N_2 (followed by TLC). Upon completion, the solution was extracted with ethyl acetate, washed with distilled water and brine, dried over $MgSO_4$ and concentrated *in vacuo*. The crude product was purified by FCC (SiO2; EtOAc) to yield a yellow solid (0.03 g, 31%). ^{1}H NMR (500MHz; $CDCl_3$; Me_4Si; ppm) δ 7.52-7.39 (m, 1H), 7.36-7.29 (m, 2H), 7.09-7.01 (m, 2H), 6.91-6.86 (m, 2H), 6.66 (s, 2H), 6.44-6.42 (m, 1H), 6.16 (s, 2H), 5.32 (s, 1H), 5.24 (s, 1H), 4.60 (s, 1H), 4.31 (s, 1H), 4.29-4.12 (m, 2H), 3.79-3.32 (m, 8H), 3.24-3.23 (m, 1H), 2.63-2.60 (m, 1H), 2.04-1.76 (m, 2H), 1.39-1.36 (m, 1H), 0.91 (s, 9H), 0.06 (s, 3H), 0.05 (s, 3H); ^{13}C NMR (126 MHz; $CDCl_3$; Me_4Si) δ 174.01, 157.52, 147.94, 147.86, 144.19, 137.94, 134.90, 133.81, 130.72, 130.00, 128.52, 128.22, 127.88, 127.61, 126.17, 125.41, 119.51, 119.34, 115.65, 113.32, 77.68, 71.16, 68.83, 64.26, 56.36, 53.87, 49.07, 45.87, 44.46, 26.28 (3C), 23.97, 18.61, -4.26, -4.40; HRMS (ES+) 720.2572 $[M+H]^+$ $C_{36}H_{47}N_5O_4{}^{79}BrSi$ requires 720.2575 (Diff 0.45 ppm).

1-((*S*)-2-(2-(2-Bromophenyl)pyrazolidin-1-yl)-2-oxo-1-phenylethyl)-3-(((2*R*,3*S*,6*R*)-3- hydroxy-1,3,4,6-tetrahydro-2H-2,6-methanobenzo[c][1,5]oxazocin-8-yl)methyl)urea **(18d)**

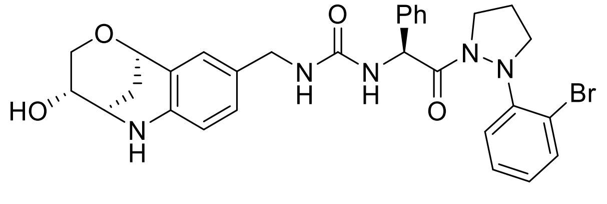

To a solution of 1-((*S*)-2-(2-(2-bromophenyl)pyrazolidin-1-yl)-2-oxo-1-phenylethyl)-3- (((2*R*, 3*S*,6*R*)-3-((*tert*-butyldimethylsilyl)oxy)-1,3,4,6-tetrahydro-2H-2,6- methanobenzo[c][1,5]oxazocin-8-yl)methyl)urea (**9a**) (0.02 g, 0.03 mmol, 1.00 eq) in THF (1.6 ml), *tetra-N*-butylammonium fluoride in 1M THF (0.02ml, 0.06 mmol, 2.00 eq) was added dropwise. The reaction mixture was allowed to

stir at room temperature overnight. EtOAc (5 ml) was added to the reaction mixture and washed with distilled water (10ml), brine (10 ml) then dried over $MgSO_4$ and concentrated *in vacuo* to afford the crude product. The crude product was purified by FCC (SiO_2; 95:5 EtOAc:MeOH) to yield a yellow solid (0.015 g, 79%). M.p. 132-136 °C; ^{1}H NMR (400 MHz, DMSO) δ 7.75 – 7.01 (m, 6H), 6.96 – 6.76 (m, 5H), 6.59 – 6.48 (m, 1H), 6.47 – 6.42 (m, 1H), 6.37 (t, *J* = 5.5 Hz, 1H), 6.28 (s, 1H), 5.95 (d, *J* = 8.3 Hz, 1H, rotamer 2), 5.48 (d, *J* = 7.3 Hz, 1H, rotamer 1), 4.94 (d, *J* = 3.8 Hz, 1H), 4.49 (s, 1H), 4.06 – 3.84 (m, 2H), 3.77 – 3.35 (m, 5H), 3.28 – 3.23 (m, 1H), 3.13 (d, *J* = 12.3 Hz, 1H), 2.46 – 2.39 (m, 1H), 2.12 – 1.75 (m, 2H), 1.28 – 1.18 (m, 1H). IR *v*max/cm-1: 3332 (N-H), 1618 (urea C=O), 1508 (N-H), 610 (C-Br); HRMS (ES+) 606.1707 $[M+H]^+$ $C_{30}H_{33}N_5O_4{}^{79}Br$ requires 606.1710 (Diff 1.69 ppm); $[\alpha]^{20}_D$ = 2.5 (*c* 1.0, CH_3OH); Purity HPLC 95.3%, Rt = 8.85 min (UV254 nm).

1-(((2*R*,3*S*,6*R*)-3-((*tert*-butyldimethylsilyl)oxy)-1,3,4,6-tetrahydro-2H-2,6-methanobenzo[c][1,5]oxazocin-8-yl)methyl)-3-((*S*)-2-(2-(2-chloro-5-methoxyphenyl)pyrazolidin-1-yl)-2-oxo-1-phenylethyl)urea

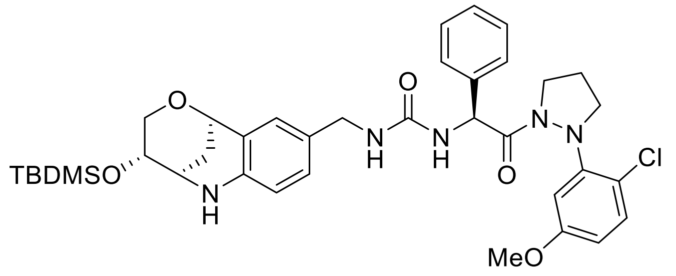

The title compound was obtained following a similar procedure as described for 1-(2-(2-(2-bromophenyl)pyrazolidin-1-yl)-2-oxoethyl)-3-(((2R,3S,6R)-3-((*tert*-butyldimethylsilyl)oxy)-1,3,4,6-tetrahydro-2H-2,6-methanobenzo[c][1,5]oxazocin-8-yl)methyl)urea starting from *tert*-butyl(S)-(2-(2-(2-chloro-5-methoxyphenyl) pyrazolidin-1-yl)-2-oxo-1-phenylethyl)Carbamate (**9b**) (0.3 g, 0.67 mmol). The crude product was purified by FCC (SiO_2; 95:5 DCM:MeOH) to give the title compound as a white solid (0.3 g, 64%). ^{1}H NMR (400 MHz; $CDCl_3$) δ 7.46 (s, 1H), 7.41-7.28 (m, 2H), 7.16-6.94 (m, 4H), 6.94-6.83 (m, 1H), 6.57-6.53 (m, 1H), 6.45-6.42 (m, 1H), 6.29 (d, *J* = 7.7 Hz, 1H), 6.07 (s, 1H), 5.64-5.59 (m, 1H), 5.03 (s, 1H), 4.61 (s, 1H), 4.31 (s, 1H), 4.25-4.19 (m, 2H), 3.77 (d, *J* = 6.2 Hz, 1H), 3.50-3.26 (m, 10H), 2.62 (d, *J* = 12.7 Hz, 1H), 1.76 (s, 2H), 1.43-1.33 (m, 1H), 0.92 (s, 9H), 0.07 (s, 3H), 0.06 (s, 3H). ^{13}C NMR (101 MHz; $CDCl_3$) δ 171.5, 156.9, 143.9, 130.4, 129.6, 129.3, 129.1, 128.4, 128.3, 128.2, 128.2, 128.0, 128.0, 127.5, 127.2, 127.1, 125.2, 119.1, 113.1, 110.0, 70.9, 68.5, 64.0, 56.0, 55.6, 55.4, 48.8, 45.1, 44.2, 26.0 (3C), 23.7, 23.2, 18.3, -4.5, -4.6. HRMS (ES+) m/z 728.3008 $[M+ Na]^+$ $C_{37}H_{48}ClN_5O_5SiNa$ requires 728.3005 (Diff = 0.41 ppm).

1-((*S*)-2-(2-(2-chloro-5-methoxyphenyl)pyrazolidin-1-yl)-2-oxo-1-phenylethyl)-3-(((2*R*,3*S*,6*R*)-3-hydroxy-1,3,4,6-tetrahydro-2H-2,6-methanobenzo[c][1,5]oxazocin-8-yl)methyl)urea **(18e)**

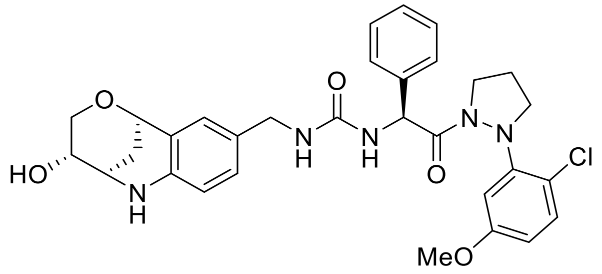

The title compound was obtained following a similar procedure to 1-(2-(2-(2-Bromophenyl)pyrazolidin-1-yl)-2-oxoethyl)-3-(((2R,3S,6R)-3-hydroxy-1,3,4,6-tetrahydro-2H-2,6-methanobenzo[c][1,5]oxazocin-8-yl)methyl)urea (**18a**) starting from 1-(((2*R*,3*S*,6*R*)-3-((*tert*-

butyldimethylsilyl)oxy)-1,3,4,6-tetrahydro-2H-2,6-methanobenzo[c][1,5]oxazocin-8-yl)methyl)-3-((*S*)-2-(2-(2-chloro-5-methoxyphenyl)pyrazolidin-1-yl)-2-oxo-1-phenylethyl)urea (230 mg, 0.32 mmol). The crude product was purified by FCC (SiO_2; 95:5 DCM:MeOH) to give the title compound as a yellow solid (150 mg, 79%). M.p. 68-69°C; ^{1}H NMR (400 MHz, $CDCl_3$) δ 7.47 (s, 1H), 7.33 – 7.22 (m, 1H), 7.14 – 7.02 (m, 2H), 7.11 – 6.81 (m, 4H), 6.63 – 5.97 (m, 4H), 5.62 (s, 1H), 5.57 – 5.39 (m, 1H), 4.51 (s, 1H), 4.30 – 4.05 (m, 2H), 3.98 – 3.31 (m, 11H), 3.28 (d, *J* = 12.7 Hz, 1H), 2.60 – 2.41 (m, 1H), 2.04 – 1.66 (m, 2H), 1.44 – 1.31 (m, 1H). HRMS (ES+) m/z 614.2143 $[M+ Na]^+$ $C_{31}H_{34}ClN_5O_5Na$ requires 614.2141 (Diff = 0.33 ppm); Purity HPLC 96.4%, Rt = 8.82 min (UV254 nm).

Tert-butyl (S)-(1-(4-bromophenyl)-2-(2-(2-chloro-5-fluorophenyl)pyrazolidin-1-yl)-2-oxoethyl)carbamate

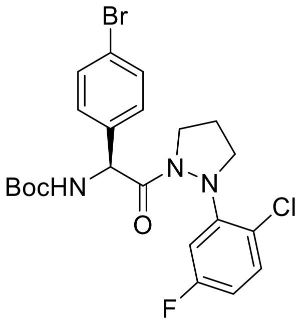

The title compound was obtained following a similar procedure as described for *tert*-butyl(2-(2-(2-bromophenyl)pyrazolidin-1-yl)-2-oxoethyl)carbamate (**8a**) starting from *tert*-butyl2-(2-chloro-5-fluorophenyl)pyrazolidine-1-carboxylate (0.5 g, 1.66 mmol, 1 eq) and (S)-2-(4-bromophenyl)-2-((tert-butoxycarbonyl)amino)acetic acid (0.55 g, 1.66 mmol, 1 eq). The crude product was purified by FCC (SiO_2, hexane: ethyl acetate = 3:1) to afford the title compound (300 mg, 35.2%) as a white solid R_f = 0.30, 30% hexane in ethyl acetate. ^{1}H NMR (400 MHz; MeOD) δ 7.45 (dd, *J* = 10.8, 8.5 Hz, 1H), 7.34-7.29 (m, 1H), 7.00 (d, *J* = 8.0 Hz, 1H), 6.94 (d, *J* = 7.8 Hz, 1H), 6.57 (t, *J* = 6.6 Hz, 1H), 5.94-5.89 (major, m, 1H), 5.85 (major, d, *J* = 9.8 Hz, 1H), 5.73 (minor, d, *J* = 7.2 Hz, 1H), 5.34-5.28 (minor, m, 1H), 4.13-4.04 (minor, m, 2H), 3.90-3.88 (minor, m, 2H), 3.61-3.51 (major, m, 2H), 3.42-3.35 (major, m, 2H), 2.06 (minor, br s, 2H), 1.88-1.86 (major, m, 2H), 1.37 (major, s, 9H), 1.33 (minor, s, 9H). ^{13}C NMR (101 MHz; MeOD) δ 174.04, 174.01, 162.72 (d, *J* = 266.7 Hz), 162.5 (d, *J* = 247.2 Hz), 157.33, 157.26, 149.73, 149.65, 136.66, 133.30, 132.64, 132.55, 132.38 (4C), 131.08 (4C), 130.43, 130.39, 127.76, 121.17, 112.71, 112.48, 108.11, 107.84, 57.53, 56.65, 46.34, 42.41,

35.16, 29.57, 28.68 (3C), 24.00 (3C), 23.94, 21.35. HRMS (ESI+) $C_{22}H_{24}{}^{79}Br^{35}ClFN_3O_3$ [M+ Na]$^+$ requires 534.0566, found 534.0570.

1-((S)-1-(4-bromophenyl)-2-(2-(2-chloro-5-fluorophenyl)pyrazolidin-1-yl)-2-oxoethyl)-3-(((2R,3S,6R)-3-((tert-butyldimethylsilyl)oxy)-1,3,4,6-tetrahydro-2H-2,6-methanobenzo[c][1,5]oxazocin-8-yl)methyl)urea

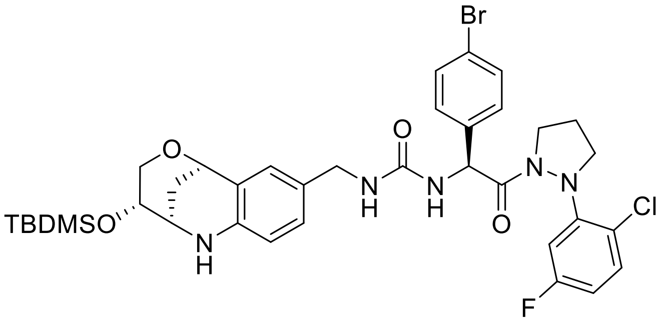

The title compound was obtained following a similar procedure as described for 1-(2-(2-(2-bromophenyl)pyrazolidin-1-yl)-2-oxoethyl)-3-(((2R,3S,6R)-3-((*tert*-butyldimethylsilyl)oxy)-1,3,4,6-tetrahydro-2H-2,6-methanobenzo[c][1,5]oxazocin-8-yl)methyl)urea starting from *tert*-butyl (S)-(1-(4-bromophenyl)-2-(2-(2-chloro-5-fluorophenyl)pyrazolidin-1-yl)-2-oxoethyl)carbamate (1 g, 1.95 mmol, 1 eq). Purification by column chromatography (DCM: MeOH= 20:1) gave the title compound (1.02 g, 68%) as a yellow solid. R_f = 0.50, 10% MeOH in DCM. ^{1}H NMR (400 MHz; MeOD) δ 7.40 (s, 1H), 7.29 (s, 1H), 7.19-7.16 (m, 1H), 6.98 (d, *J* = 7.6 Hz, 1H), 6.93-6.81 (m, 3H), 6.58 (t, *J* = 7.1 Hz, 1H), 6.44 (d, *J* = 8.0 Hz, 1H), 5.96 (d, *J* = 9.6 Hz, 1H), 4.52 (s, 1H), 4.10-3.90 (m, 3H), 3.73-3.24 (m, 8H), 2.52 (d, *J* = 12.6 Hz, 1H), 2.02-1.79 (m, 2H), 1.30 (d, *J* = 12.2 Hz, 1H), 0.88 (s, 9H), 0.04 (s, 6H). ^{13}C NMR (101 MHz; MeOD) δ 174.49, 159.88, 159.81, 149.73, 146.00, 137.52, 132.65, 132.38, 130.89, 130.66, 130.62, 130.23, 130.20, 127.89, 122.58, 121.21, 119.20, 114.03, 112.55 (d, *J* = 23.3 Hz), 107.94 (d, *J* = 27.3 Hz), 71.93, 70.38, 64.97, 56.88, 56.63, 46.49, 46.34, 44.41, 26.32 (3C), 24.52, 23.97, 18.97, -4.69, -4.76. HRMS (ESI+) $C_{36}H_{44}{}^{79}Br^{35}ClFN_5O_4SiNa$ [M+ Na]$^+$ requires 796.1916, found 796.1890.

1-((S)-2-(2-(2-chloro-5-fluorophenyl)pyrazolidin-1-yl)-1-(4'-(morpholinomethyl)-[1,1'-biphenyl]-4-yl)-2-oxoethyl)-3-(((2R,3S,6R)-3-hydroxy-1,3,4,6-tetrahydro-2H-2,6-methanobenzo[c][1,5]oxazocin-8-yl)methyl)urea

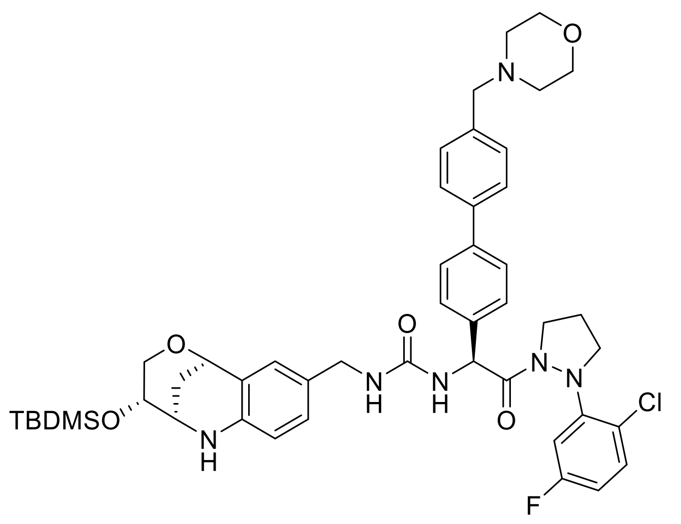

To a solution of 1-((S)-1-(4-bromophenyl)-2-(2-(2-chloro-5-fluorophenyl)pyrazolidin-1-yl)-2-oxoethyl)-3-(((2R,3S,6R)-3-((*tert*-butyldimethylsilyl)oxy)-1,3,4,6-tetrahydro-2H-2,6-methanobenzo[c][1,5]oxazocin-8-yl)methyl)urea (200 mg, 0.26 mmol, 1 eq) in anhydrous dioxane (4 ml) and H_2O (0.6 ml) was added [4-(Morpholinomethyl) phenyl] boronic acid (129.3 mg, 0.58 mmol, 2 eq), [1, 1′-Bis(diphenylphosphino) ferrocene]dichloropalladium(II) (20 mg, 0.026 mmol, 0.1 eq), K_2CO_3 (107.6 mg, 0.78 mmol, 3 eq) under N_2, the reaction was heated to reflux for 24h. The mixture was filtrated through Celite and extracted with DCM, washed with distilled water and brine, dried over $MgSO_4$ then concentrated *in vacuo* to afford crude product. Purification by column chromatography (DCM: MeOH= 30:1) gave the title compound (300 mg, 88%) as a white solid. R_f = 0.6, 10% MeOH in DCM. ^{1}H NMR (400 MHz; MeOD) δ 7.51 (s, 1H), 7.36 (s, 1H), 7.30 (s, 4H), 7.16-7.12 (m, 1H), 7.11-7.06 (m, 3H), 6.94 (d, *J* = 8.0 Hz, 1H), 6.88-6.80 (m, 1H), 6.46-6.39 (m, 1H), 5.97-5.92 (m, 1H), 4.52 (s, 1H), 4.13-3.93 (m, 2H), 3.63-3.61 (m, 4H), 3.59 (s, 2H), 3.55-3.47 (m, 4H), 3.42 (br s, 2H), 3.36 (br s, 1H), 3.27 (s, 1H), 3.25 (s, 1H), 2.51 (d, *J* = 12.5 Hz, 1H), 2.41 (s, 4H), 2.04-1.79 (m, 2H), 1.29 (d, *J* = 12.6 Hz, 1H), 0.88 (s, 9H), 0.04 (s, 3H), 0.03 (s, 3H). ^{13}C NMR (101 MHz; MeOD) δ 174.98, 162.40 (d, *J* = 247.5 Hz), 159.95, 149.86, 149.82, 145.98, 141.52, 141.04, 137.42, 137.15, 132.48, 132.40, 131.06, 130.62, 130.21, 129.63, 129.49, 127.91, 127.85 (2C), 121.21, 121.17, 119.20, 114.04, 112.38 (d, *J* = 20.5 Hz), 107.98 (d, *J* = 27.3 Hz), 71.92, 70.36, 68.11, 67.73 (2C), 64.95, 63.97, 57.23, 56.64, 54.64 (2C), 46.27, 44.44, 26.34 (3C), 24.50, 23.99, 18.97, -4.67, -4.75. HRMS (ESI+) $C_{47}H_{59}{}^{35}ClFN_6O_5Si$ [M+ H] $^+$ requires 869.3989, found 869.3979.

1-((S)-2-(2-(2-chloro-5-fluorophenyl)pyrazolidin-1-yl)-1-(4'-(morpholinomethyl)-[1,1'-biphenyl]-4-yl)-2-oxoethyl)-3-(((2R,3S,6R)-3-hydroxy-1,3,4,6-tetrahydro-2H-2,6-methanobenzo[c][1,5]oxazocin-8-yl)methyl)urea (18f)

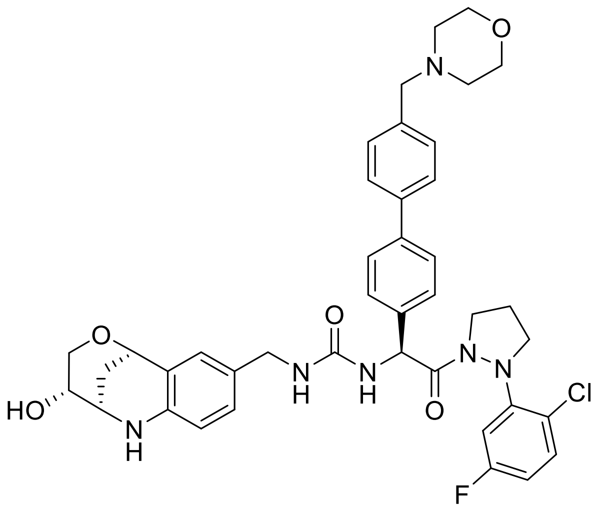

The title compound was obtained following a similar procedure to 1-(2-(2-(2-Bromophenyl)pyrazolidin-1-yl)-2-oxoethyl)-3-(((2R,3S,6R)-3-hydroxy-1,3,4,6-tetrahydro-2H-2,6-methanobenzo[c][1,5]oxazocin-8-yl)methyl)urea (**18a**) starting from

1-((S)-2-(2-(2-chloro-5-fluorophenyl)pyrazolidin-1-yl)-1-(4'-(morpholinomethyl)-[1,1'-biphenyl]-4-yl)-2-oxoethyl)-3-(((2R,3S,6R)-3-hydroxy-1,3,4,6-tetrahydro-2H-2,6-methanobenzo[c][1,5]oxazocin-8-yl)methyl)urea (300 mg, 0.34 mmol, 1 eq) Purification by column chromatography (DCM: MeOH= 15:1) gave the title compound (150 mg, 57.7%) as a white solid. R_f = 0.5, 10% MeOH in DCM. m.p. decomposed at 158°C. ^{1}H NMR (400 MHz, MeOD) δ 7.66 – 7.47 (m, 2H), 7.46 – 7.29 (m, 4H), 7.22 – 7.06 (m, 4H), 7.05 – 6.86 (m, 2H), 6.58 – 6.43 (m, 2H), 6.03 (d, *J* = 9.9 Hz, 1H), 5.98 (s, 1H), 4.59 (s, 1H), 4.22 – 3.98 (m, 3H), 3.77 – 3.32 (m, 13H), 2.59 – 2.52 (m, 1H), 2.49 (s, 4H), 2.14 – 1.82 (m, 2H), 1.42 – 1.34 (m, 1H). ^{13}C NMR (101 MHz, MeOD) δ 174.96, 162.40 (d, *J* = 246.6 Hz), 159.96, 149.82 (d, *J* = 8.9 Hz), 146.12, 141.51, 141.12, 137.21, 137.15, 132.45 (d, *J* = 9.2 Hz), 131.26, 131.12, 130.63, 130.19, 129.62, 129.50, 127.91, 127.87, 121.19 (d, *J* = 3.3 Hz), 119.19, 114.08, 112.38 (d, *J* = 23.1 Hz), 107.98 (d, *J* = 26.4 Hz), 70.86, 70.37, 67.66, 64.35, 63.91, 57.24, 56.64, 54.80, 54.60, 46.28, 44.44, 24.63, 23.99. HRMS (ESI+) $C_{41}H_{45}ClFN_6O_5$ [M+ H]$^+$ requires 755.3124, found 755.3120. Purity HPLC 98.0%, R_t = 7.68 min (UV254 nm).

1-(((2R,3S,6R)-3-((*tert*-butyldimethylsilyl)oxy)-1,3,4,6-tetrahydro-2H-2,6-methanobenzo[c][1,5]oxazocin-8-yl)methyl)-3-(2-((R)-2-(2-chloro-5-fluorophenyl)pyrrolidin-1-yl)-2-oxoethyl)urea

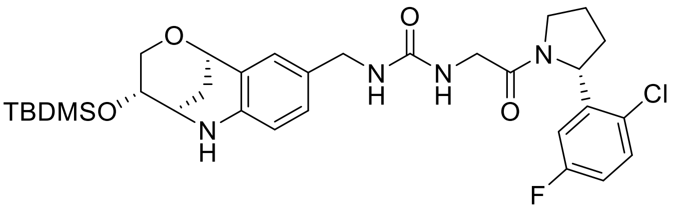

To a solution of *tert*-butyl(R)-(2-(2-(2-chloro-5-fluorophenyl)pyrrolidin-1-yl)-2-oxoethyl)carbamate (75 mg, 0.21 mmol) in dichloromethane (3 mL) was add trifluoroacetic acid (0.5 mL). The reaction mixture was allowed to stir at room temperature until disappearance of starting material (2 h, followed by TLC). Upon completion, the solvent was remove in vacuo to give the free amine as TFA salt which was used directly without further purification.

The TFA salt of the free amine was redissolved in anhydrous acetonitrile (15 mL), *N*-(((2*R*,3*S*,6*R*)-3-((*tert*-butyldimethylsilyl)oxy)-1,3,4,6-tetrahydro-2H-2,6-methanobenzo[c][1,5]oxazocin-8-yl)methyl)-1H-imidazole-1-carboxamide (100 mg, 0.23 mmol, 1.1 eq), triethylamine (0.06 mL, 0.46 mmol, 2.2 eq) and DMAP (3 mg, 0.02 mmol, 0.1 eq) were added. The reaction mixture was allowed to stir at room temperature overnight (followed by TLC). Upon completion, the reaction was diluted with dichloromethane, washed with water, followed by brine, dried over $MgSO_4$, filtered and concentrated *in vacuo* to a light brown oil. The crude product was purified by FCC (SiO_2; 4:96 MeOH:DCM) to afford the title compound as a white solid (90 mg, 69%). With the yield consisting of a mixture of rotamers in ratio 1/1.12.

^{1}H NMR (400 MHz, $CDCl_3$) δ 7.35 (dd, *J* = 8.8, 5.0 Hz, 1H, minor), 7.30 (dd, *J* = 8.8, 5.1 Hz, 1H, major), 7.09 – 7.00 (m, 2H), 6.98 – 6.91 (m, 1H, minor), 6.90 – 6.84 (m, 1H, majorj), 6.75 (dd, *J* = 9.0, 3.0 Hz, 1H, minor), 6.67 (dd, *J* = 9.2, 2.9 Hz, 1H, major), 6.47 (d, *J* = 8.2 Hz, 1H), 5.38 (dd, *J* = 8.1, 2.0 Hz, 1H, major), 5.33 (br s, 1H), 5.22 (d, *J* = 7.2 Hz, 1H, minor), 4.79 (t, *J* = 5.6 Hz, 1H, minor), 4.71 (t, *J* = 5.2 Hz, 1H, major), 4.67 (s, 1H), 4.31 (s, 1H), 4.23 (d, *J* = 5.5 Hz, 1H), 4.19 (d, *J* = 5.5 Hz, 1H), 4.12 (d, *J* = 4.0 Hz, 1H), 4.09 (d, *J* = 4.9 Hz, 1H, minor), 3.83 – 3.56 (m, 3H), 3.47 (d, *J* = 11.8 Hz, 3H), 3.41 – 3.30 (m, 2H), 2.66 (d, *J* = 12.5 Hz, 1H), 2.47 – 2.26 (m, 2H), 2.06 – 1.93 (m, 2H), 1.93 – 1.72 (m, 2H), 1.56 (d, *J* = 12.1 Hz, 1H), 1.44 (d, *J* = 14.6 Hz, 1H), 0.93 (s, 9H), 0.09 (s, 3H), 0.08 (s, 3H).

1-(2-((*R*)-2-(2-chloro-5-fluorophenyl)pyrrolidin-1-yl)-2-oxoethyl)-3-(((2R,3S,6R)-3-hydroxy-1,3,4,6-tetrahydro-2H-2,6-methanobenzo[c][1,5]oxazocin-8-yl)methyl)urea (**19**)

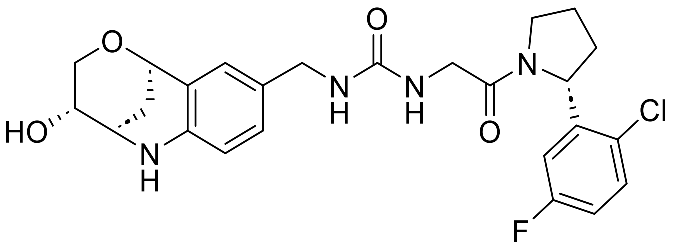

To a solution of 1-(((2R,3S,6R)-3-((*tert*-butyldimethylsilyl)oxy)-1,3,4,6-tetrahydro-2H-2,6-methanobenzo[c][1,5]oxazocin-8-yl)methyl)-3-(2-((R)-2-(2-chloro-5-fluorophenyl)pyrrolidin-1-yl)-2-oxoethyl)urea (80 mg, 0.13 mmol) in anhydrous THF (5 mL) was added tetrabutylammonium fluoride (1.0 M in THF, 0.26 mL, 0.26 mmol, 2 eq) dropwise. The reaction mixture was allowed to stir at room temperature for 16 h (followed by TLC). Upon completion, the reaction was diluted with ethyl acetate, washed with water and brine, dried over $MgSO_4$, filtered and concentrated *in vacuo* to a white solid. The crude product was purified by FCC (SiO_2; 5:95 MeOH:DCM) to afford the title compound as a white solid (48 mg, 73 %). With the yield consisting of a mixture of rotamers in ratio 1.21:1. ^{1}H NMR (400 MHz, $CDCl_3$) δ 7.35 (dd, *J* = 8.8, 5.0 Hz, 1H, minor), 7.29 (dd, *J* = 8.8, 5.1 Hz, 1H, major), 7.09 – 7.02 (m, 1H), 6.99 (dd, *J* = 11.0, 2.1 Hz, 1H), 6.97 – 6.92 (m, 1H, minor), 6.89 – 6.83 (m, 1H, major), 6.74 (dd, *J* = 9.0, 3.0 Hz, 1H, minor), 6.69 (dd, *J* = 9.2, 3.0 Hz, 1H, major), 6.49 (d, *J* = 1.6 Hz, 1H), 6.47 (d, *J* = 1.6 Hz, 1H), 5.63 – 5.52 (m, 1H), 5.42 – 5.34 (m, 1H, major), 5.28 – 5.22 (m, 1H, minor), 5.19 – 5.08 (m, 1H), 4.65 (s, 1H), 4.47 – 4.35 (m, 1H), 4.26 – 4.02 (m, 3.5H, minor), 3.83 – 3.68 (m, 2H), 3.67 – 3.28 (m, 5.5 H, major), 2.70 (t, *J* = 7.3 Hz, 1H), 2.60 – 2.50 (m, 1H), 2.47 – 2.26 (m, 1H), 2.04 – 1.88 (m, 2H), 1.91 – 1.75 (m, 1H), 1.54 – 1.43 (m, 1H). ^{13}C NMR (101 MHz, $CDCl_3$) δ 168.39, 167.83, 161.67 (d, *J* = 248.1 Hz), 161.48 (d, *J* = 246.3 Hz), 156.16, 155.85, 141.81 (d, *J* = 6.7 Hz), 141.41 (d, *J* = 6.7 Hz), 131.69 (d, *J* = 8.0 Hz), 131.18 (d, *J* = 8.2 Hz),126.73 (d, *J* = 3.1 Hz), 126.63 (d, *J* = 3.1 Hz), 116.07 (d, *J* = 22.9 Hz), 115.15 (d, *J* = 23.0 Hz), 113.36 (d, *J* = 24.4 Hz), 113.23 (d, *J* = 24.2 Hz), 84.74, 79.94, 79.79, 59.11, 58.42, 47.69, 46.92, 45.22, 43.20, 42.85, 42.27, 40.96, 34.04, 31.93, 27.86, 23.92, 23.52, 21.17, 21.05. HRMS (ES+) 503.1852 $[M+H]^+$ $C_{25}H_{29}N_4O_4FCl$ requires 503.1856 (Diff = -0.79 ppm); Purity HPLC 95.1%, Rt = 7.82 min (UV 254 nm).

3. Analytical Data/ NMR/HPLC traces

^{1}H NMR spectrum of **4b**

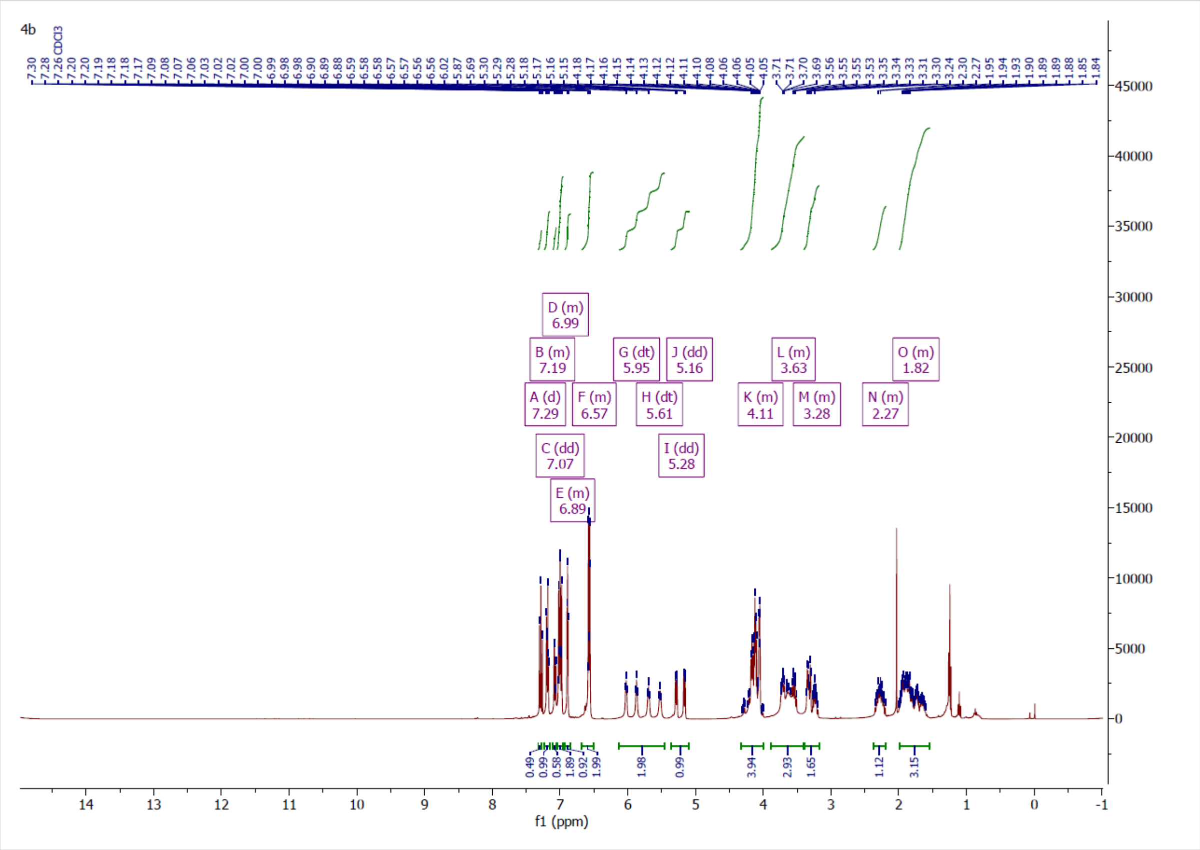

^{13}C NMR spectrum of **4b**

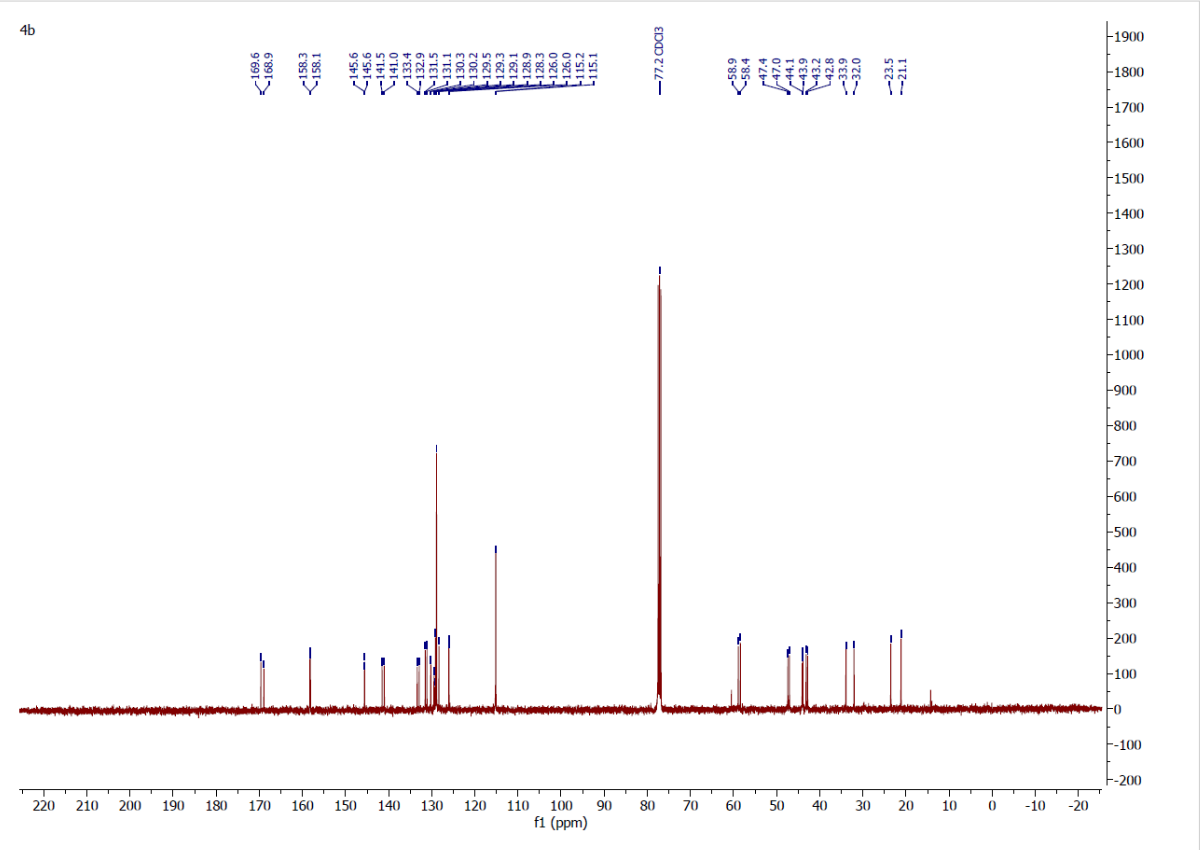

^{1}H NMR spectrum of **4c**

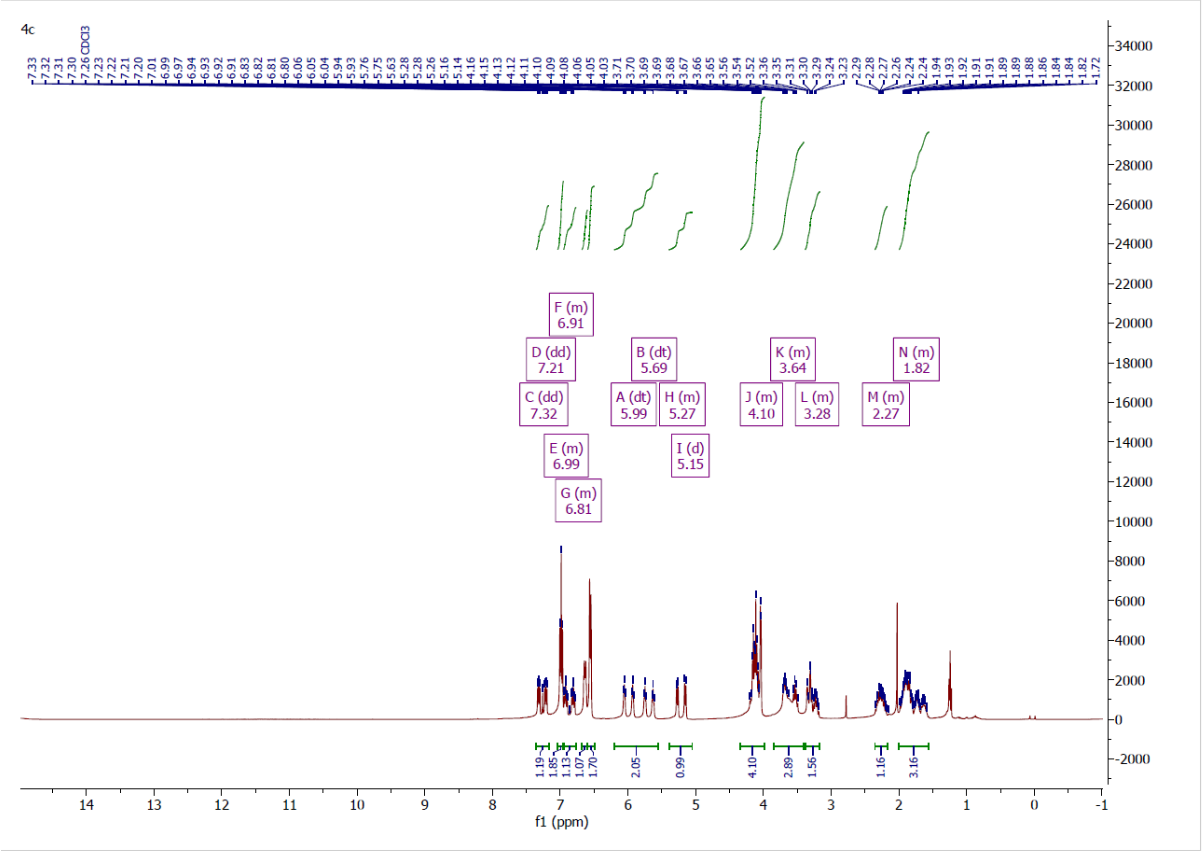

^{13}C NMR spectrum of **4c**

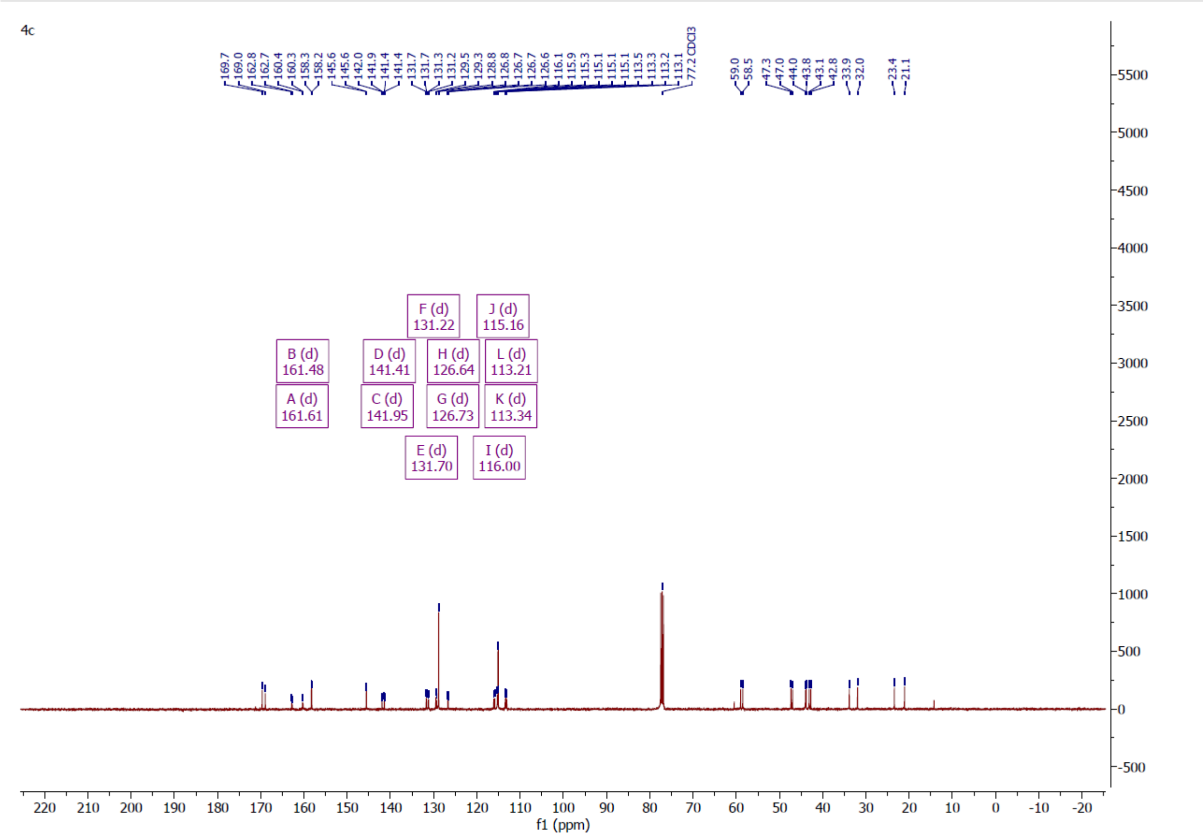

^{1}H NMR spectrum of **4d**

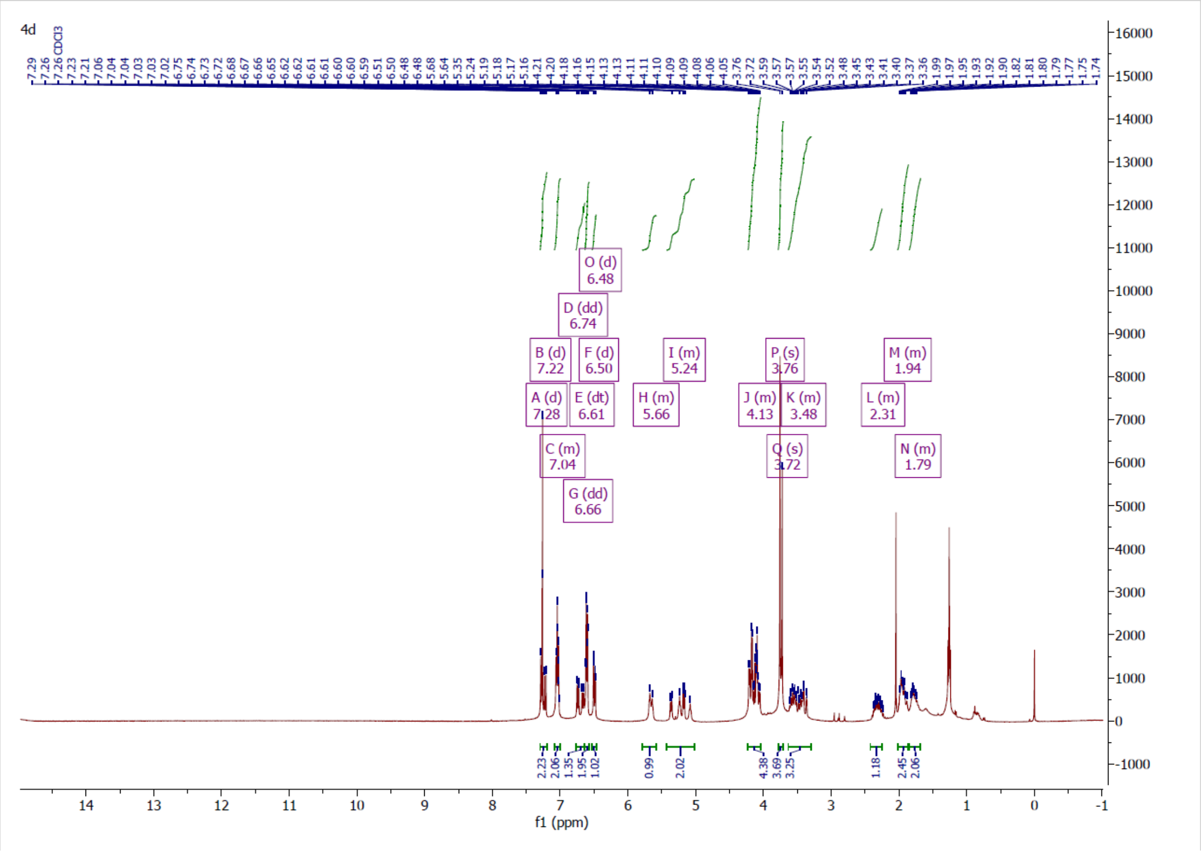

^{13}C NMR spectrum of **4d**

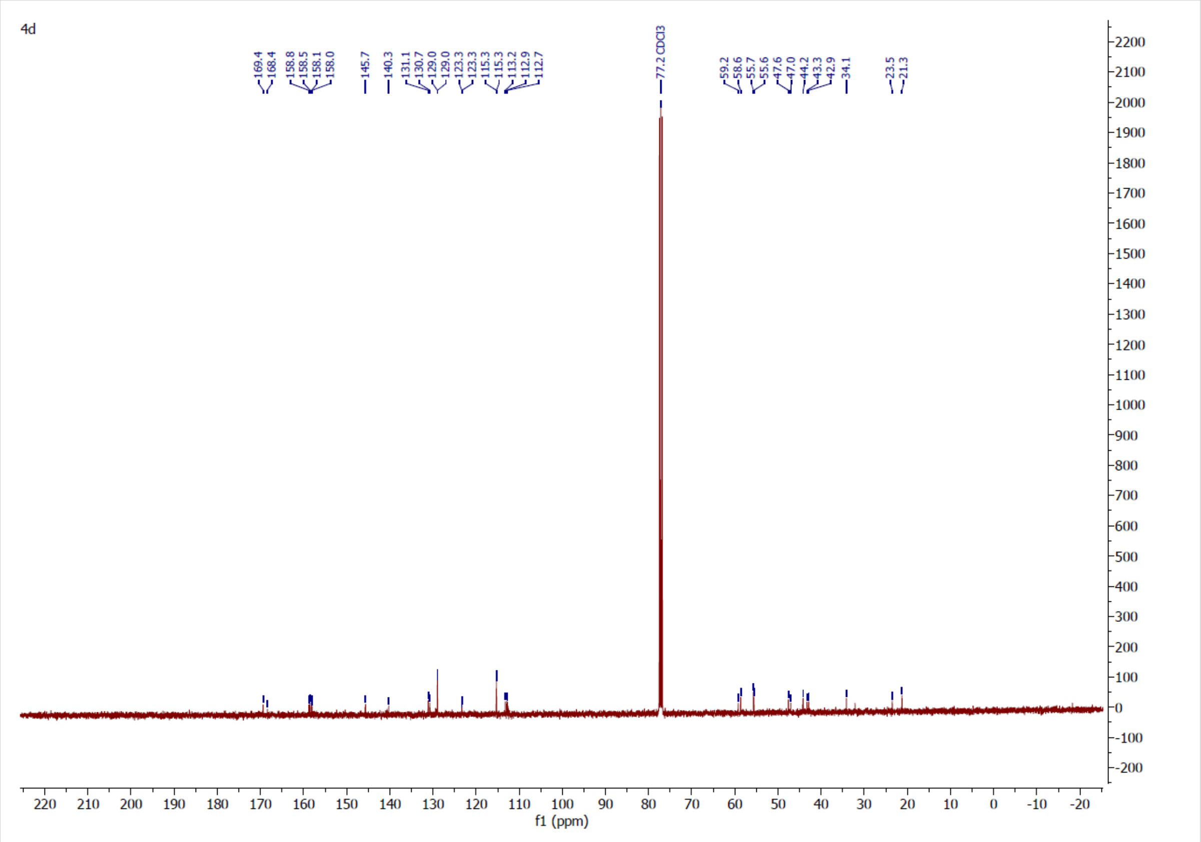

^{1}H NMR spectrum of **12b**

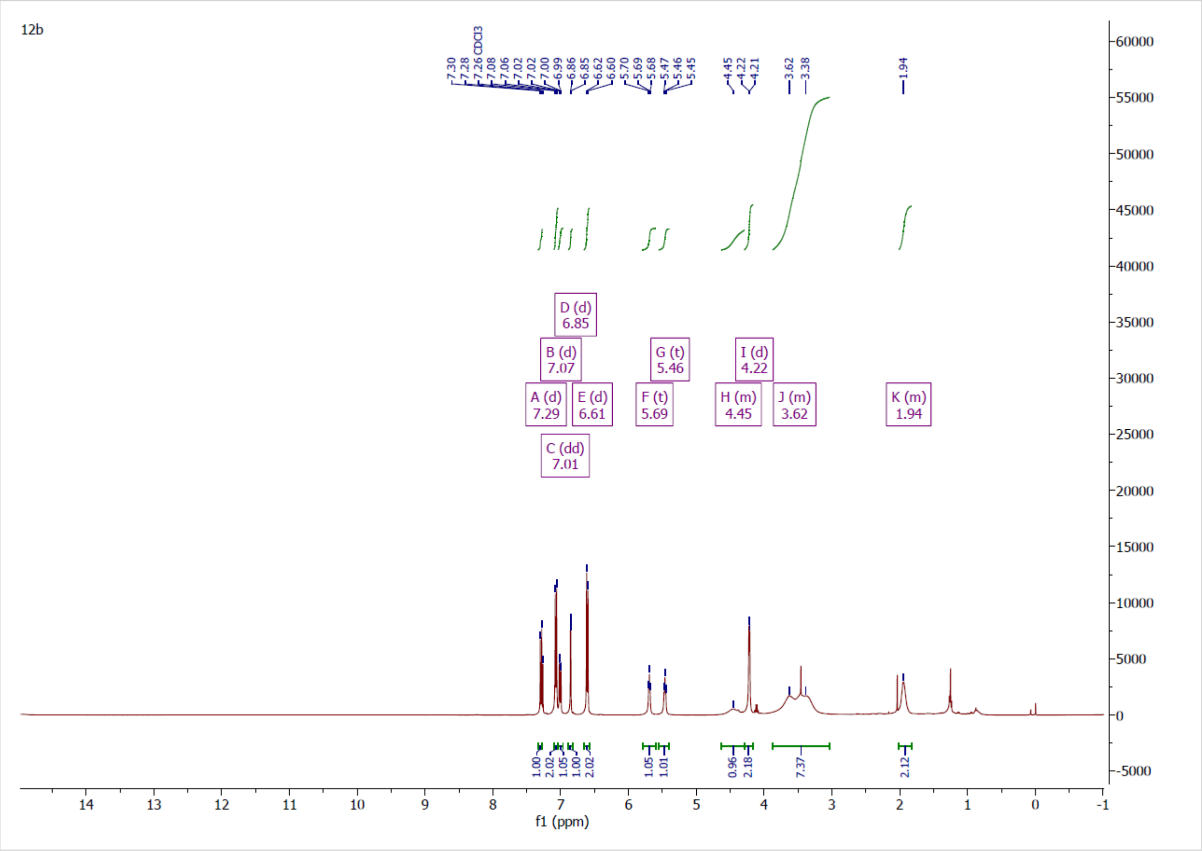

^{13}C NMR spectrum of **12b**

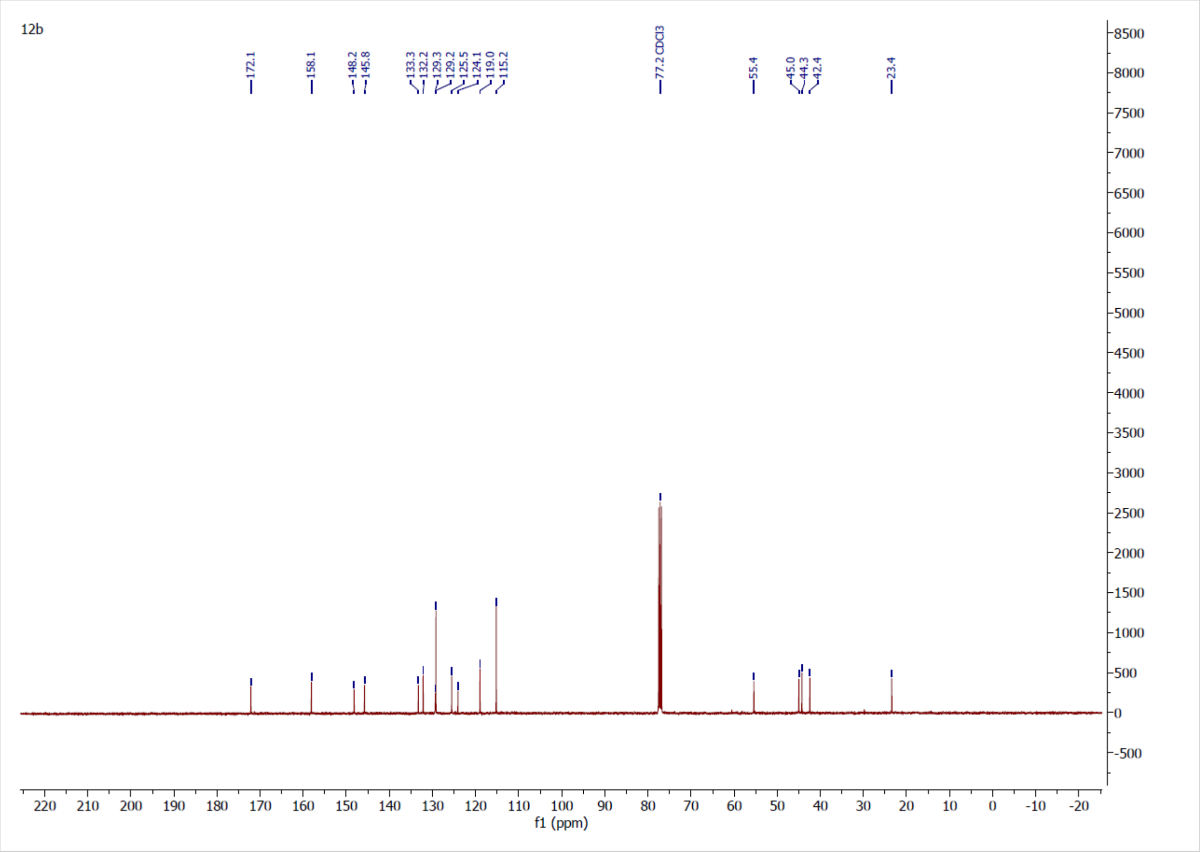

^{1}H NMR spectrum of **12c**

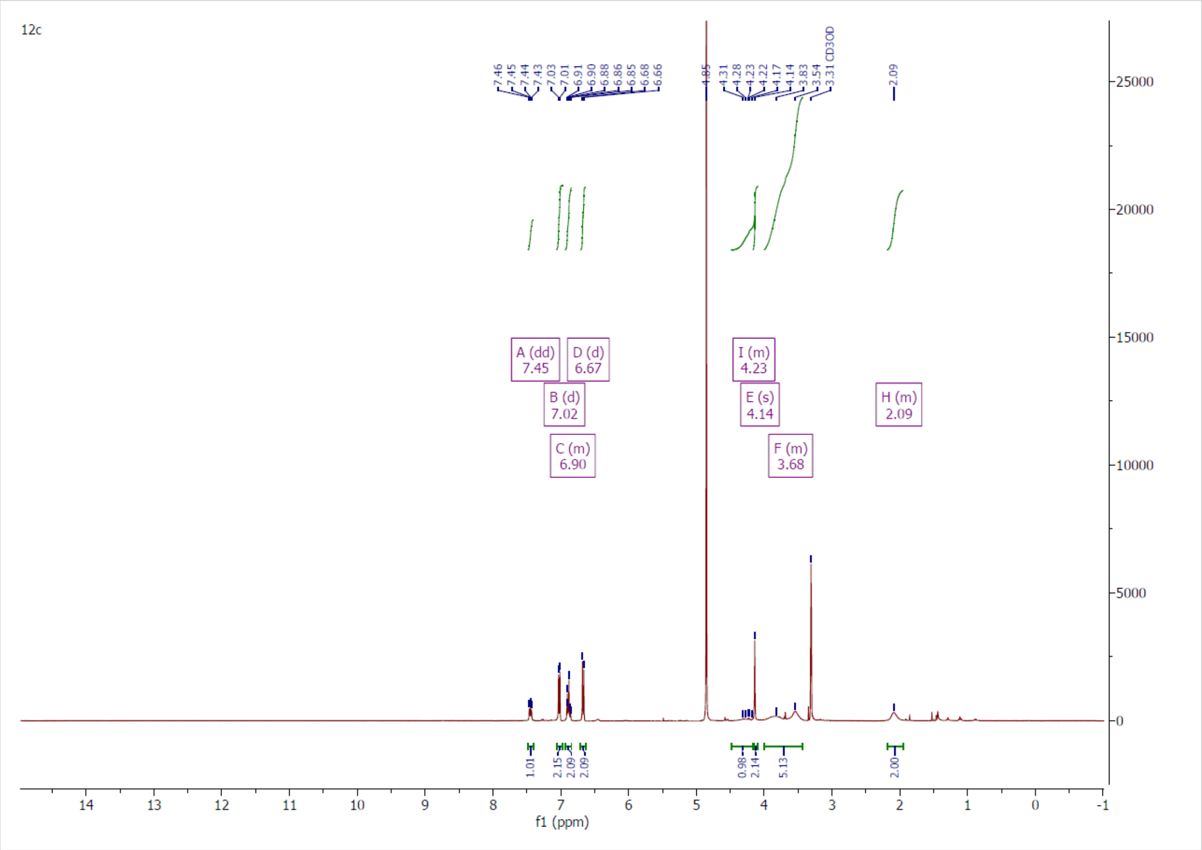

^{13}C NMR spectrum of **12c**

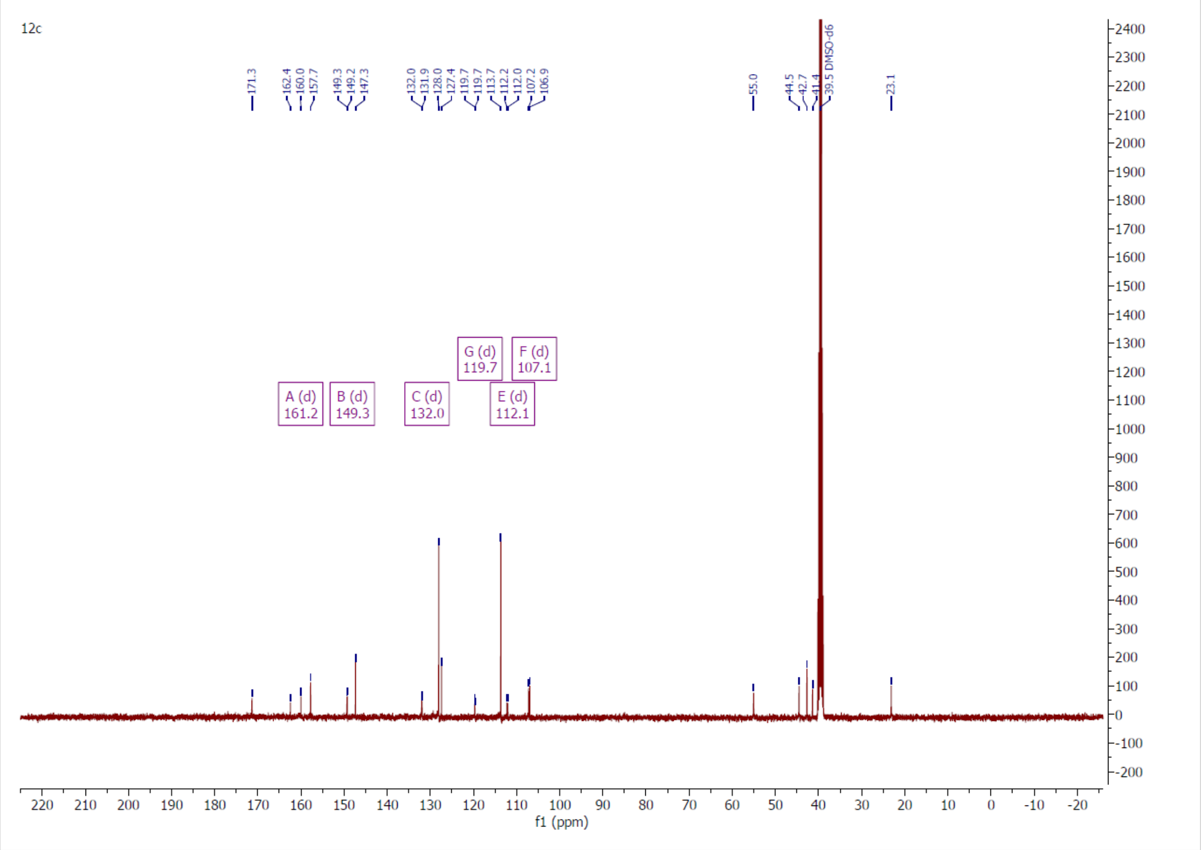

^{1}H NMR spectrum of **12d**

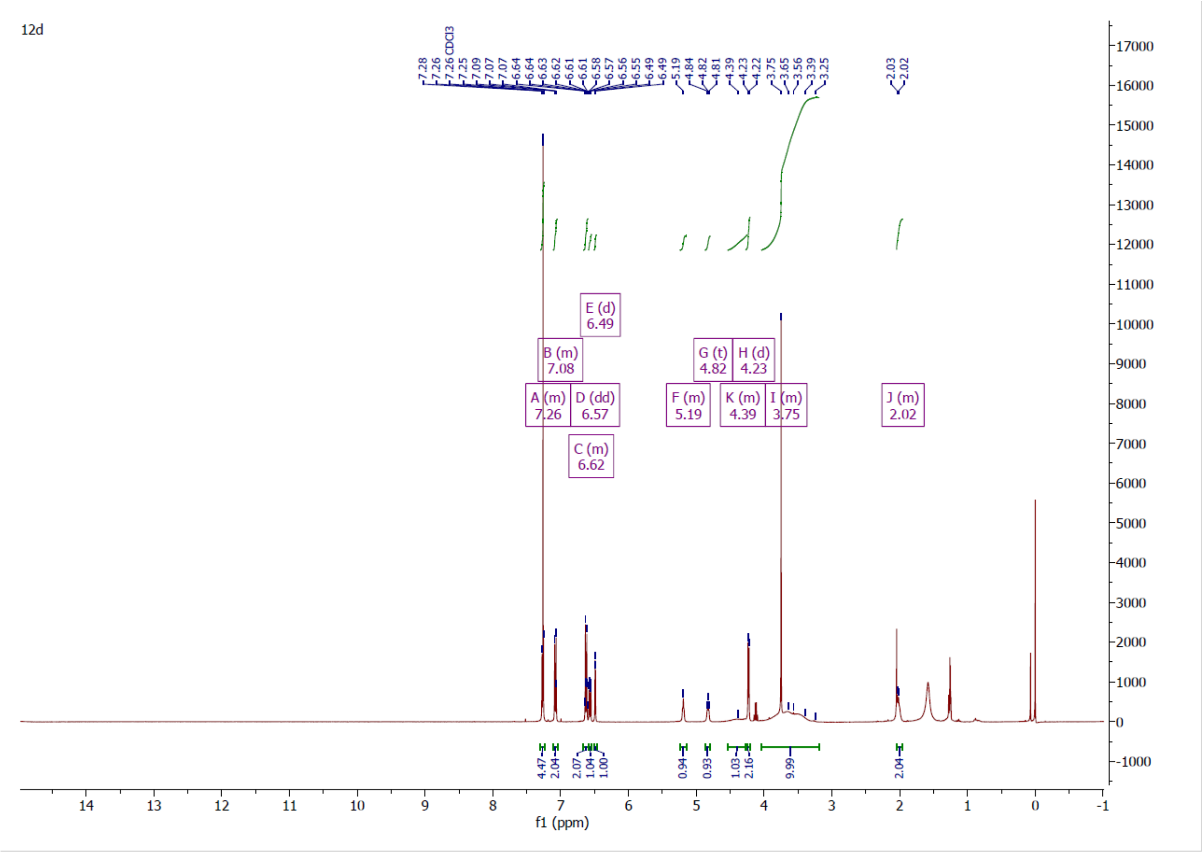

^{13}C NMR spectrum of **12d**

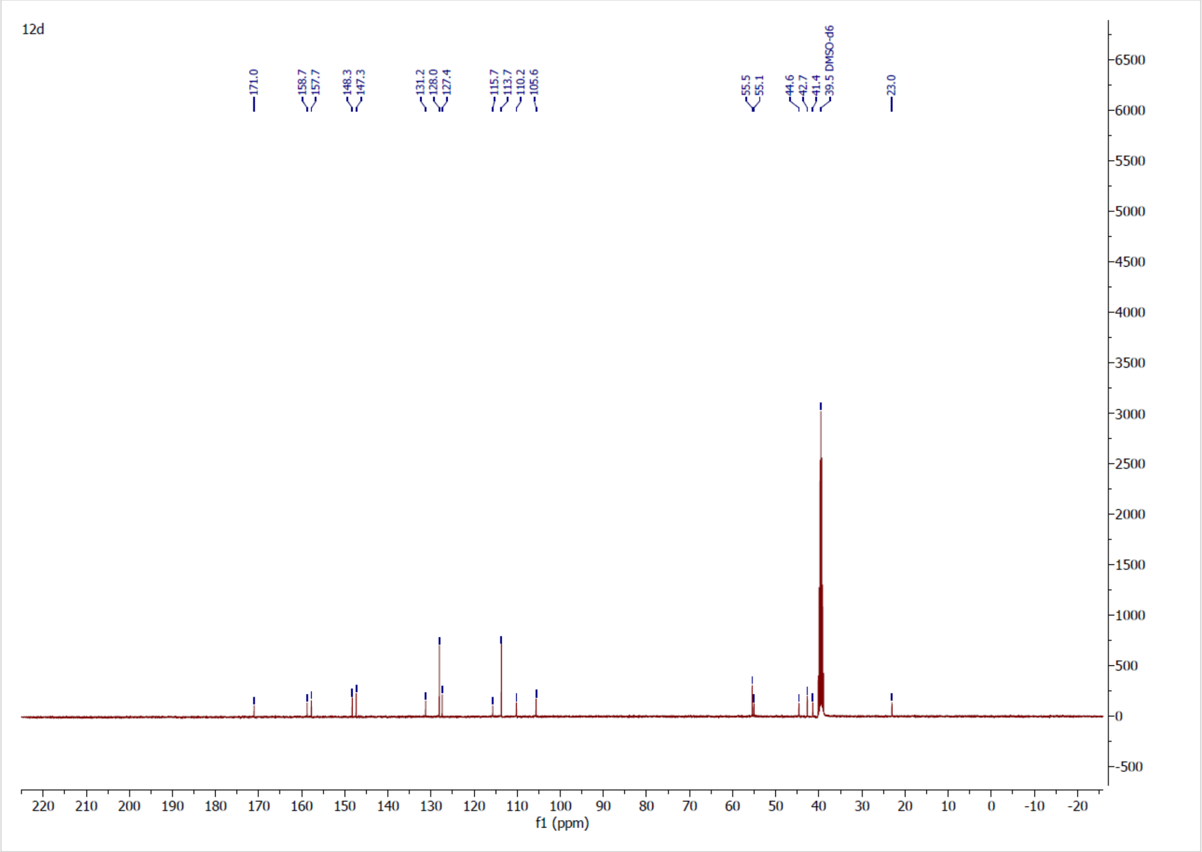

^{1}H NMR spectrum of **13**

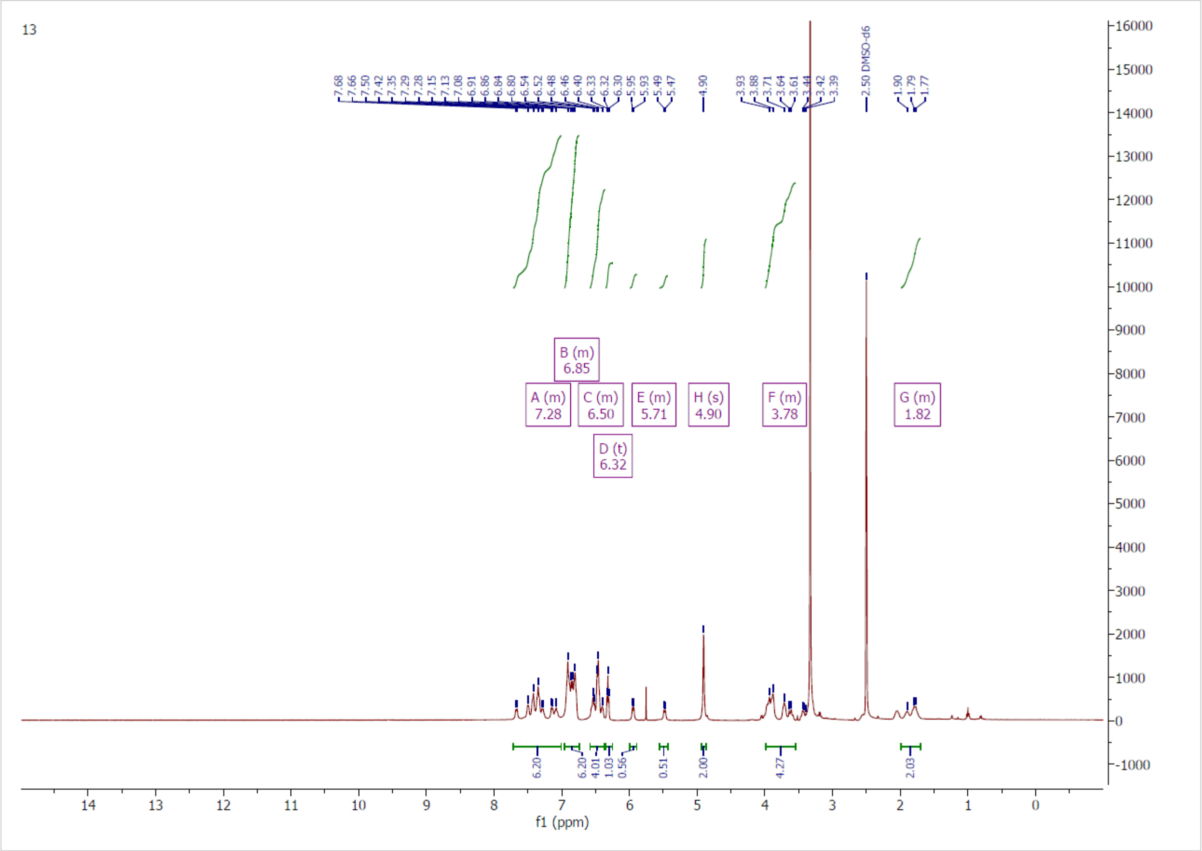

^{13}C NMR spectrum of **13**

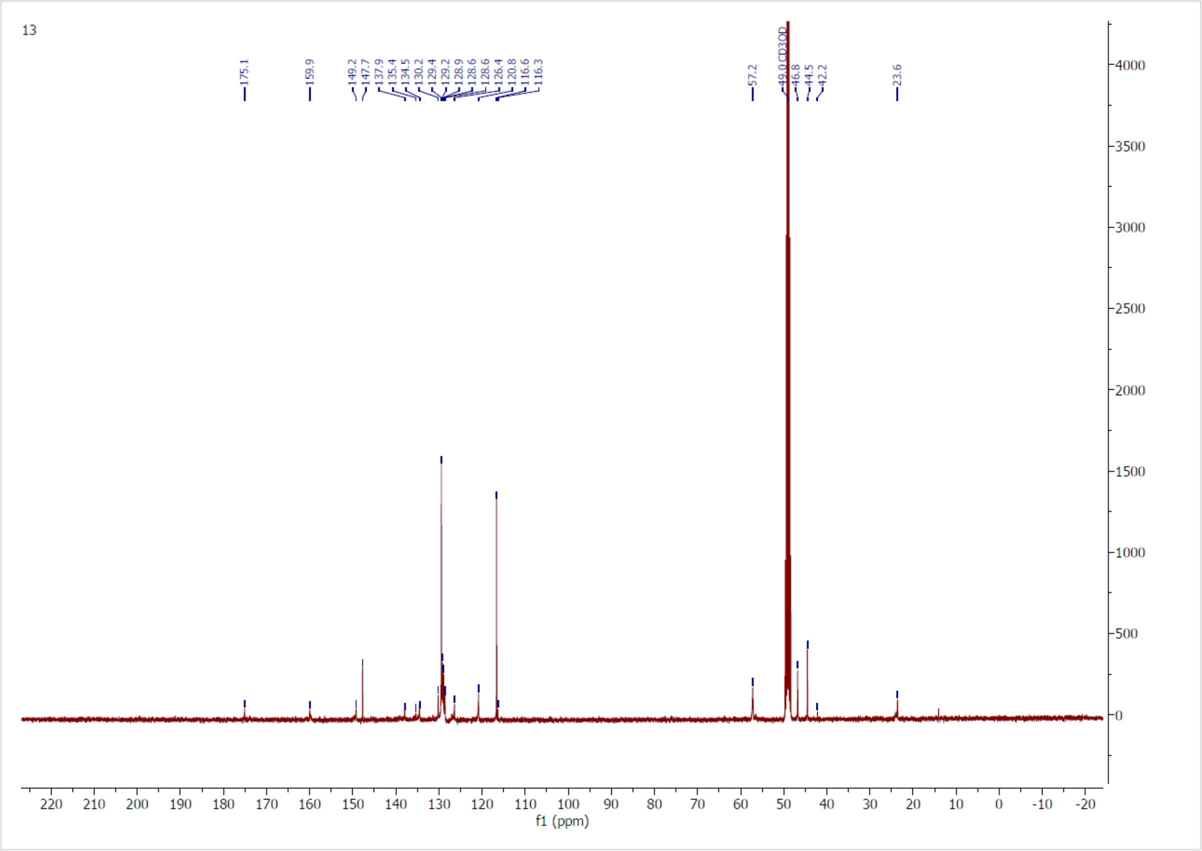

^{1}H NMR spectrum of **18a**

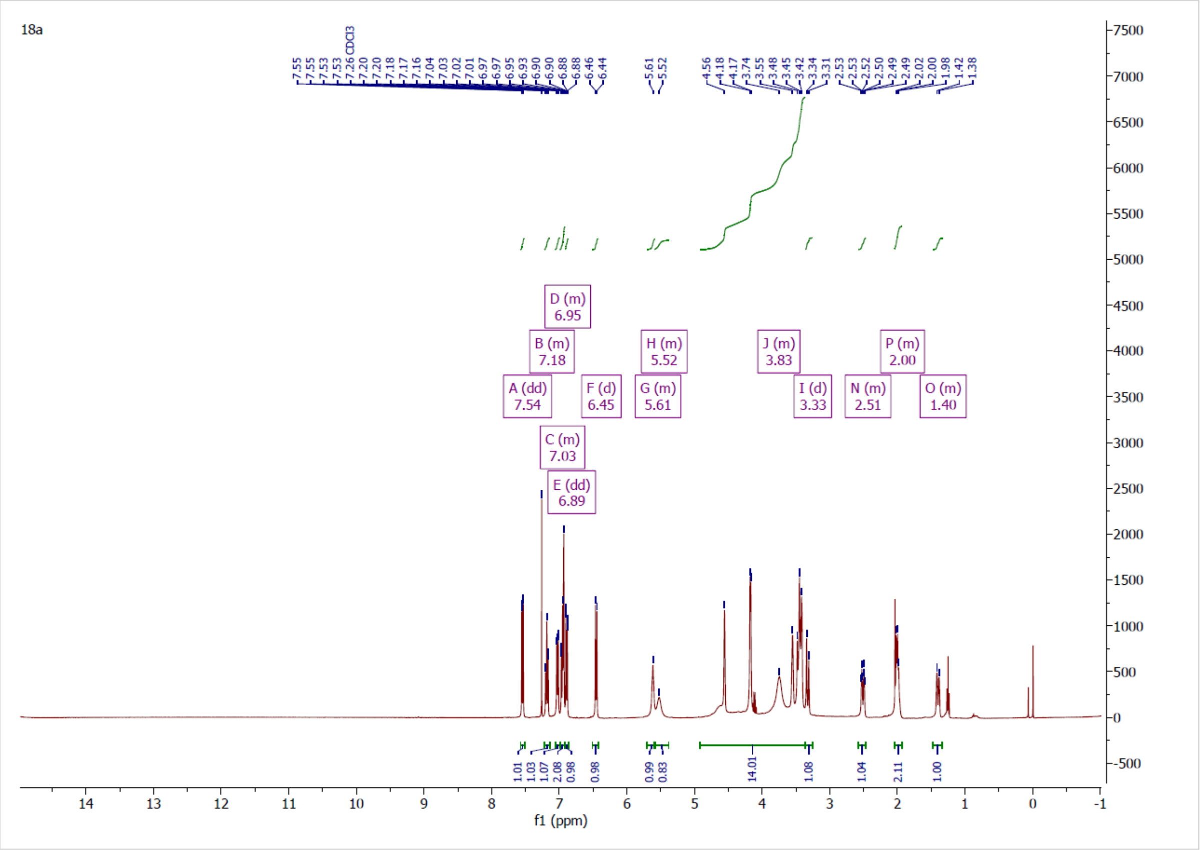

^{13}C NMR spectrum of **18a**

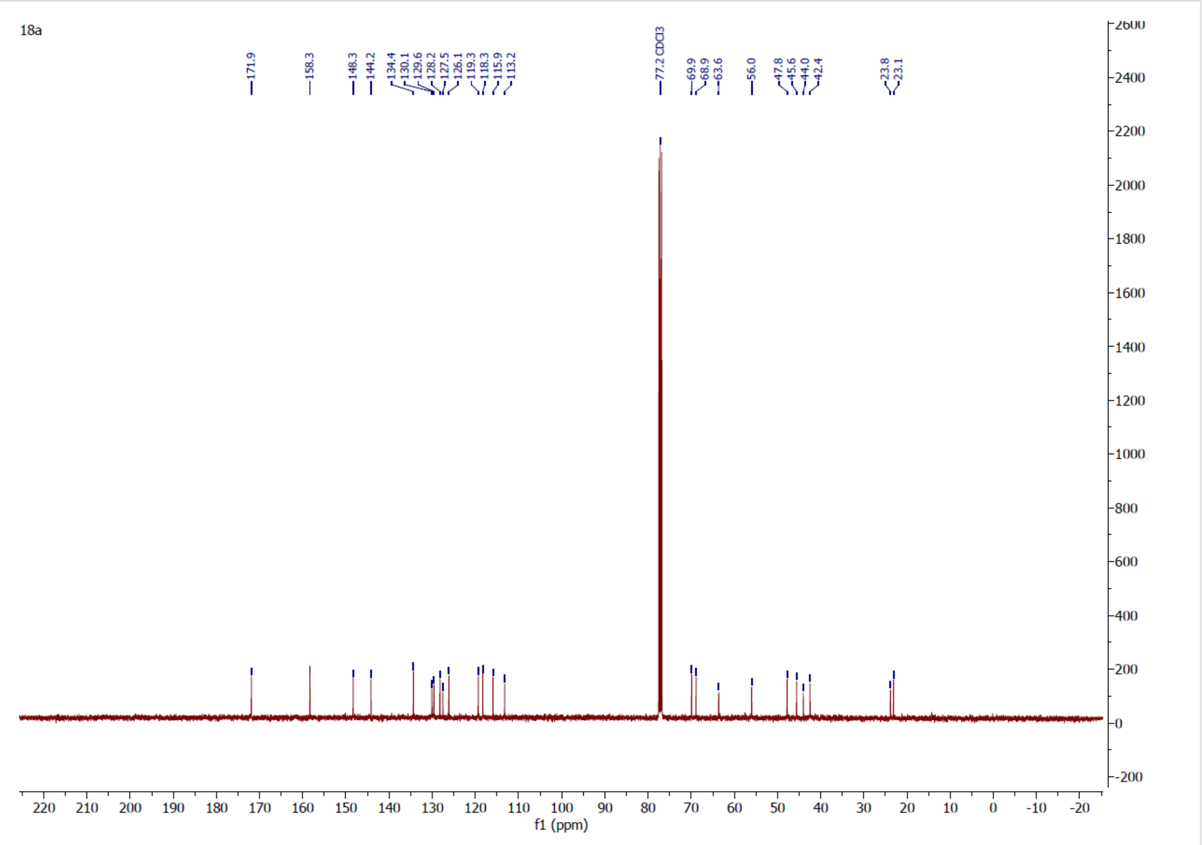

^{1}H NMR spectrum of **18b**

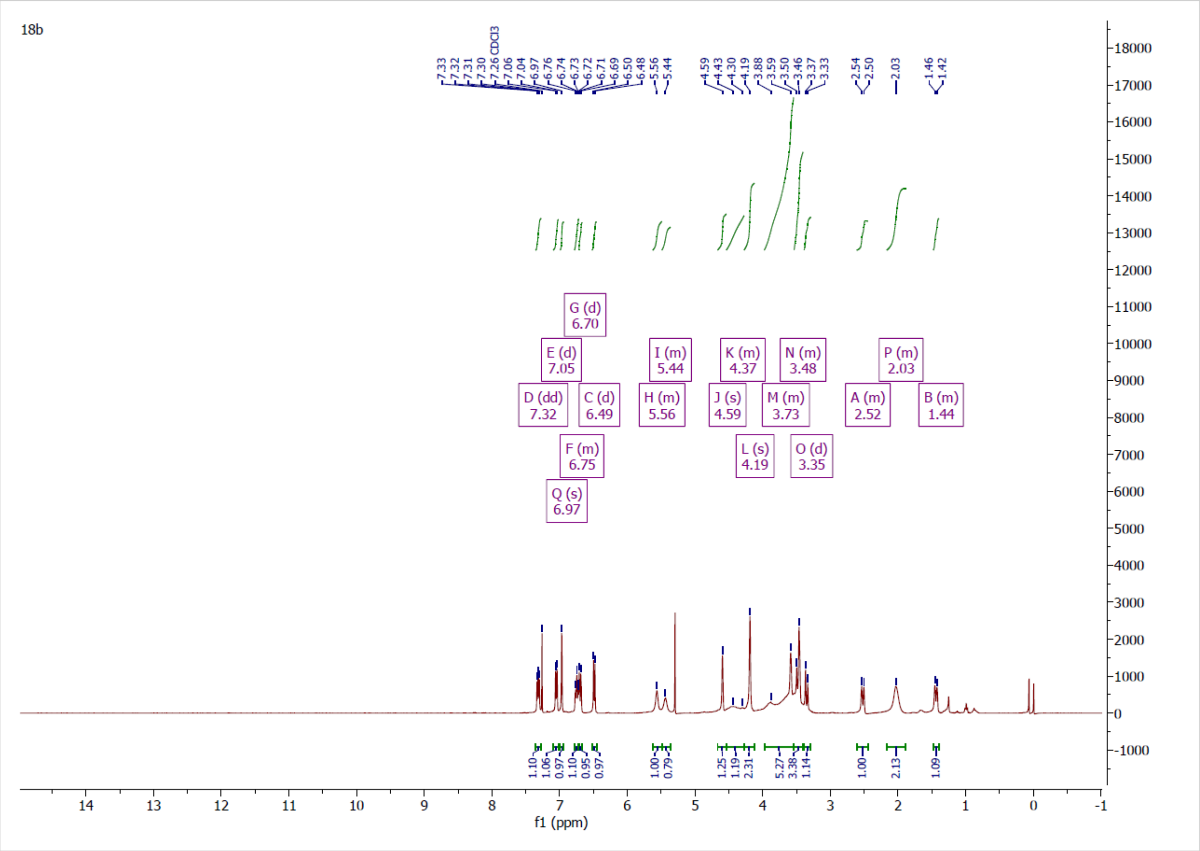

^{13}C NMR spectrum of **18b**

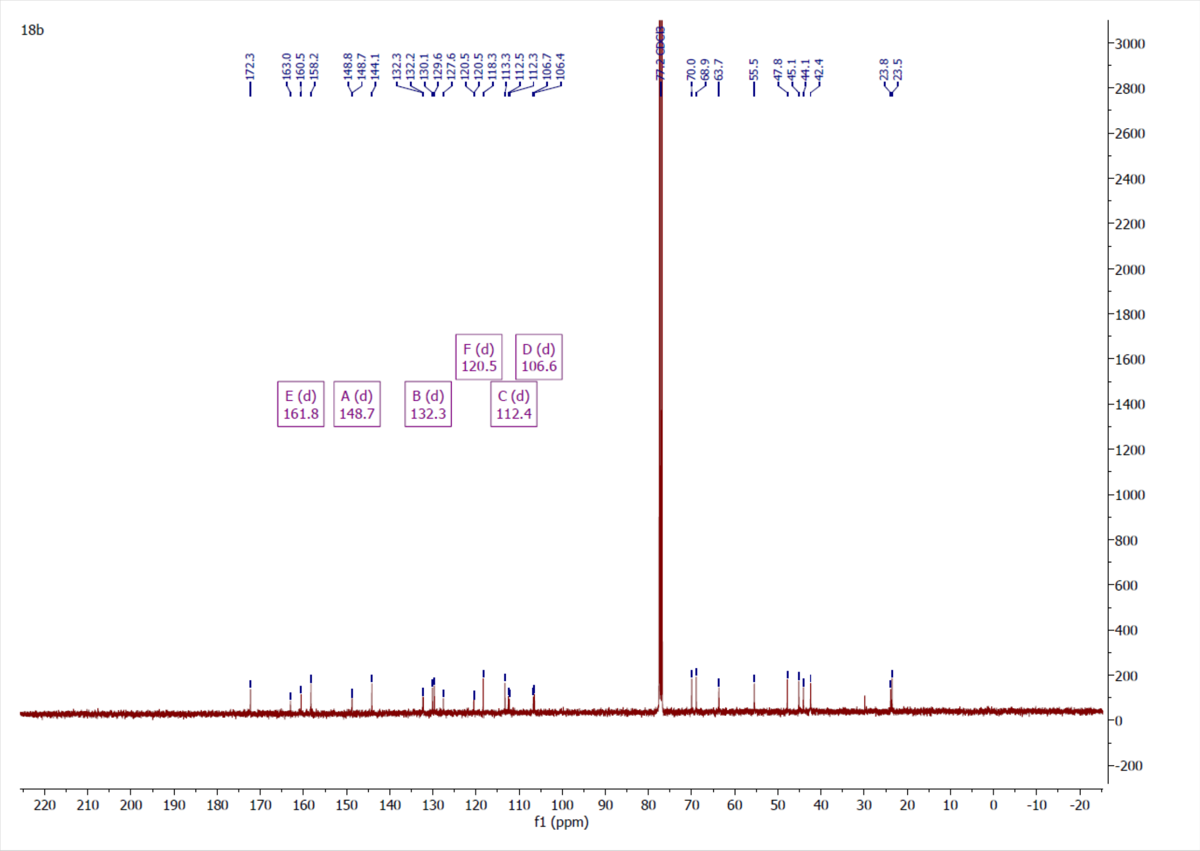

^{1}H NMR of spectrum **18c**

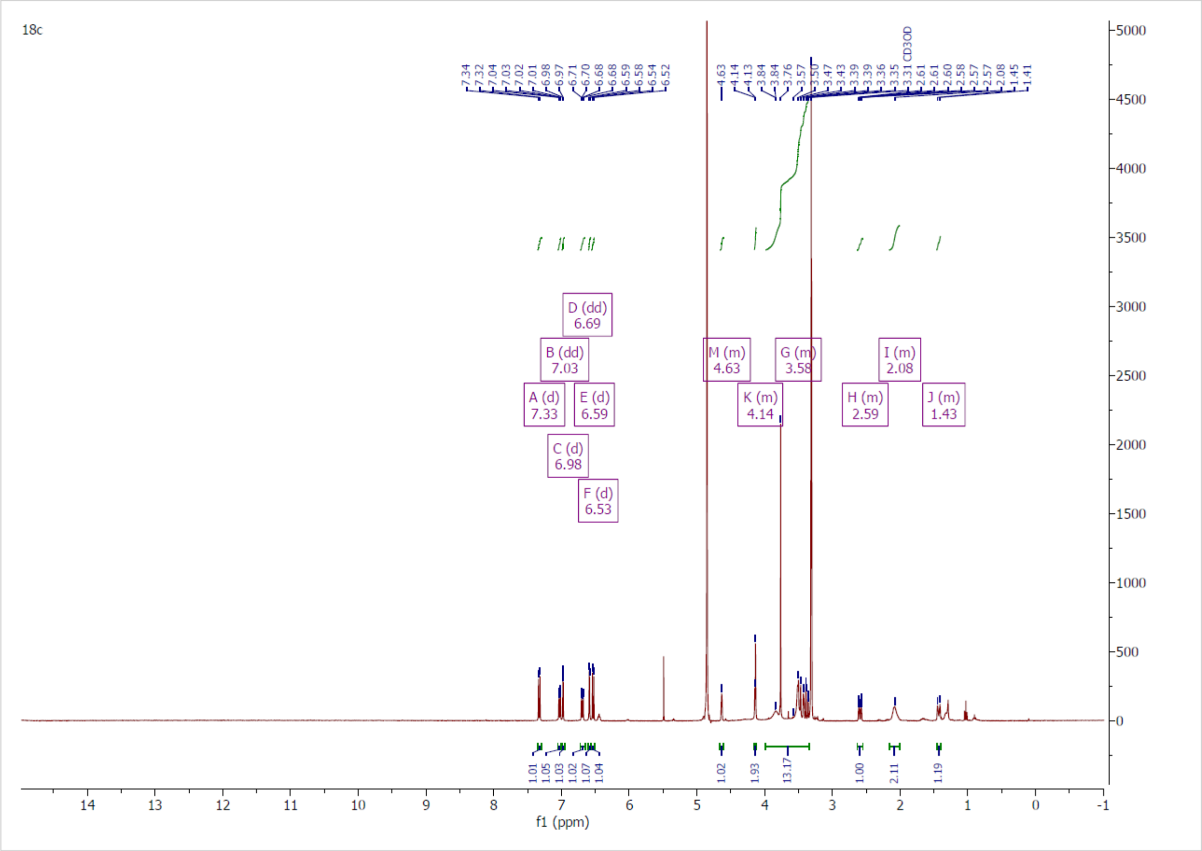

^{13}C NMR of spectrum **18c**

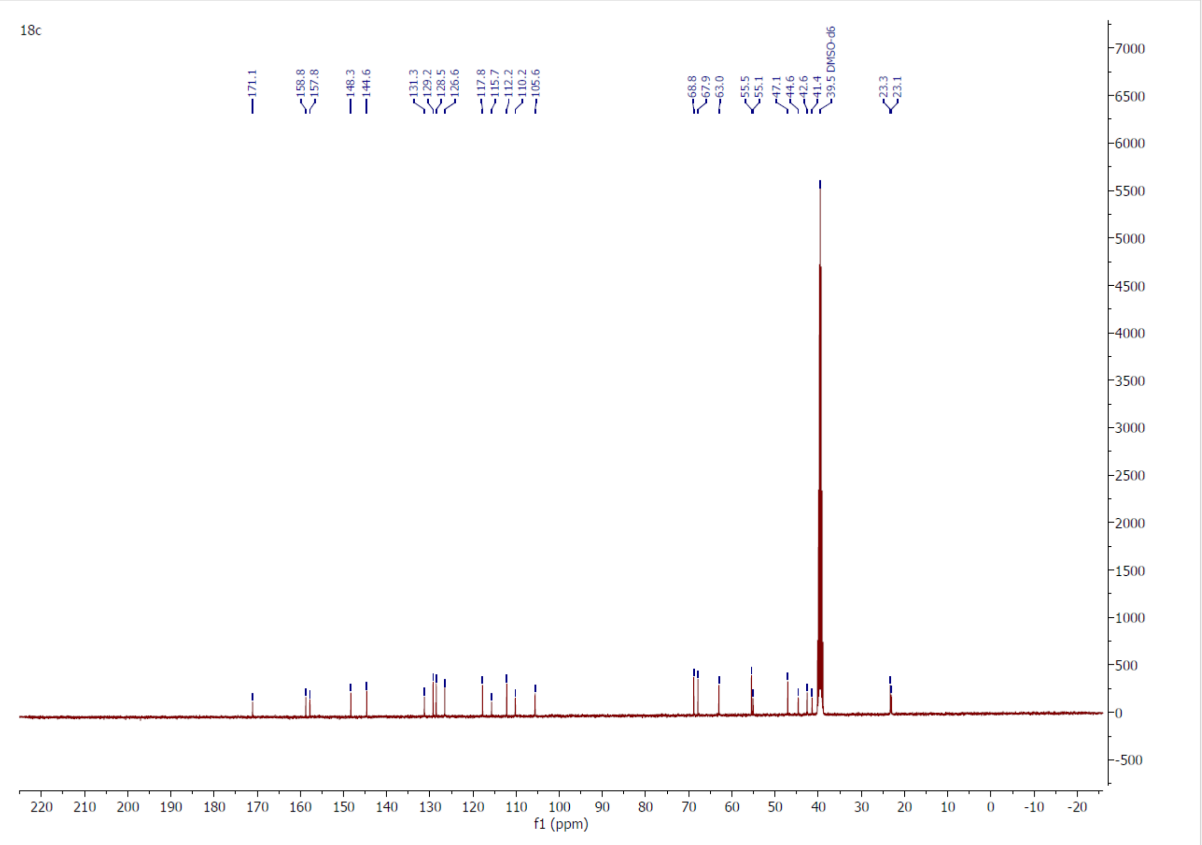

^{1}H NMR of spectrum **18d**

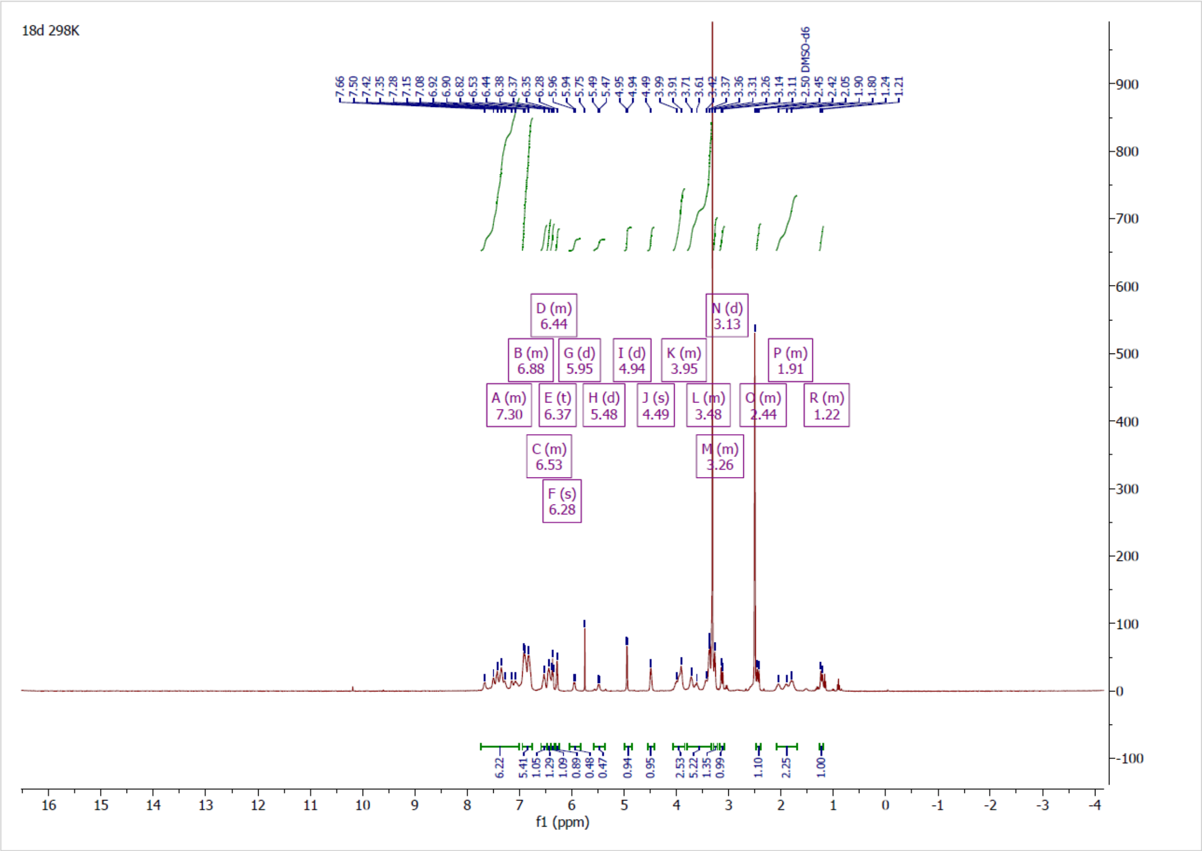

^{1}H NMR spectrum of **18e**

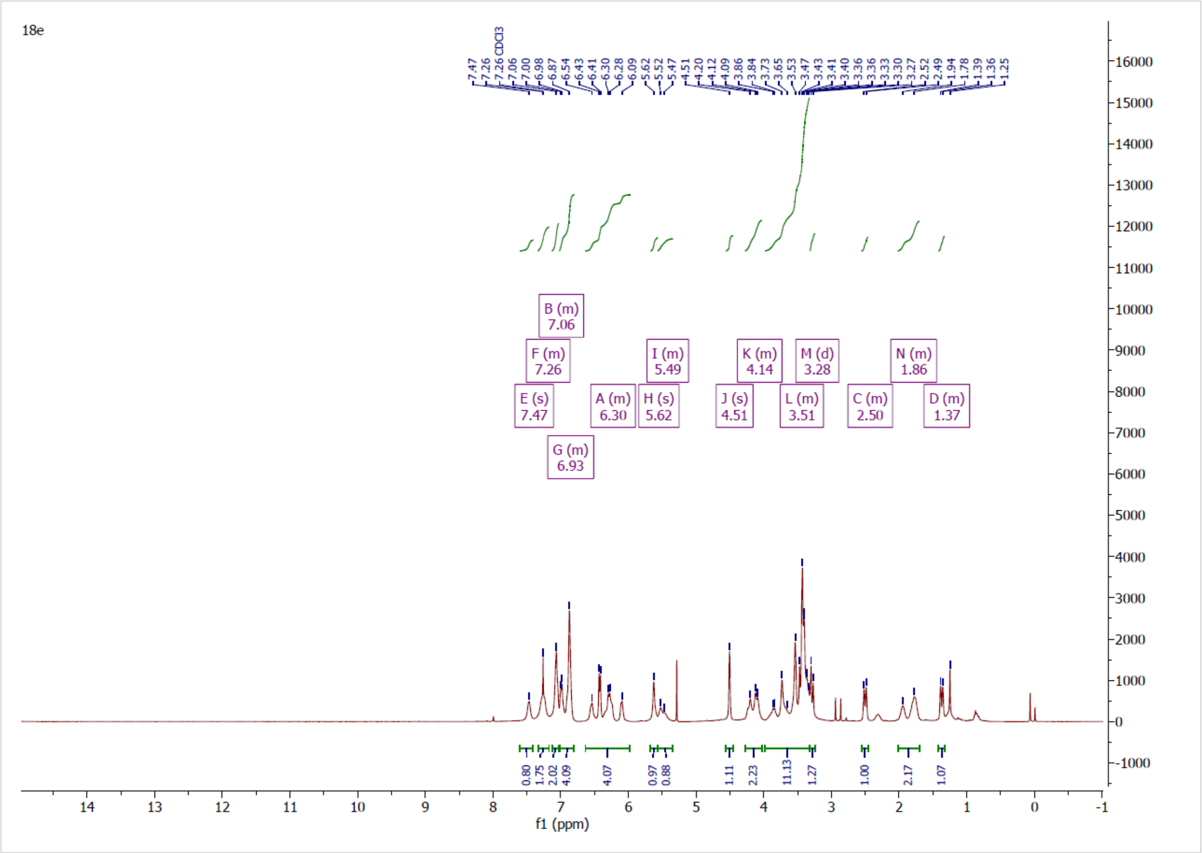

^{1}H NMR spectrum of **18f**

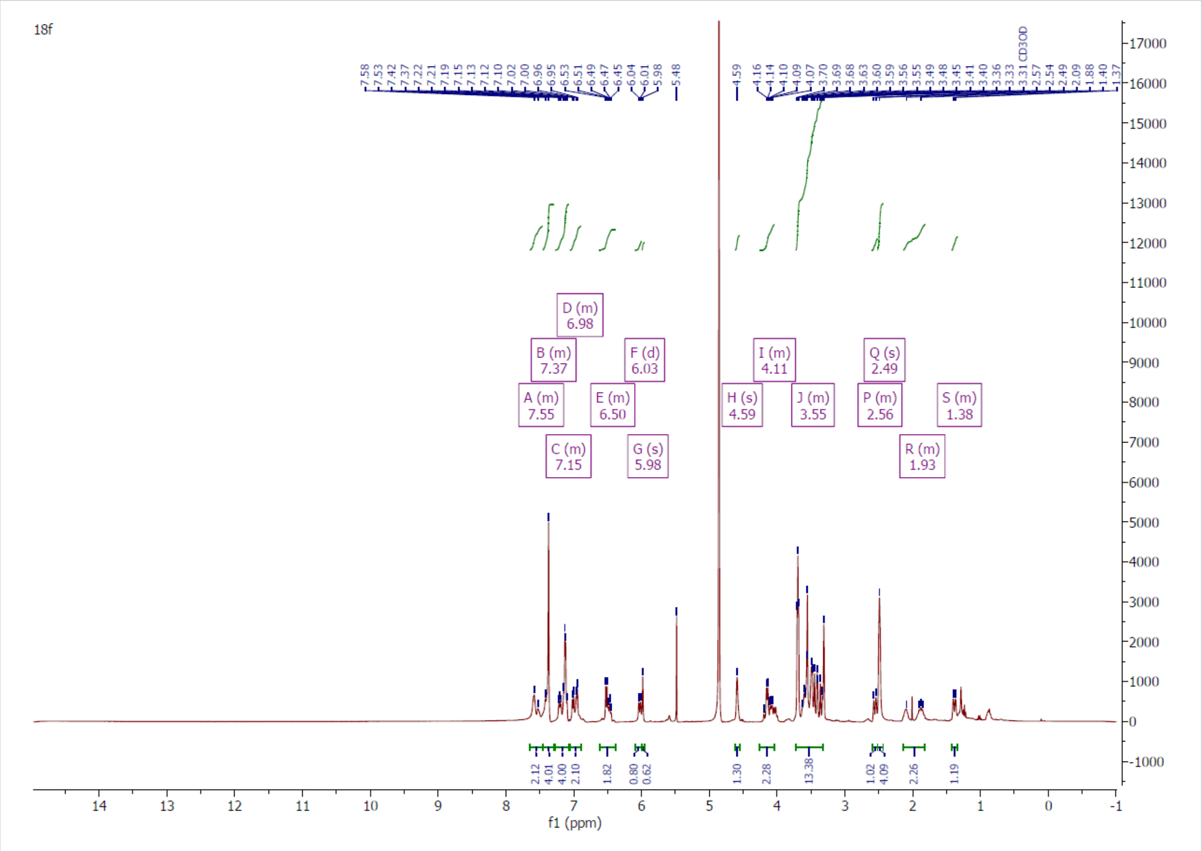

^{13}C NMR spectrum of **18f**

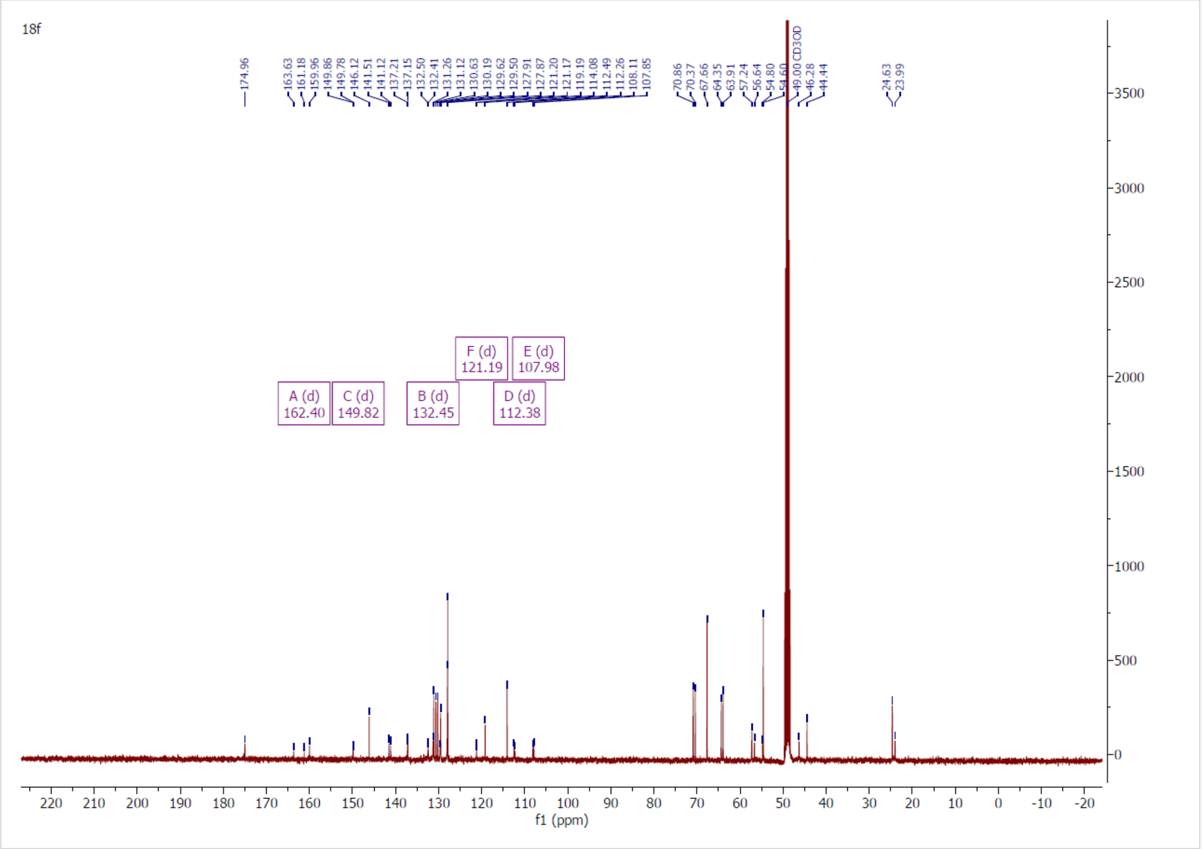

^{1}H NMR spectrum of **19**

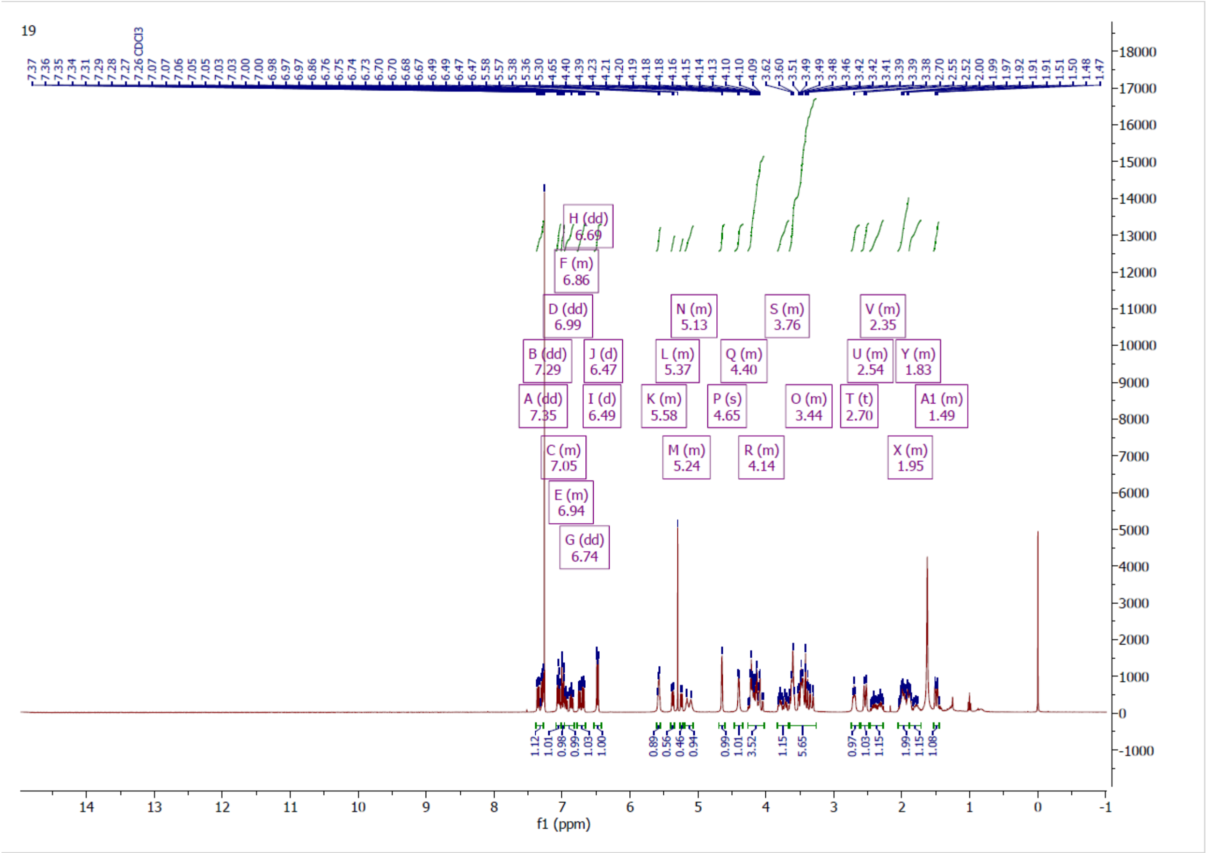

^{13}C NMR spectrum of **19**

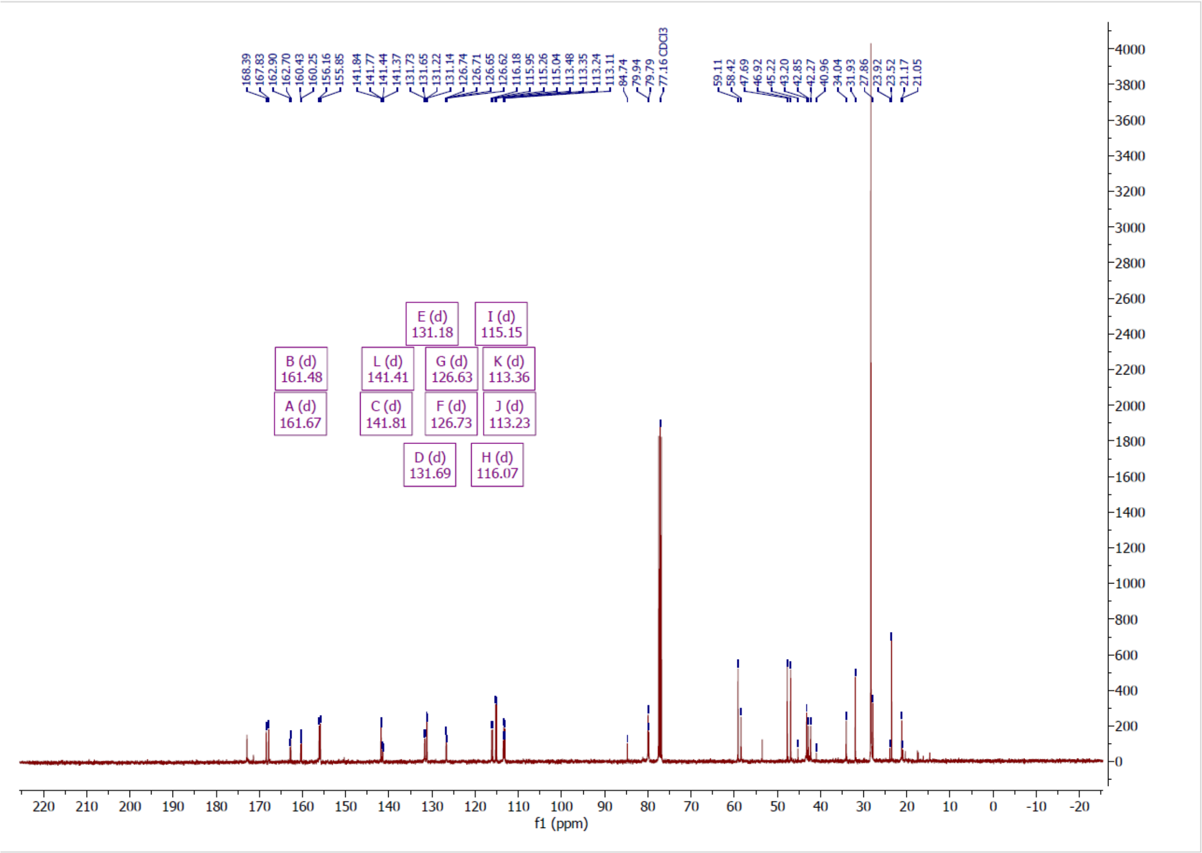

HPLC traces of the samples:

4c

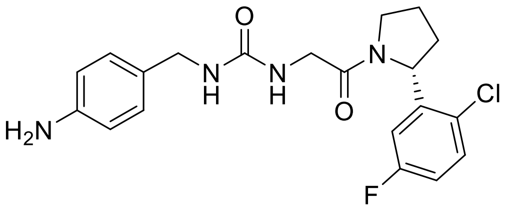

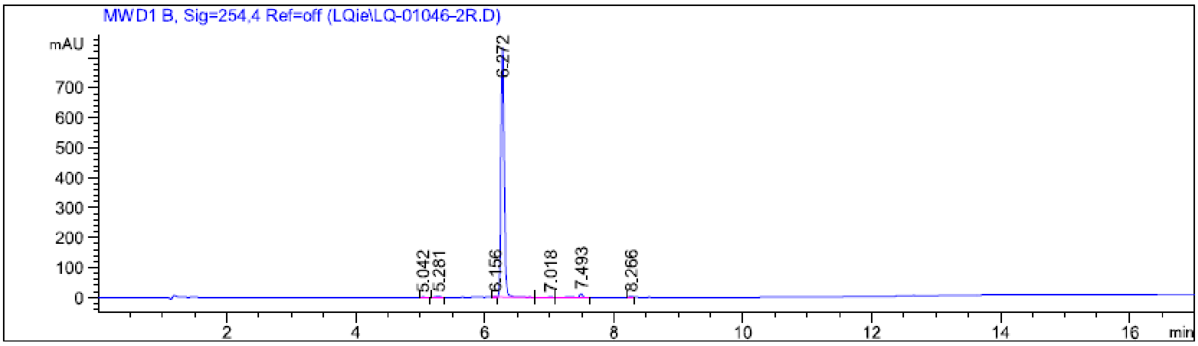

Signal 1: MWD1 B, Sig=254,4 Ref=off

Peak #	RetTime [min]	Type	Width [min]	Area [mAU*s]	Height [mAU]	Area %
1	5.042	VB	0.0489	7.68450	2.43024	0.2651
2	5.281	BB	0.0774	21.82837	4.01667	0.7529
3	6.156	BV E	0.0480	7.35022	2.38483	0.2535
4	6.272	VV R	0.0529	2781.30859	833.11481	95.9366
5	7.018	VB R	0.0657	11.35079	2.56278	0.3915
6	7.493	VB R	0.0684	55.72815	11.94401	1.9222
7	8.266	VV	0.0534	13.86174	4.10016	0.4781

Totals : 2899.11236 860.55350

4d

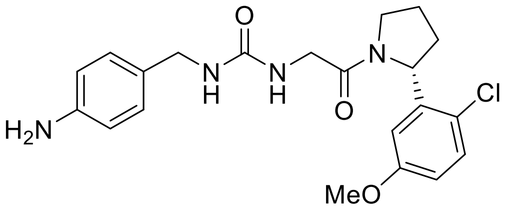

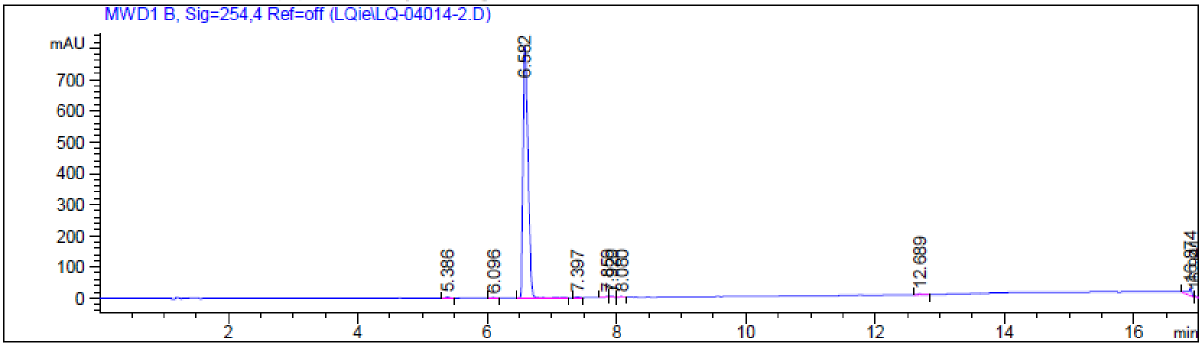

Signal 1: MWD1 B, Sig=254,4 Ref=off

Peak #	RetTime [min]	Type	Width [min]	Area [mAU*s]	Height [mAU]	Area %
1	5.386	BB	0.0542	18.33426	5.32641	0.4102
2	6.096	BB	0.0563	6.46440	1.78260	0.1446
3	6.582	BV R	0.0840	4278.24707	805.39685	95.7182
4	7.397	BB	0.0674	10.61888	2.61569	0.2376
5	7.859	BV	0.0527	13.59439	3.89549	0.3042
6	7.920	VB	0.0565	14.88271	4.08784	0.3330
7	8.080	BV	0.0738	10.82169	2.26417	0.2421
8	12.689	BV R	0.0630	7.94099	1.89044	0.1777
9	16.874	BB	0.0591	98.65726	24.42125	2.2073
10	16.941	BBA	0.0412	10.06746	4.02184	0.2252

Totals : 4469.62909 855.70258

12c

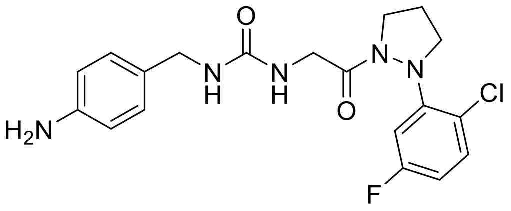

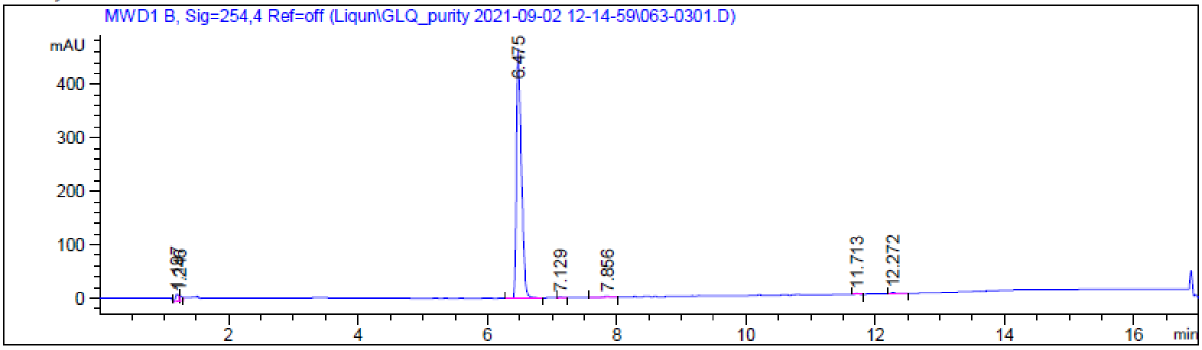

Signal 1: MWD1 B, Sig=254,4 Ref=off

Peak #	RetTime [min]	Type	Width [min]	Area [mAU*s]	Height [mAU]	Area %
1	1.197	BV	0.0566	47.51101	13.01536	1.7679
2	1.246	VV	0.0523	29.69230	9.45481	1.1049
3	6.475	BB	0.0843	2572.36279	466.43826	95.7190
4	7.129	VB	0.0578	6.96782	1.85424	0.2593
5	7.856	VV R	0.0916	12.52124	1.88171	0.4659
6	11.713	BB	0.0574	6.78642	1.91356	0.2525
7	12.272	BV R	0.0671	11.57063	2.64060	0.4305

Totals : 2687.41221 497.19855

12d

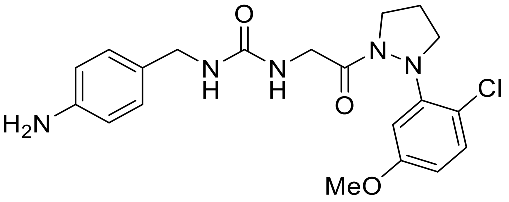

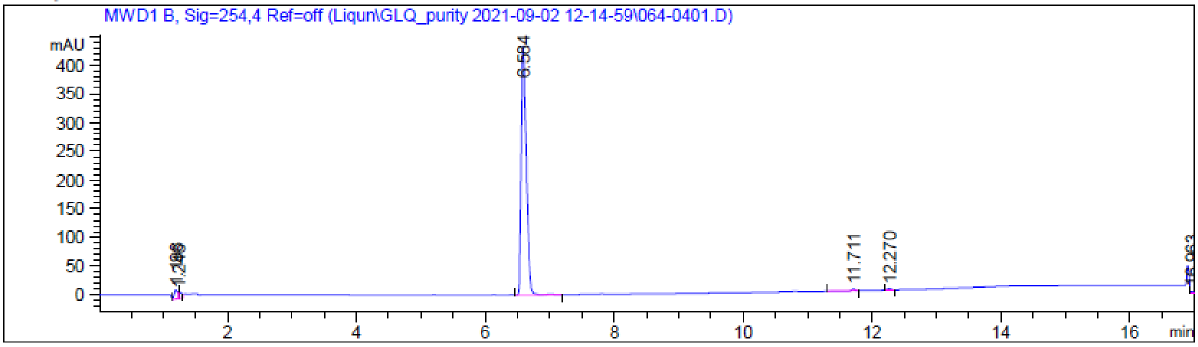

Signal 1: MWD1 B, Sig=254,4 Ref=off

Peak #	RetTime [min]	Type	Width [min]	Area [mAU*s]	Height [mAU]	Area %
1	1.198	BV	0.0560	57.36206	15.21810	2.3117
2	1.246	VV	0.0535	36.44854	11.34896	1.4689
3	6.584	BV R	0.0818	2361.05322	431.58148	95.1492
4	11.711	VB R	0.0615	10.91127	2.79602	0.4397
5	12.270	BB	0.0588	9.43037	2.57129	0.3800
6	16.963	BBA	0.0328	6.21626	3.18646	0.2505
Totals :				2481.42172	466.70231	

13

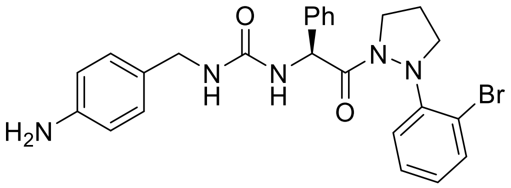

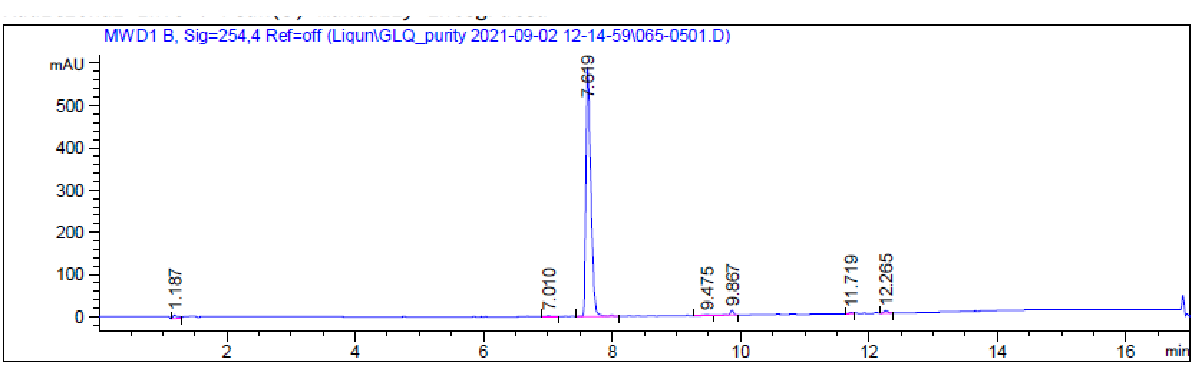


```
=====================================================================
                         Area Percent Report
=====================================================================

Sorted By             :      Signal
Multiplier            :      1.0000
Dilution              :      1.0000
Do not use Multiplier & Dilution Factor with ISTDs


Signal 1: MWD1 B, Sig=254,4 Ref=off
```

Peak #	RetTime [min]	Type	Width [min]	Area [mAU*s]	Height [mAU]	Area %
1	1.187	BV	0.0767	40.02349	7.68677	1.2104
2	7.010	BV R	0.0581	10.60429	2.68114	0.3207
3	7.619	VV R	0.0784	3159.53052	589.00824	95.5538
4	9.475	BV E	0.0879	12.03453	1.89886	0.3640
5	9.867	VB R	0.0573	49.51830	11.49602	1.4976
6	11.719	BV	0.0585	10.13576	2.65926	0.3065
7	12.265	VB	0.0681	24.69955	5.53118	0.7470

Totals : 3306.54643 620.96147

Chiral HPLC trace of **13**

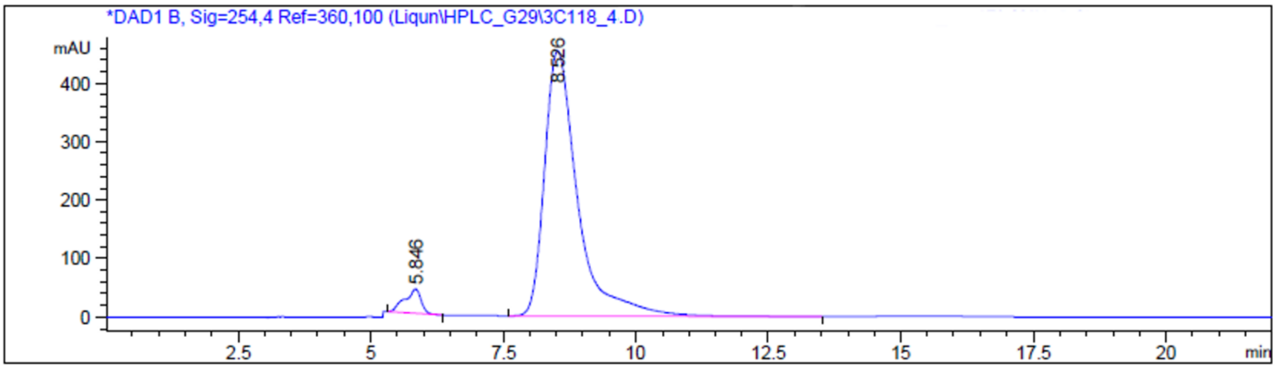

Signal 1: DAD1 B, Sig=254,4 Ref=360,100

Peak #	RetTime [min]	Type	Width [min]	Area [mAU*s]	Height [mAU]	Area %
1	5.846	BB	0.2988	881.04022	41.31134	4.2779
2	8.526	BB	0.6537	1.97140e4	452.98358	95.7221

Totals : 2.05951e4 494.29492

18a

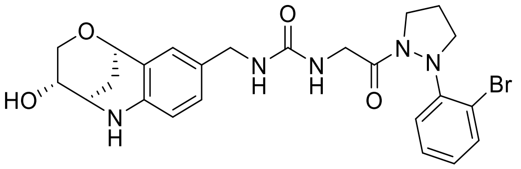

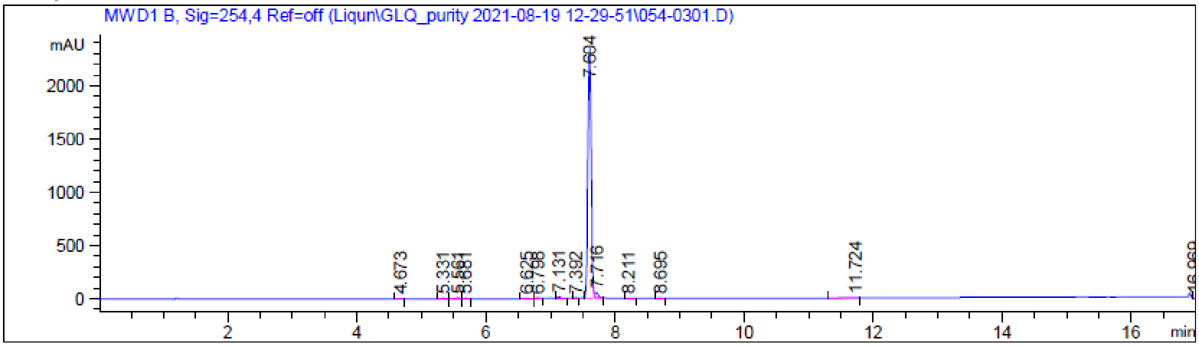

Signal 1: MWD1 B, Sig=254,4 Ref=off

Peak #	RetTime [min]	Type	Width [min]	Area [mAU*s]	Height [mAU]	Area %
1	4.673	BV	0.0518	6.92639	2.13823	0.0827
2	5.331	VB	0.0584	19.91087	5.48421	0.2376
3	5.561	BV	0.0526	30.44770	9.20338	0.3634
4	5.681	VB	0.0604	13.21370	3.32065	0.1577
5	6.625	VB	0.0668	17.72252	3.77524	0.2115
6	6.798	BB	0.0543	24.39424	7.05751	0.2911
7	7.131	BB	0.0530	50.98883	15.25528	0.6085
8	7.392	VB	0.0566	14.76780	4.04772	0.1762
9	7.604	BV R	0.0536	7971.99707	2351.15063	95.1434
10	7.716	VB E	0.0537	179.94830	50.32281	2.1476
11	8.211	VB	0.0676	18.23977	3.96664	0.2177
12	8.695	VV	0.0624	16.78795	4.04521	0.2004
13	11.724	VB R	0.0635	9.06476	2.13547	0.1082
14	16.969	BBA	0.0301	4.51685	2.62129	0.0539

Totals : 8378.92676 2464.52427

18b

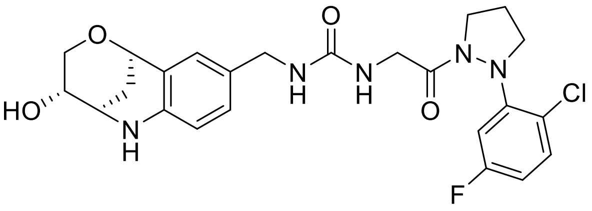

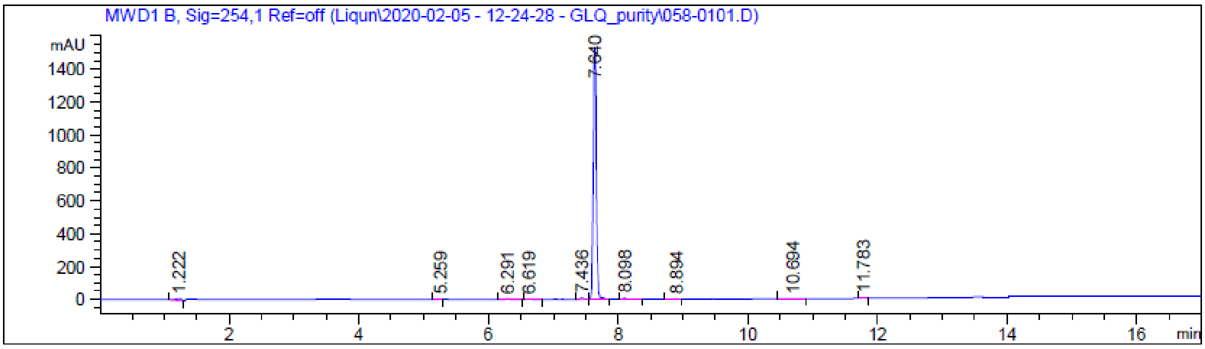

Signal 1: MWD1 B, Sig=254,1 Ref=off

Peak #	RetTime [min]	Type	Width [min]	Area [mAU*s]	Height [mAU]	Area %
1	1.222	BB	0.0853	77.32372	12.65040	1.4448
2	5.259	BV	0.0558	12.13067	3.38373	0.2267
3	6.291	BV R	0.0644	20.92699	4.84511	0.3910
4	6.619	BV R	0.0749	19.82815	3.92620	0.3705
5	7.436	BB	0.0583	36.53005	9.20137	0.6826
6	7.640	BV R	0.0507	5118.95996	1544.28174	95.6459
7	8.098	BV R	0.0577	25.63019	6.84403	0.4789
8	8.894	VB R	0.0783	11.02600	2.00135	0.2060
9	10.694	VV R	0.0744	9.06303	1.80984	0.1693
10	11.783	BB	0.0549	20.57088	5.87435	0.3844
Totals :				5351.98963	1594.81812	

18c

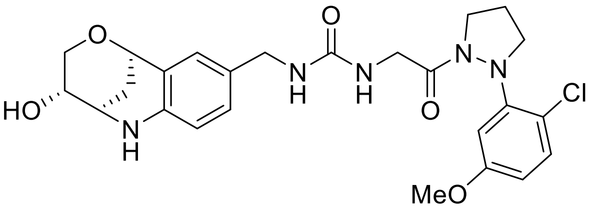

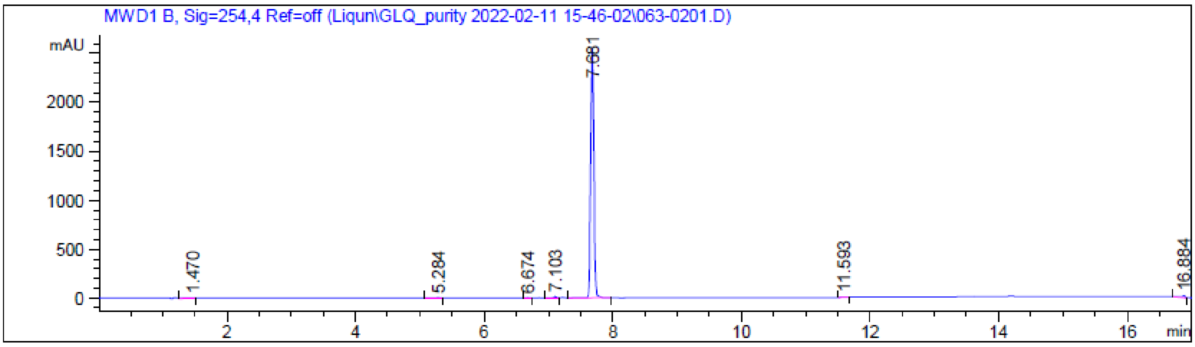


```
=====================================================================
                          Area Percent Report
=====================================================================

Sorted By             :      Signal
Multiplier            :      1.0000
Dilution              :      1.0000
Do not use Multiplier & Dilution Factor with ISTDs


Signal 1: MWD1 B, Sig=254,4 Ref=off

Peak RetTime Type  Width     Area      Height     Area
  #   [min]        [min]   [mAU*s]     [mAU]       %
----|-------|----|-------|----------|----------|--------|
   1   1.470 VB    0.1836   47.49691    3.65423   0.5380
   2   5.284 VV R  0.0484   16.09598    5.15570   0.1823
   3   6.674 BB    0.0515    8.59070    2.67022   0.0973
   4   7.103 VV R  0.0535   42.76931   12.00964   0.4844
   5   7.681 VB R  0.0529 8593.93945 2572.66626  97.3412
   6  11.593 BB    0.0571   12.70692    3.43655   0.1439
   7  16.884 BB    0.0645  107.07703   22.92338   1.2128

Totals :                  8828.67631 2622.51597
```

18d

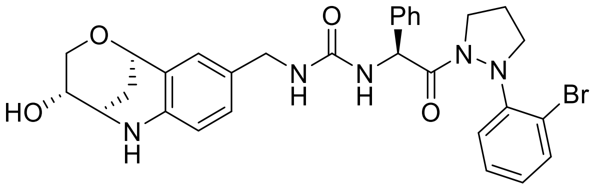

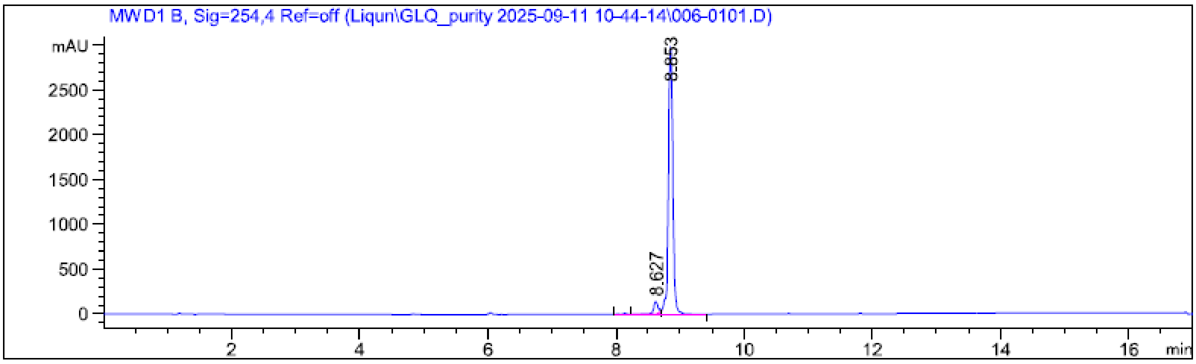

Signal 1: MWD1 B, Sig=254,4 Ref=off

Peak #	RetTime [min]	Type	Width [min]	Area [mAU*s]	Height [mAU]	Area %
1	8.627	VV E	0.0751	709.64941	140.01707	4.6742
2	8.853	VV R	0.0768	1.44726e4	2959.89893	95.3258

Totals : 1.51823e4 3099.91600

18e

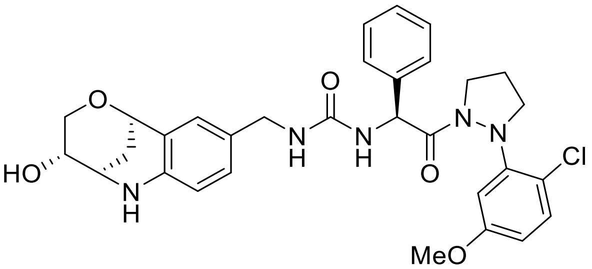

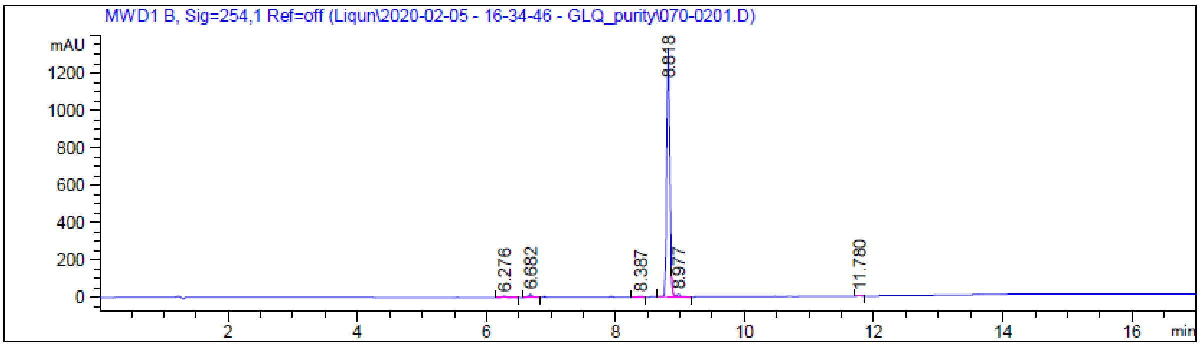

Signal 1: MWD1 B, Sig=254,1 Ref=off

Peak #	RetTime [min]	Type	Width [min]	Area [mAU*s]	Height [mAU]	Area %
1	6.276	BV R	0.0613	27.01065	6.65642	0.5648
2	6.682	BV	0.0635	64.87892	15.28597	1.3567
3	8.387	BB	0.0591	9.43747	2.55260	0.1974
4	8.818	VV R	0.0543	4611.80664	1333.21375	96.4399
5	8.977	VB E	0.0665	60.61702	13.47251	1.2676
6	11.780	BB	0.0553	8.30364	2.34369	0.1736
Totals :				4782.05435	1373.52493	

18f

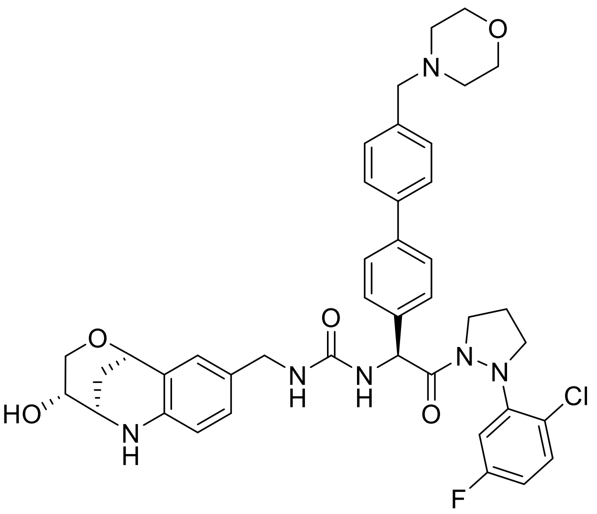

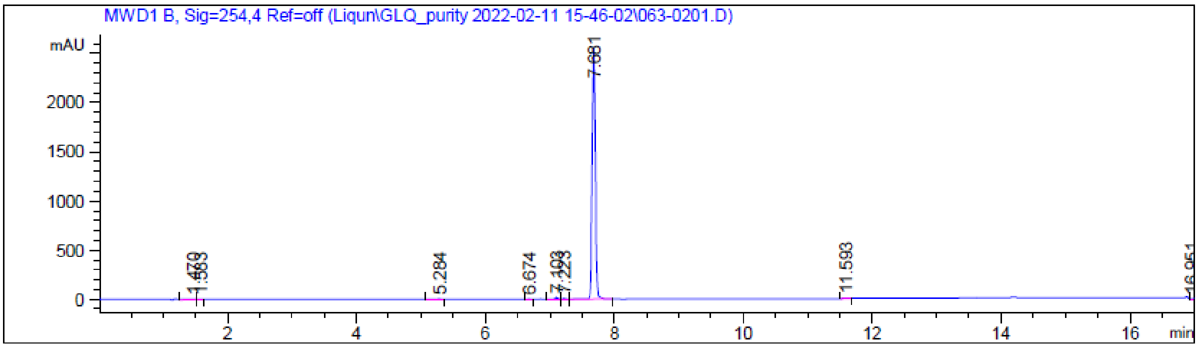


```
=====================================================================
                         Area Percent Report
=====================================================================

Sorted By             :      Signal
Multiplier            :      1.0000
Dilution              :      1.0000
Do not use Multiplier & Dilution Factor with ISTDs


Signal 1: MWD1 B, Sig=254,4 Ref=off

Peak RetTime Type  Width     Area      Height     Area
  #   [min]        [min]   [mAU*s]     [mAU]        %
----|-------|----|-------|----------|----------|--------|
   1   1.470 VB    0.1836   47.49691    3.65423   0.5421
   2   1.583 BV    0.0854   10.88764    1.94244   0.1243
   3   5.284 VV R  0.0484   16.09598    5.15570   0.1837
   4   6.674 BB    0.0515    8.59070    2.67022   0.0981
   5   7.103 VV R  0.0535   42.76931   12.00964   0.4882
   6   7.223 VB    0.0506   20.69253    6.25897   0.2362
   7   7.681 VB R  0.0529 8593.93945 2572.66626  98.0882
   8  11.593 BB    0.0571   12.70692    3.43655   0.1450
   9  16.951 BBA   0.0353    8.26479    3.80878   0.0943

Totals :                  8761.44424 2611.60278
```

19

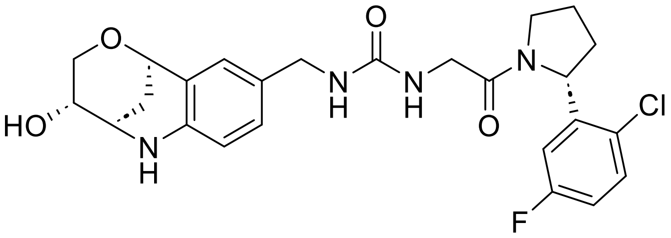

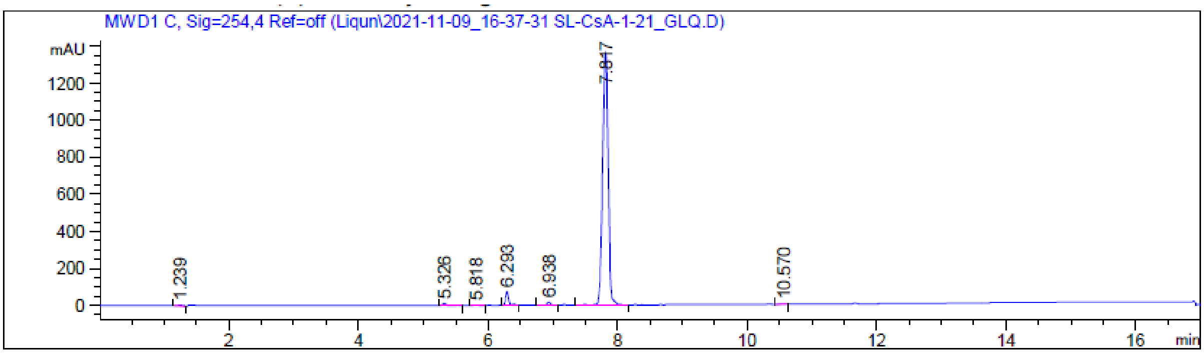


```
=====================================================================
                      Area Percent Report
=====================================================================

Sorted By             :      Signal
Multiplier            :      1.0000
Dilution              :      1.0000
Do not use Multiplier & Dilution Factor with ISTDs


Signal 1: MWD1 C, Sig=254,4 Ref=off
```

Peak #	RetTime [min]	Type	Width [min]	Area [mAU*s]	Height [mAU]	Area %
1	1.239	BB	0.1036	29.98028	3.82044	0.3604
2	5.326	BV R	0.0516	36.88723	10.87157	0.4434
3	5.818	BV R	0.0630	9.40400	2.33504	0.1130
4	6.293	BV R	0.0514	254.30585	73.69482	3.0568
5	6.938	BB	0.0536	64.96711	18.22521	0.7809
6	7.817	VB R	0.0899	7912.43896	1358.41040	95.1089
7	10.570	BV	0.0764	11.36061	2.19498	0.1366

Totals : 8319.34405 1469.55247

Chiral HPLC trace for (*R*)-2-(2-chloro-5-methoxyphenyl)pyrrolidine hydrochloride (provided by LCC):

```
Method Info      : JP00312305, OD-H hexane:iPrOH(85:15), 1.0 mL/min

Sample Info      : SS04700702, IC-3, hex:iPA (80:20), 0.5 ml/min

Additional Info : Peak(s) manually integrated
```

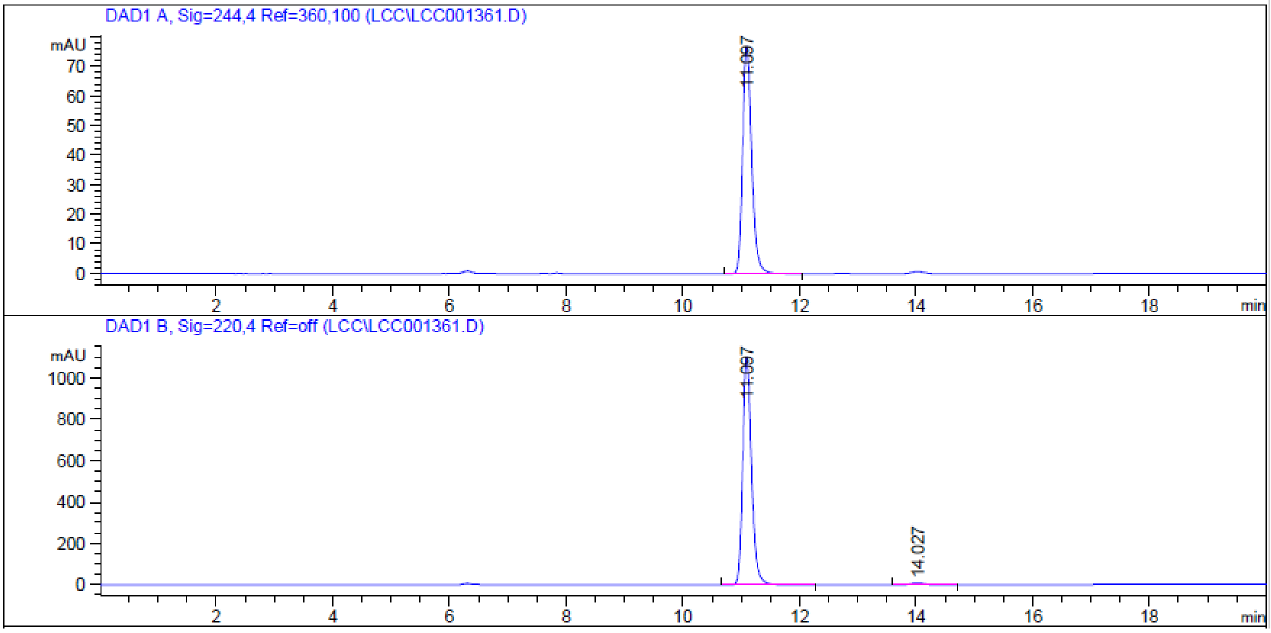

Signal 1: DAD1 A, Sig=244,4 Ref=360,100

Peak #	RetTime [min]	Type	Width [min]	Area [mAU*s]	Height [mAU]	Area %
1	11.097	BB	0.1661	832.12073	76.97348	100.0000
Totals :				832.12073	76.97348	

Signal 2: DAD1 B, Sig=220,4 Ref=off

Peak #	RetTime [min]	Type	Width [min]	Area [mAU*s]	Height [mAU]	Area %
1	11.097	BB	0.1602	1.15354e4	1101.30798	98.7303
2	14.027	BB	0.2488	148.35310	9.26525	1.2697
Totals :				1.16838e4	1110.57323	

Chiral HPLC trace for (*R*)-2-(2-chloro-5-fluorophenyl)pyrrolidine hydrochloride (provided by LCC):

Column : (R,R)WHELK-O1 0.46*5cm;3.5um
Mobile phase B : Hexane (0.1%DEA)
Mobile phase D : IPA
Temperature : 25°C

Method

<<Pump>>
Total Flow : 1.0000 mL/min
B Conc. : 95.0 %
C Conc. : 0.0 %
D Conc. : 5.0 %

<Chromatogram>

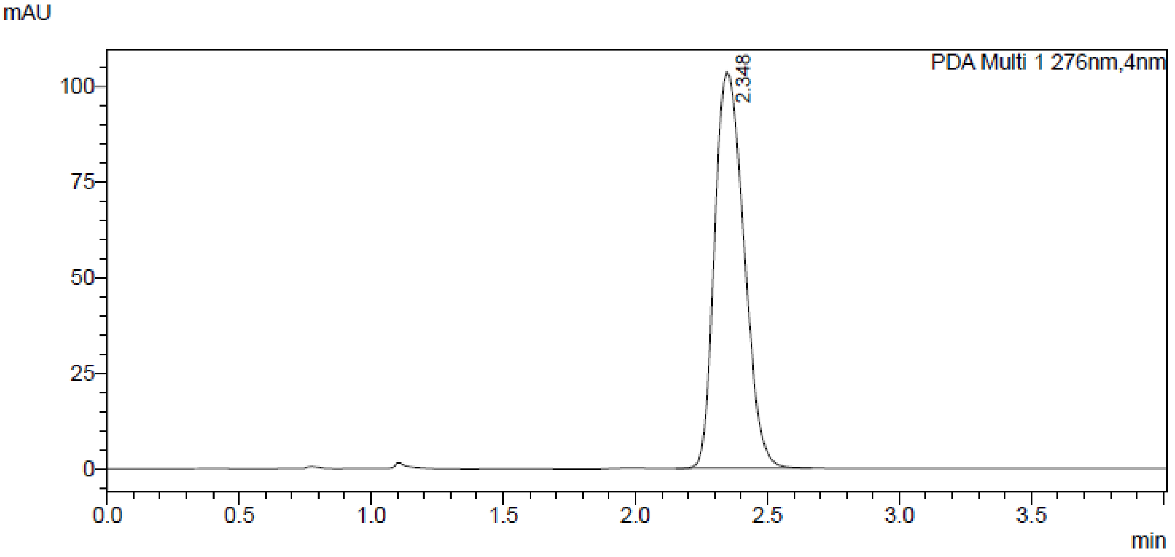

<Peak Table>

PDA Ch1 276nm

Peak#	Ret. Time	Area	Area%	Height	Conc.	Unit
1	2.348	803585	100.000	103887	0.000	
Total		803585	100.000	103887		

4 Supporting Figures

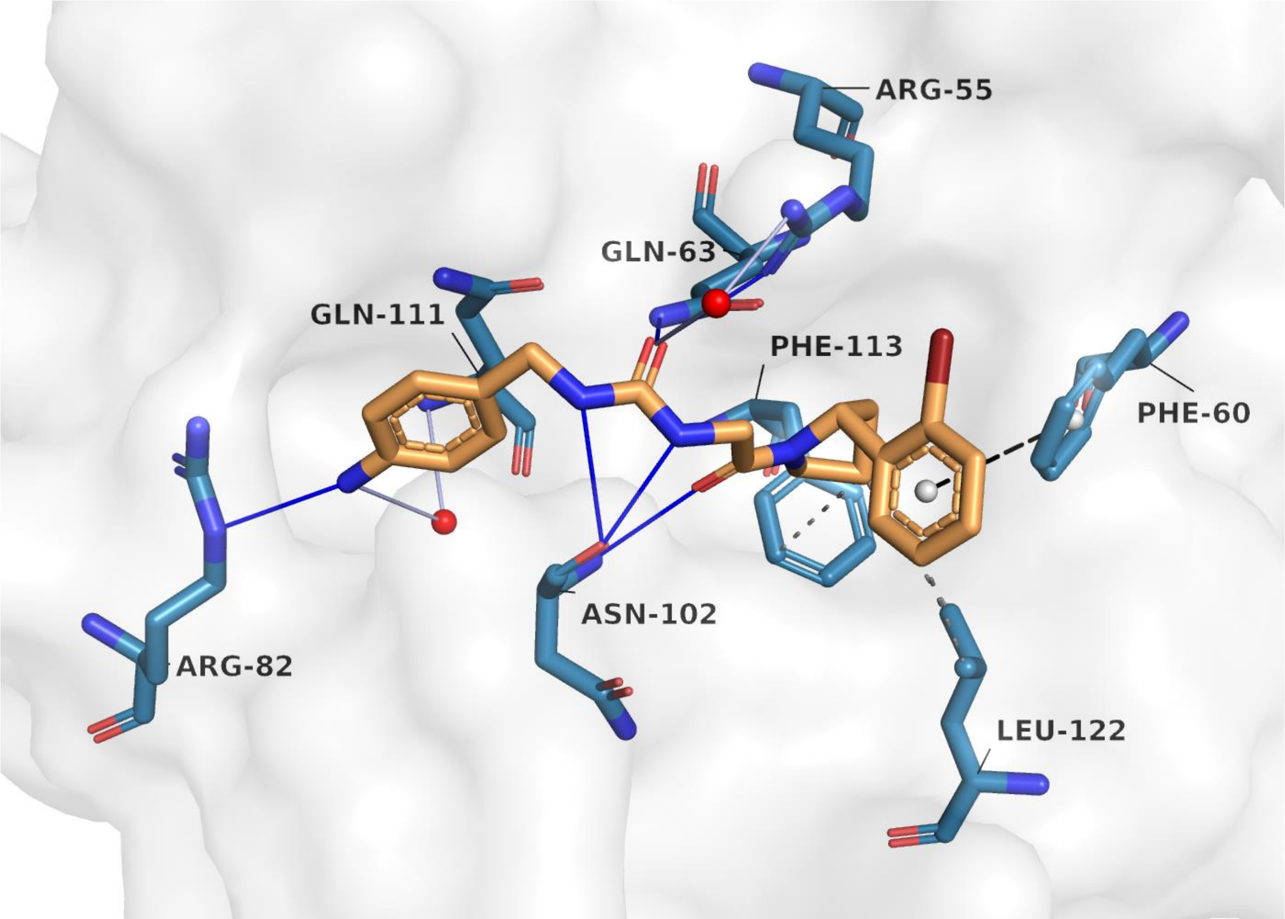

Figure S1. Compound **4a** binding to CypD with key interactions highlighted. Colour key: Protein – Light Grey surface, S1' pocket – Blue surface, S2 pocket – Red surface. Amino acids: Cyan – Carbon, Blue – Nitrogen, Red – Oxygen. Ligand: Orange – Carbon, Blue – Nitrogen, Red – Oxygen, Brown – Bromine. Interactions between Ligand and Protein: Grey Dashed Lines – hydrophobic interactions; Blue Lines – Hydrogen Bonding; Light-blue Lines – water bridges; Black Dashed Lines – T-shaped π-π interactions. X-ray crystal structure (PDB: 4J5D) was rendered in PyMOL Molecular Graphics System, Version 1.7.4.4, Schrödinger, LLC. PLIP was used for the interaction analyses.[3]

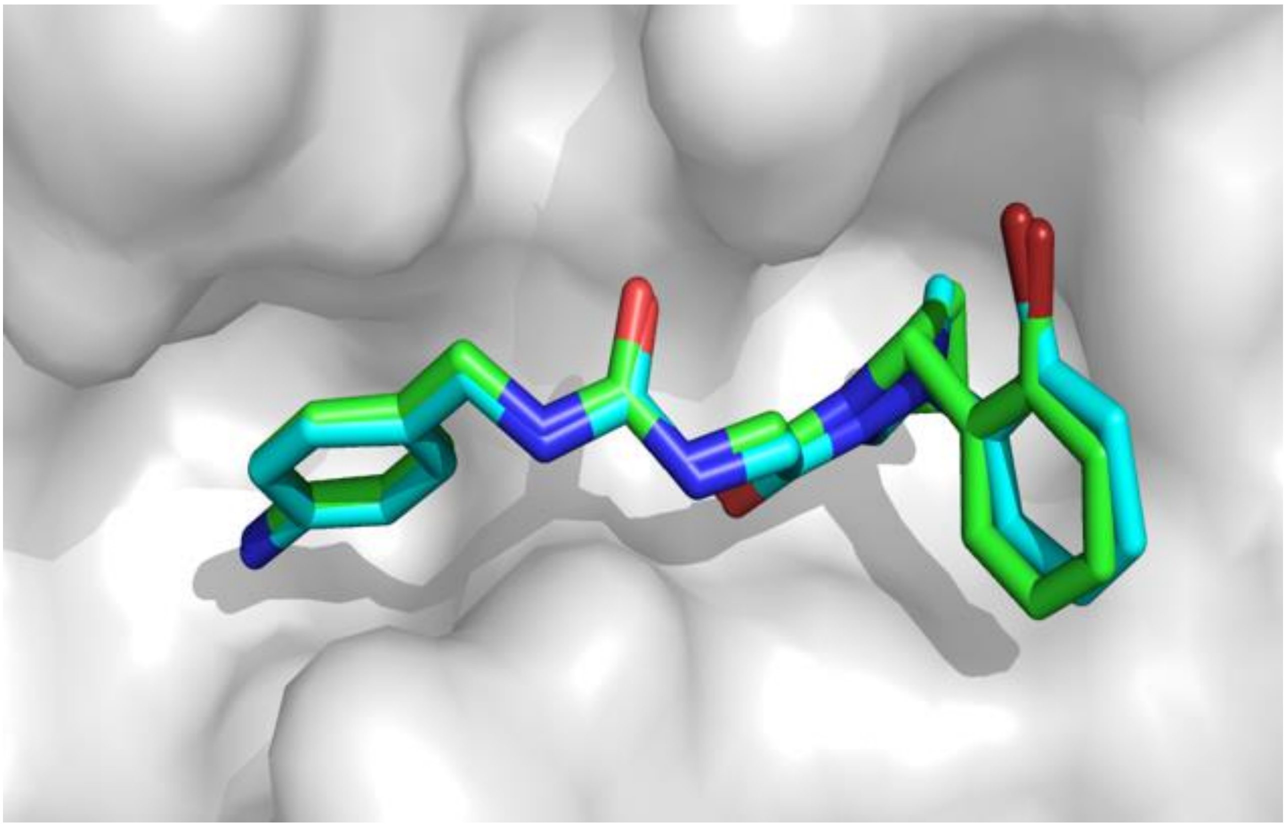

Figure S2: Superposition of CypD X-ray crystal structures with compounds **12a** and **4a** showing an identical ligand binding mode. X-ray crystal structure (PBD: 4J5D) was rendered in PyMOL Molecular Graphics System, Version 1.7.4.4, Schrödinger, LLC.

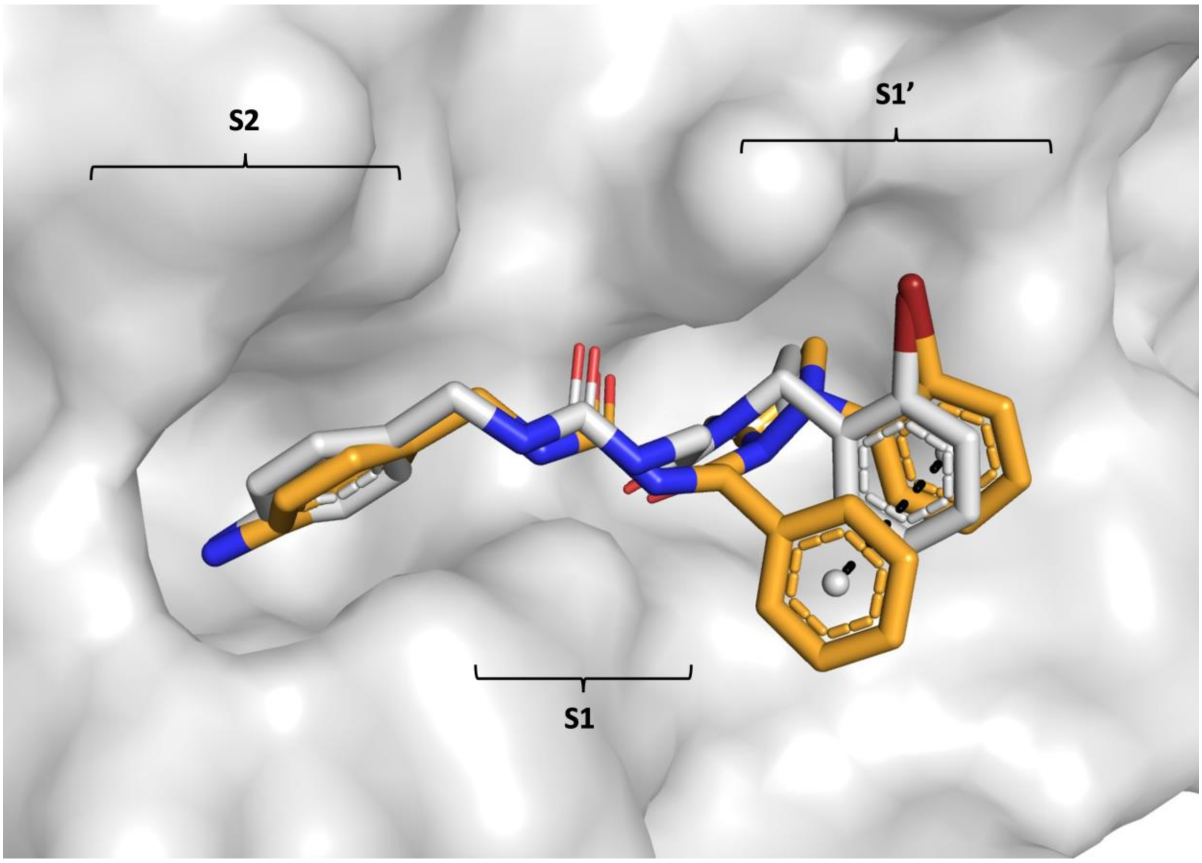

Figure S3: The structure of compound **13** docked into CypD (PDB 4J5D) by the molecular docking package GOLD. The PyMOL image has the following colour key: Protein Surface: Light grey. Docked Ligand: Orange – carbon, Blue – Nitrogen, Red – oxygen, Dark red – bromine. Co-crystallised ligand: Grey – carbon, Blue – Nitrogen, Red – oxygen, Dark red – bromine. Black lines indicate π-π interactions. Docked structure was rendered in PyMOL Molecular Graphics System, Version 1.7.4.4, Schrödinger, LLC.

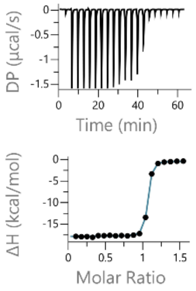

12a

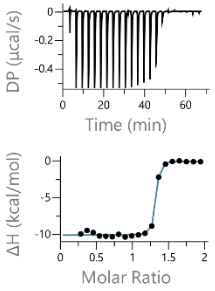

12a

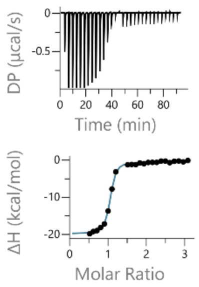

12c

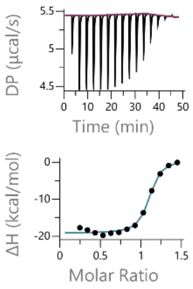

12c

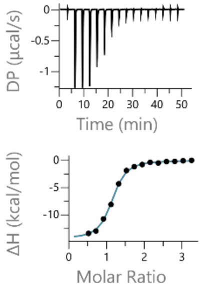

12d

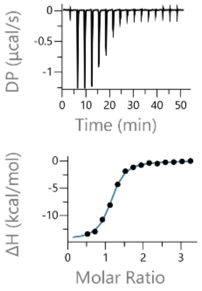

12d

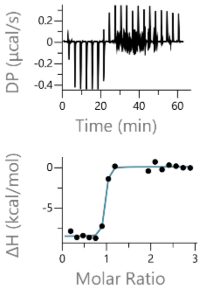

13

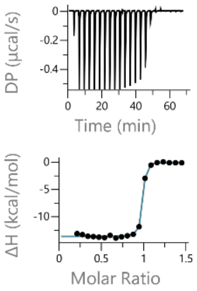

18a

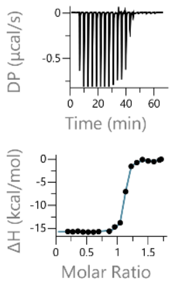

18a

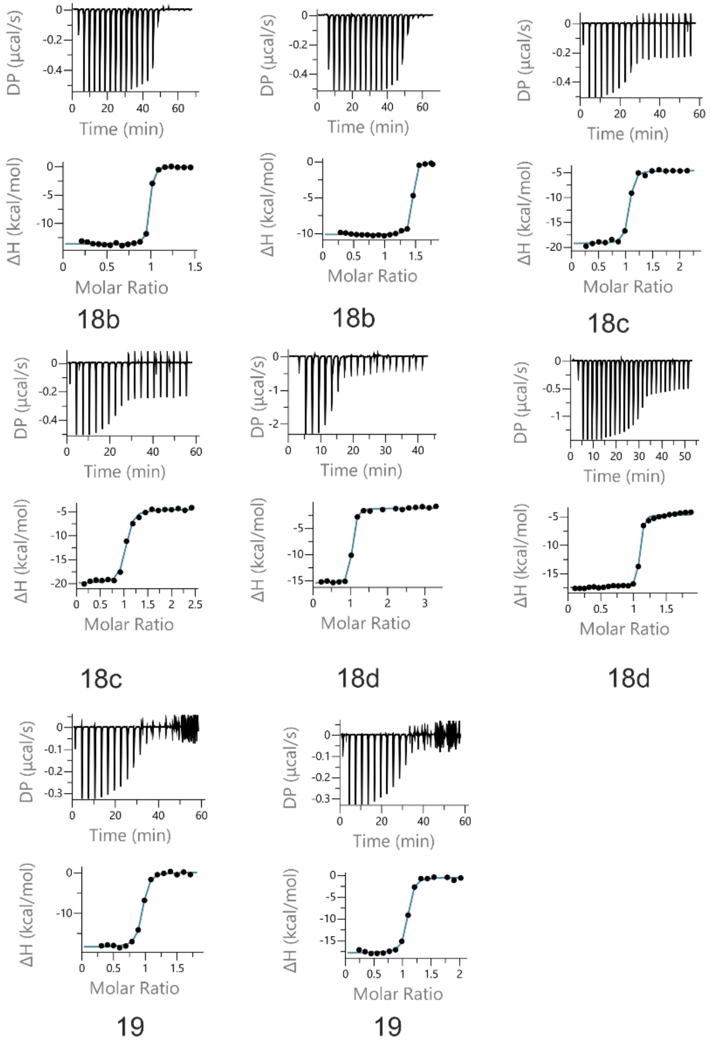

Figure S4. ITC profiles for the binding of selected compounds to CypD (duplicates are shown apart from compound **13**). (a) Compound **12a**, (b) Compound **12c**, (c) Compound **12d**, (d) Compound **13** (e) Compound **18a**, (f) Compound **18b**, (g) Compound **18c**, (h) Compound **18d**, (i) Compound **19**. The top panel shows the raw calorimetric data obtained upon titration of CypD with each ligand; the bottom panel shows the plots of the integrated heat signal as a function of molar ratio of ligand to protein. The data fit to a one-site binding to yield the Kd, ΔH, and ΔS parameters (Figure S5). All

data were corrected for heat of dilution using the fitted offset option in the PEAQ ITC software. For compound **13**, precipitation of the ligand is the most likely cause of the high noise at higher titrant values; nevertheless, the enthalpy changes are well-defined.

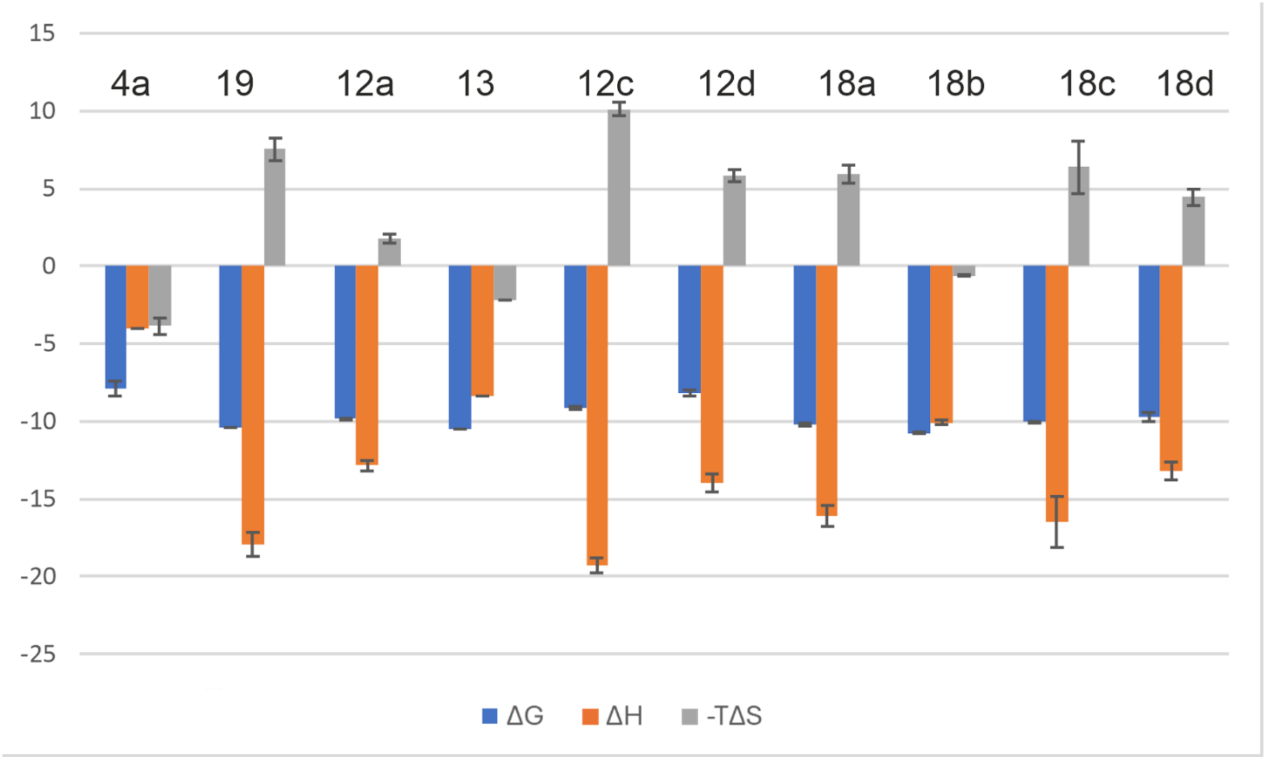

Figure S5. Thermodynamic signatures for the binding of some of compounds listed in Table 1.

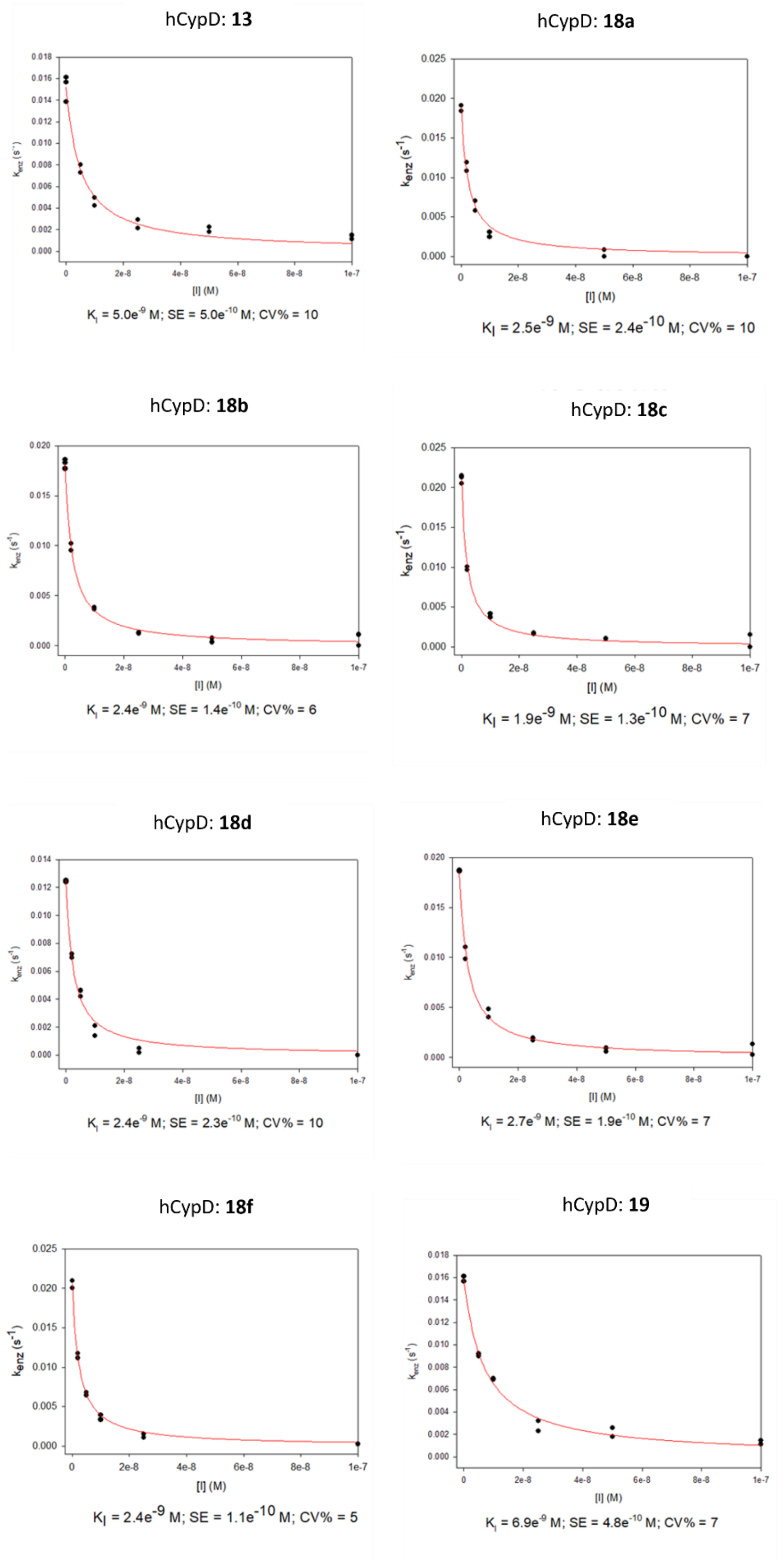

Figure S6. Cyclophilin D (CypD) peptidyl-prolyl isomerase (PPIase) assay. Plot of enzyme rate versus inhibitor cocnetration.

5. Additional NMR Studies

At 213 K in deuterated dichloromethane (DCM), ^{1}H NMR experiments showed that pyrrolidine **1a** was present as a mix of *cis* and *trans* geometric isomers in a ratio of 1.1:1 (Figure S6). Nuclear Overhauser effect (NOE) NMR was utilised to distinguish between *cis* and *trans* isomers since only the acetyl group of the *cis* geometric isomer would be expected to display NOE to H_α on the Pyr ring, and the ortho proton of the aryl ring, as predicted by DFT geometry optimisation (Figure S7). Irradiation of the proposed upfield acetyl protons at 1.8 ppm showed NOE to the more up-field H_α peak at 5.2 ppm and the *ortho* proton on the aryl ring at 7.1 ppm, consistent with the *cis* isomer (Figure S8). Irradiation of the downfield acetyl protons at 2.20 ppm displayed no NOE to any protons in the molecule, consistent with the *trans* geometric isomer (Figure S9). This enabled the *cis*:*trans* ratio to be determined, through integration of the acetyl resonances at 1.8 ppm (*cis* isomer) and 2.2 ppm (*trans* isomer), to be as 1:1.1. This NMR data are in agreement with DFT conformational studies that predicted a slight bias towards the *trans* geometric isomer (Table S1). The results of these NOE experiments are further corroborated by DFT simulated NMR spectra for the *cis* and *trans* isomers.

Only one geometrical isomer was observed in the NMR spectra at 298K for1b with the splitting of peaks in the spectrum unresolved and broad (data not shown). Upon cooling to 213 K, splitting became resolved. The ^{1}H NMR spectrum of Pz compound **1b**, in DCM, is a simpler spectrum than for compound **1a**, with only one set of peaks for each proton group in the molecule (Figure S10). Irradiation of the acetyl peak at 2.00 ppm showed NOE of the *ortho* proton of the aryl ring at 7.08 ppm, consistent with the *cis* geometric isomer (Figure S11). These data, therefore, provide the experimental evidence that the Pz unit is pre-organised in a *cis* conformation compared with the Pyr equivalent. (Table S2)

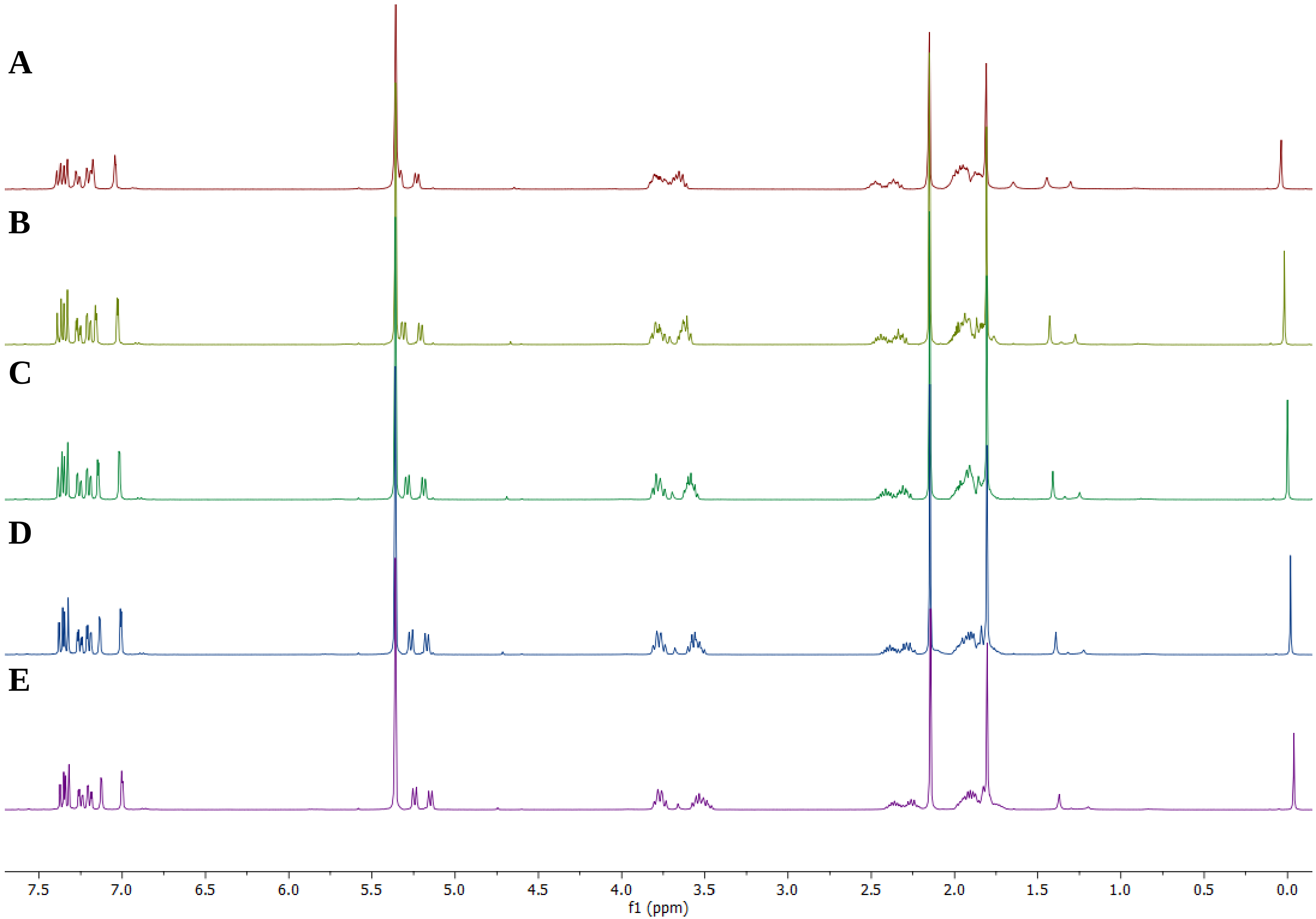

Figure S7: Variable Temperature ^{1}H NMR spectra of **1a** in deuterated DCM at A – 298K, B – 273K, C – 253K, D – 233K, E – 213K.

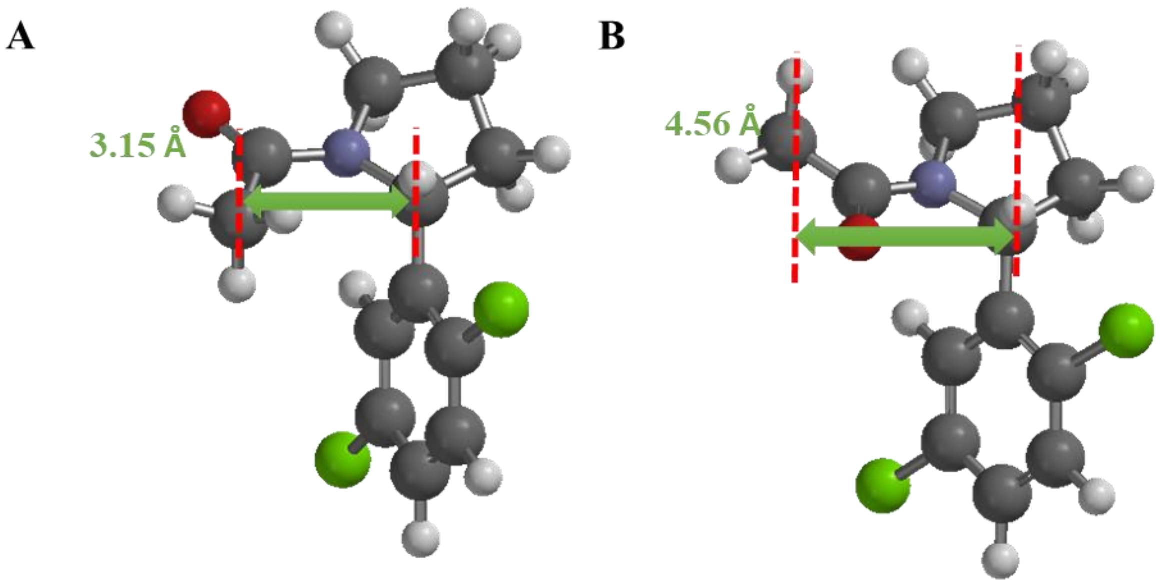

Figure S8. DFT prediction *cis* (**A**) and *trans* (**B**) geometric isomers of compound **1a** with the averaged measured distances between each acetyl proton and the H_α of the Pro ring.

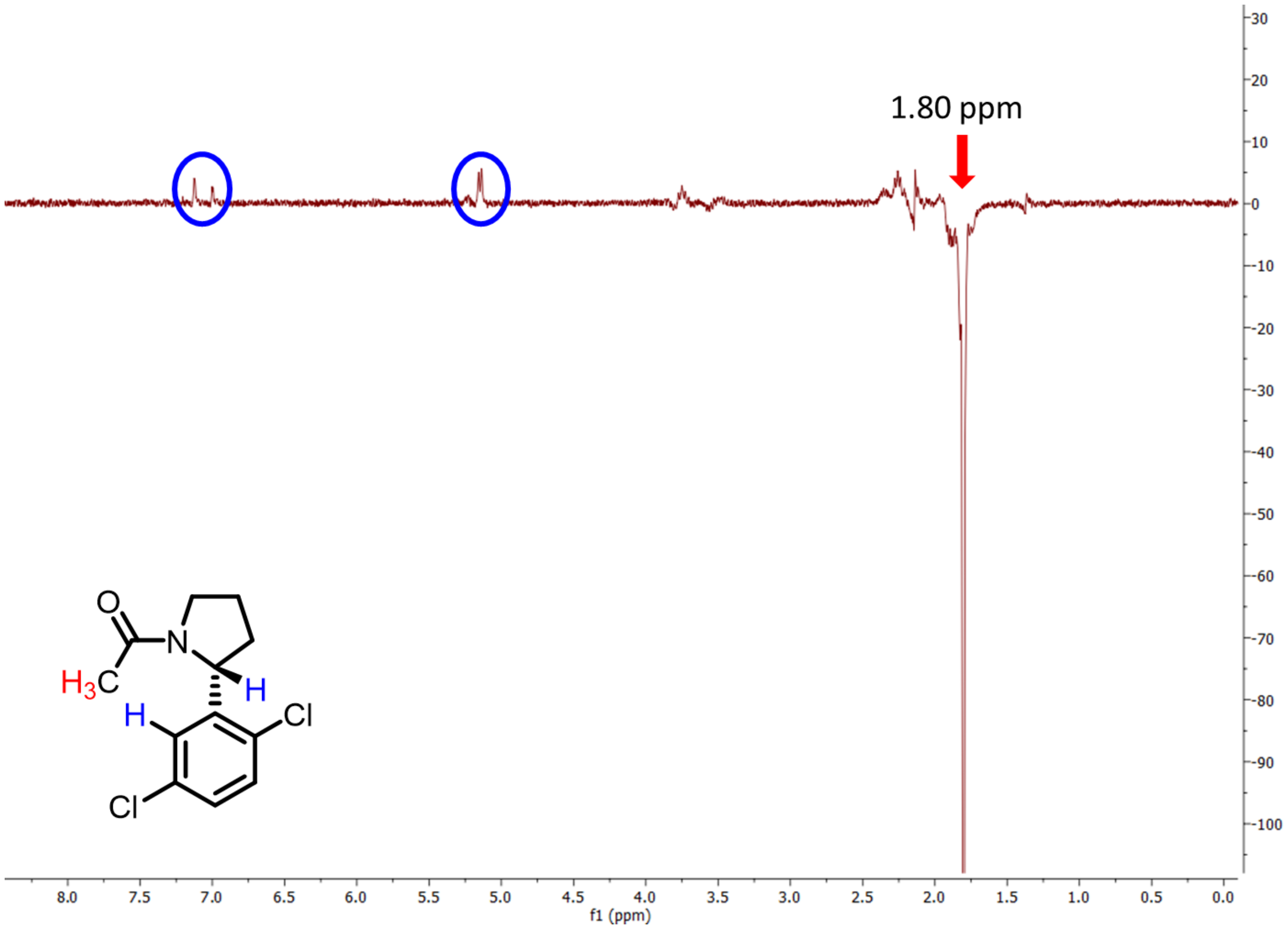

Figure S9: ^{1}H NMR spectrum of compound **1a** in DCM at 213 K, irradiated at the 1.80 ppm acetyl group show NOE to the H_α at 5.1 ppm and the *ortho* proton of the phenyl ring at 7.0 and 7.1ppm.

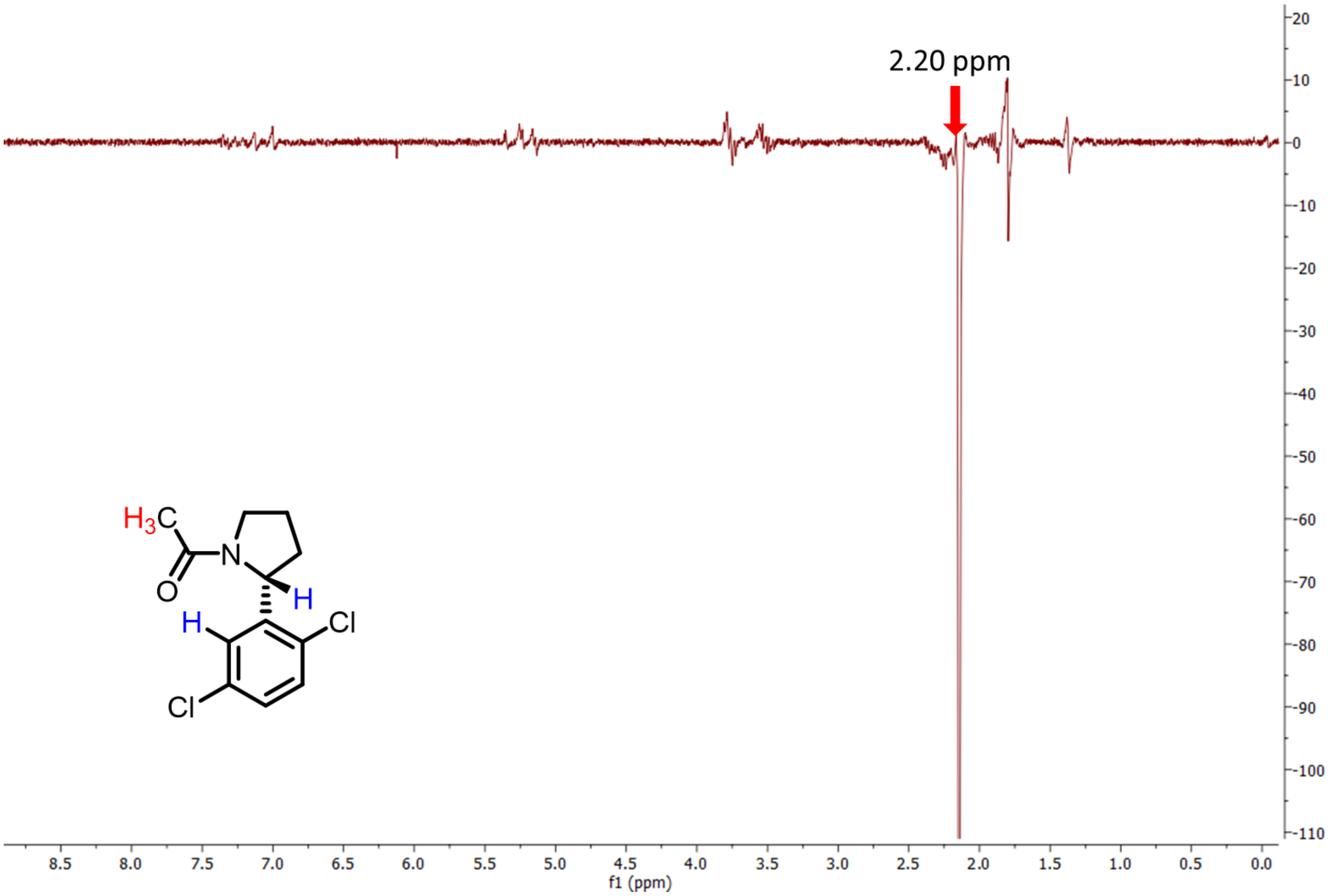

Figure S10: ^{1}H NMR of compound **1a** in deuterated DCM at 213 K, irradiated at the 2.20 ppm acetyl. No NOEs to other protons were observed. Baseline noise is seen due to incidental irradiation of the acetyl peak at 1.80 ppm.

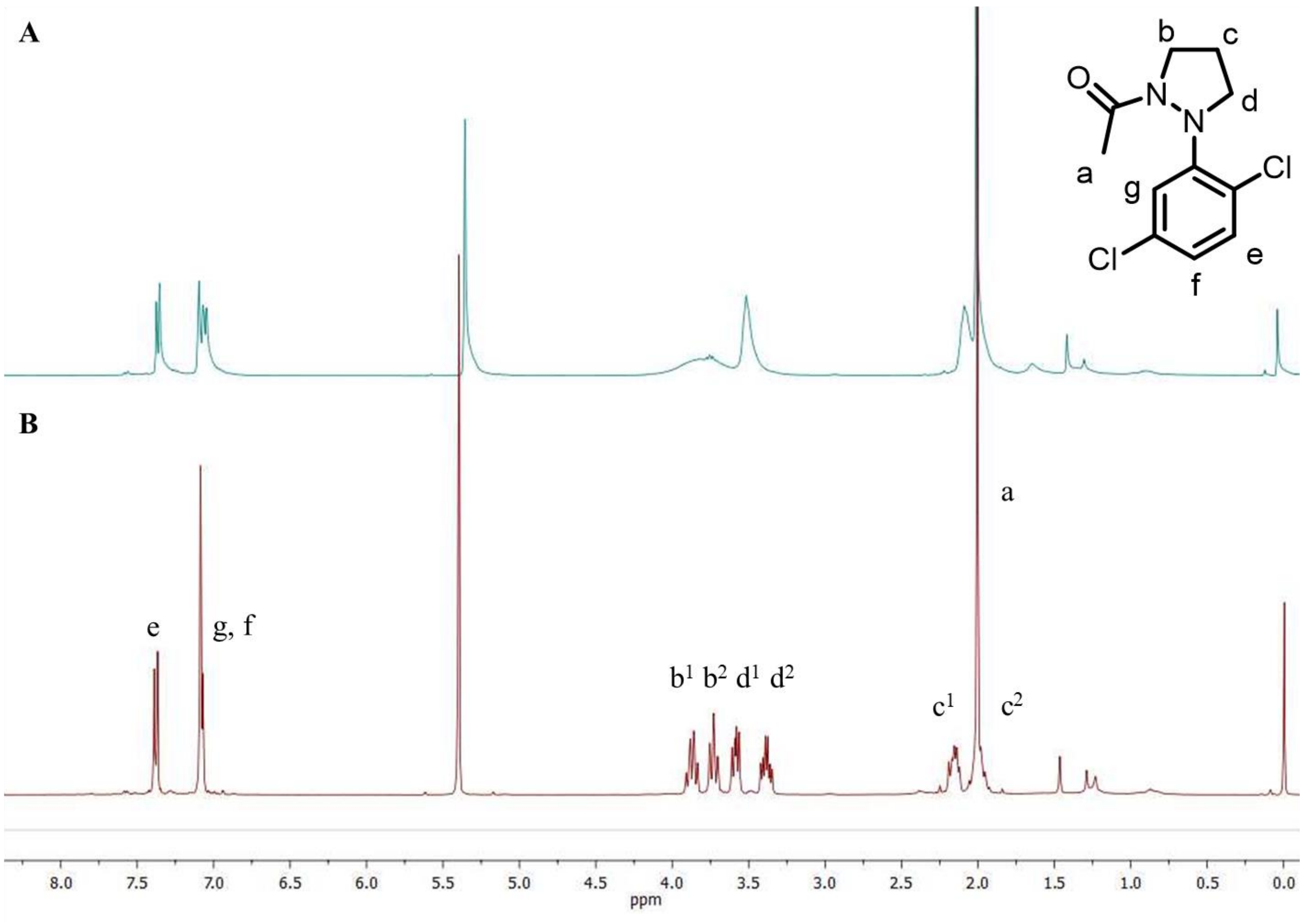

Figure S11. ^{1}H NMR spectrum of compound **1b** in deuterated DCM (**A**) at 298 K and (**B**) at 213 K. Assignments are based on NOE experiments, *J* coupling correlation and simulated ^{1}H NMR spectra.

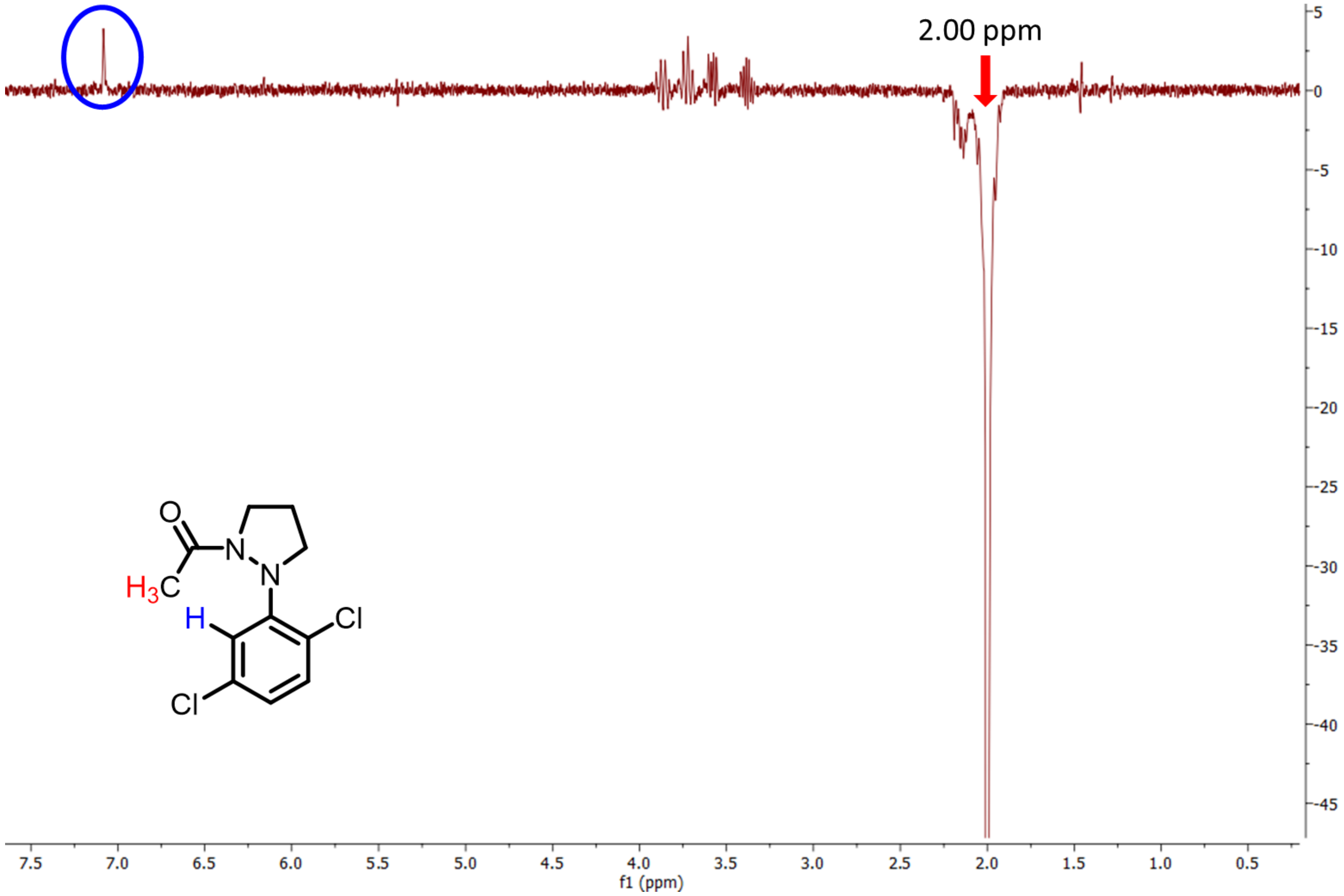

Figure S12: ^{1}H spectrum of compound **1b** in deuterated DCM at 213 K, irradiated at the 2.00 ppm acetyl show NOE to the *ortho* proton of the phenyl ring at 7.1 ppm.

6. Supporting Schemes

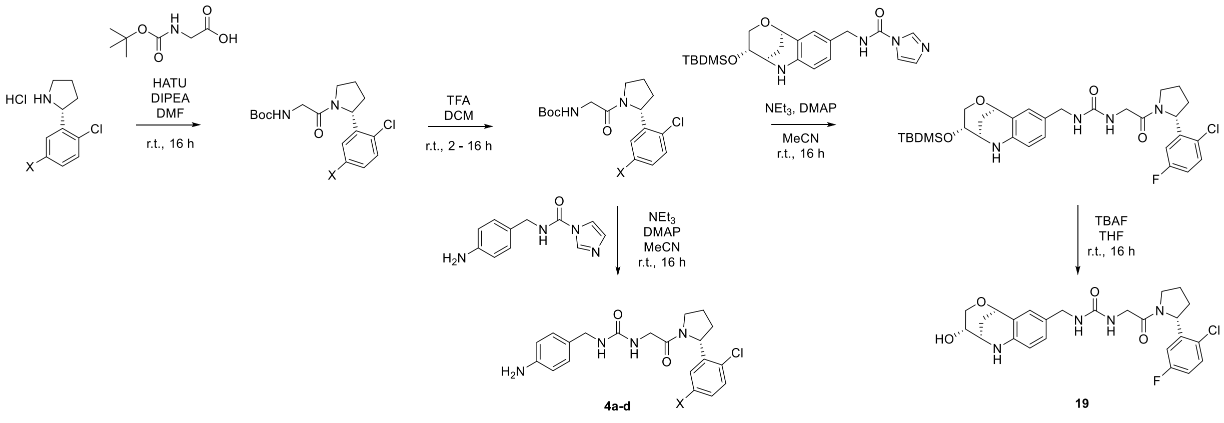

Scheme S1. Synthesis of Pyr analogues 4a-d and **19** which was prepared using a similar synthetic route as used for the aniline series with the synthesis of the starting materials outsourced.

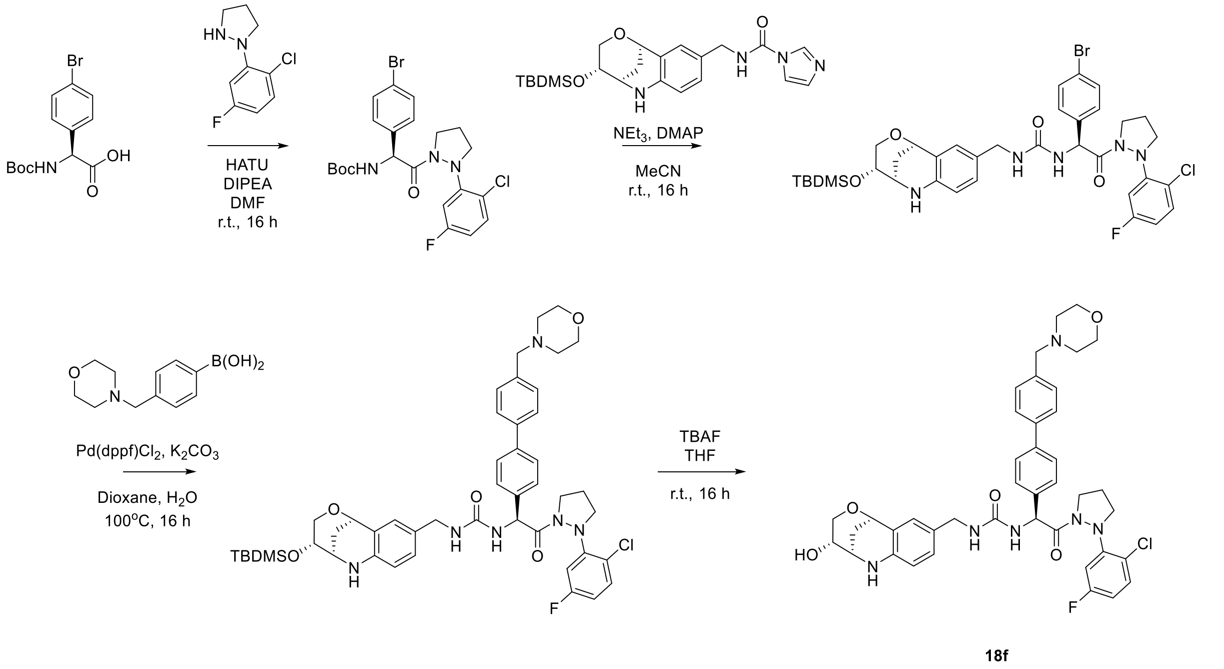

Scheme S2. Synthesis of biaryl-methylmorpholine analogue **18f** which was prepared using a similar synthetic route as used for the Pz series.

7. Supporting Tables

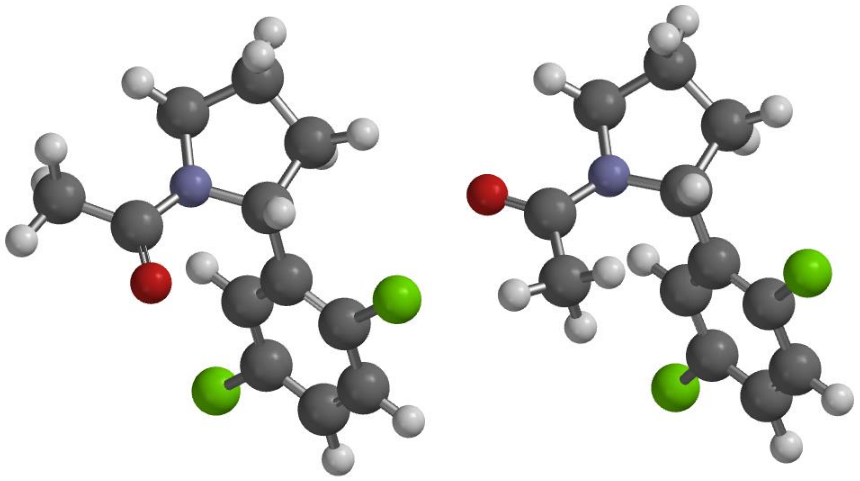

Table S1: Calculated energies of conformers of the model Pyr **1a**. Lowest energy *cis* and *trans* conformations (conformation **1** and conformation **2** respectively) are shown in bold and displayed in ball and stick representations (above).

Rank	G (au)	Grel (Kcal/mol)	Amide Geometry
**1**	**-1515.11819**	**0**	***Trans***
**2**	**-1515.11811**	**0.05**	***Cis***
3	-1515.11584	1.47	*Trans*
4	-1515.1157	1.56	*Cis*

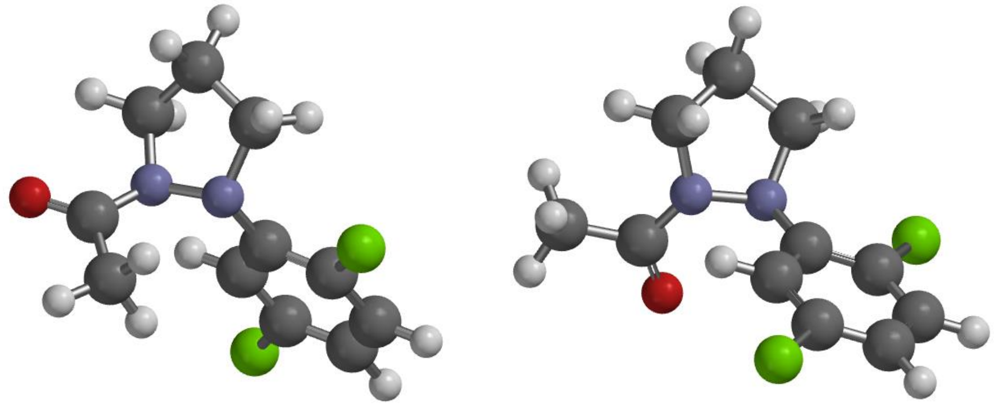

Table S2: Calculated energies of conformers of the model Pz **1b**. Lowest energy *cis* and *trans* conformations (conformation **1** and conformation **3** respectively) are shown in bold and displayed in ball and stick representations (above).

Rank	G (au)	Grel (Kcal/mol)	Amide Geometry
**1**	**-1531.12158**	**0.00**	***Cis***
2	-1531.12151	0.04	*Cis*
**3**	**-1531.11698**	**2.89**	***Trans***
4	-1531.11697	2.89	*Trans*
5	-1531.11394	4.79	*Cis*
6	-1531.11376	4.91	*Cis*
7	-1531.11153	6.31	*Trans*
8	-1531.11142	6.38	*Trans*

Table S3: CytochromeP450 isoform- and regio-selectivity was predicted for CypD inhibitors reported in this work through the use of predictive models implemented in the P450 module of the cheminformatics package Stardrop. Orange labels indicate labile positions, yellow – moderate, grey – low. Numbers in labels indicate percentage contribution of metabolite formation at a given position to overall predicted metabolism.

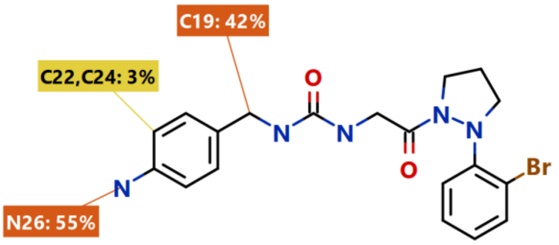

12a

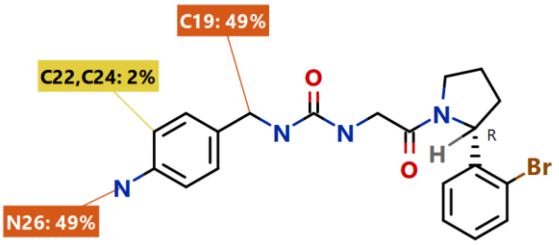

4a

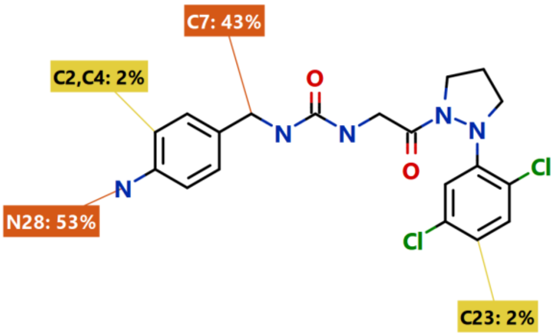

12b

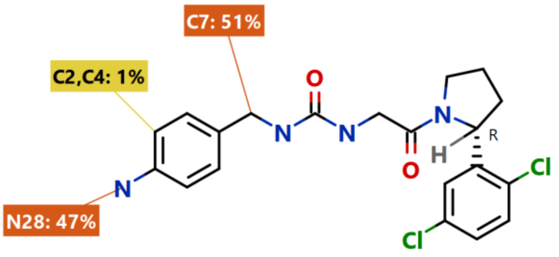

4b

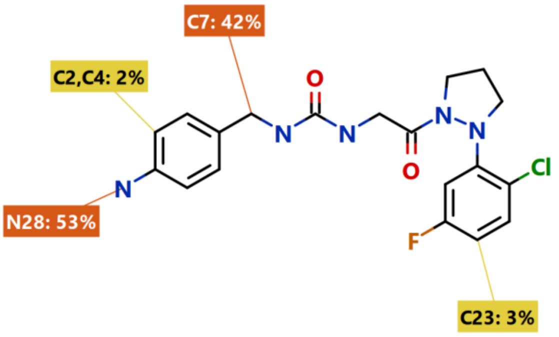

12c

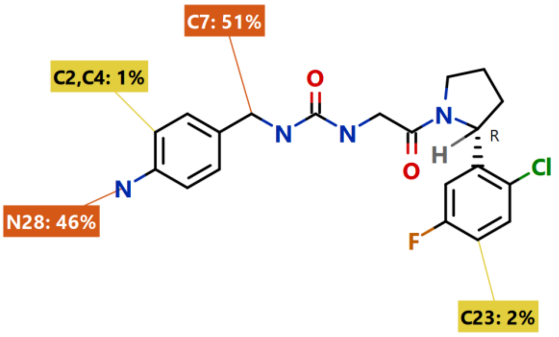

4c

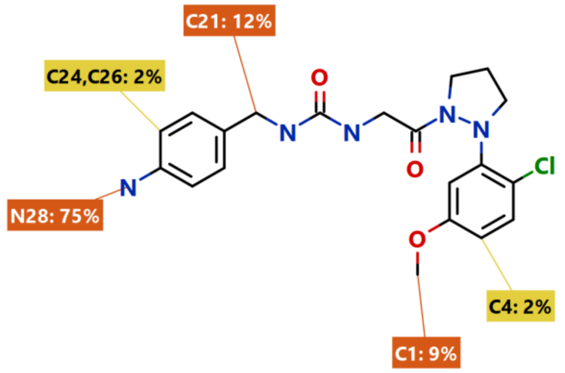

12d

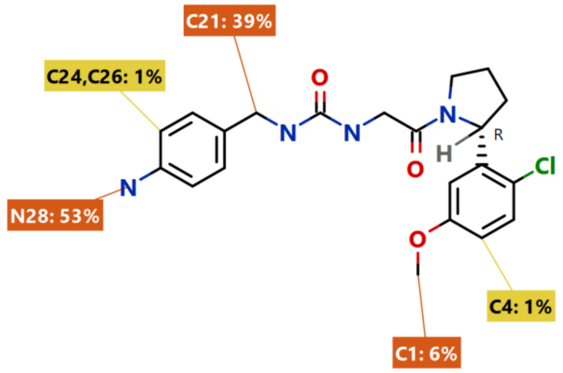

4d

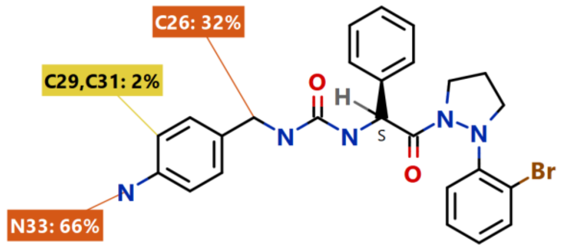

13

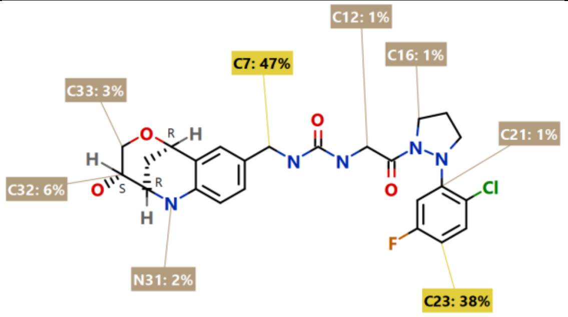

18a

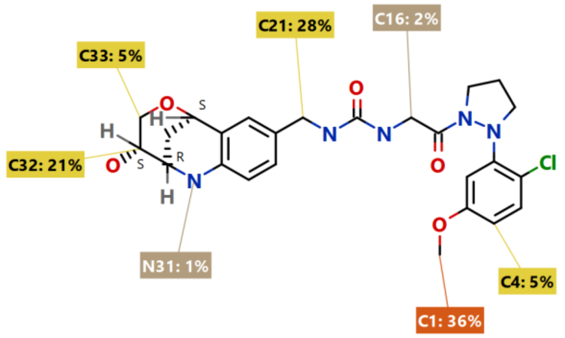

18b

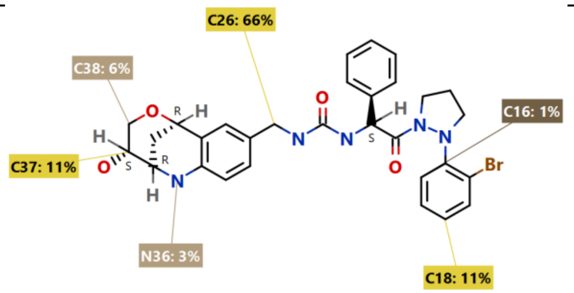

18c

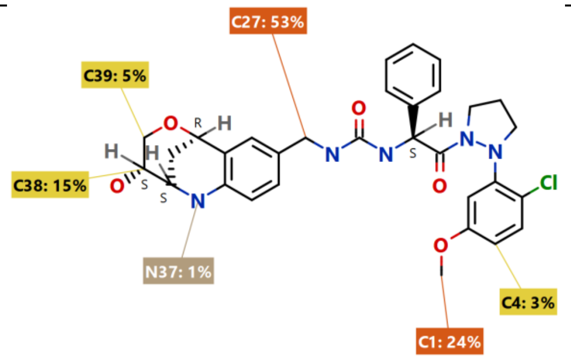

18d

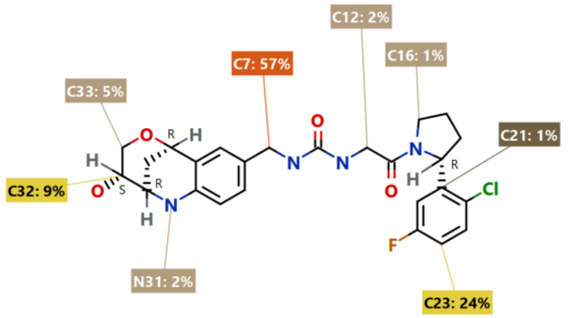

19

Table S4: X-ray data collection and refinement statistics

	**Compound 12a**	**Compound 18d**
**Data Collection**		
Wavelength (Å)	0.97857	1.54179
Resolution Range (Å)	51.39-1.45	41.00-2.25
Space Group	p43212	p43212
Unit Cell		
a, b, c (Å)	57.45 57.45 114.91	57.13 57.13 117.77
α, β, γ (°)	90 90 90	90 90 90
Unique reflections	34902(2468)	9525 (857)
Multiplicity	13.3(13.1)	6.5 (5.4)
Completeness	100(99.5)	98.0 (99.0)
Mean I/σ	15.9 (4.8)	13.9 (3.7)
*R*merge	0.063 (0.409)	0.105 (0.456)
CC1/2	0.999 (0.903)	0.997 (0.837)
**Refinement**		
Rwork	16.82(24.71)	16.03 (17.56)
Rfree	19.54(27.31)	18.48 (23.51)
RMSD(BondsÅ)	0.0055	0.004
RMS(Angles°)	0.851	0.794
Ramachamdran		
Favoured (%)	97.53	96.3
Outliers (%)	0	0

R-free was calculated using ~5% free reflections isolated from refinement, overall values are shown with highest resolution shell shown in parenthesis.

Table S5: Summary of non-covalent interactions of compound **12a** with the binding site of CypD as determined by PLIP[3] using default settings with the exception of hydrophobic interaction cut-off distance which was set at 4.3 Å.

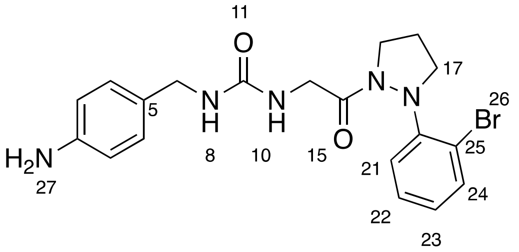

12a

Entry	Ligand Atoms	Residue	Distance (Å)	
Hydrophobic Interactions				
1	21	Phe113	3.73	
2	17	Leu122	3.73	
π-π Stacking				
14	20–25	Phe60	5.20	
Hydrogen Bonds			Distance H-A[a]	Distance D-A[b]
3	11	Arg55	2.18	3.01
4	11	Gln63	1.93	2.91
5	15	Asn102	2.42	3.08
6	10	Asn102	2.09	3.01
7	8	Asn102	2.28	2.94
8	15	Thr107	2.57	3.02
Water Bridges			Distance Lig-W[c]	Distance Prot-W[d]
9	11	Arg55	3.35	2.84
10	11	Arg55	3.78	3.59
11	8	Arg55	4.04	3.59
12	15	Gln111	3.69	3.34
13	15	Gln111	2.79	4.10

[a] Distance between the hydrogen of a hydrogen bond donor and the acceptor atom. [b] Distance between the heteroatom of a hydrogen bond donor and the acceptor atom. [c] Distance between the ligand atom and water in a water bridge interaction. [d] Distance between the protein atom and the water involved in a water bridge interaction

Table S6: Summary of non-covalent interactions of compound **18d** with the binding site of CypD as determined by PLIP[3] using default settings with the exception of hydrophobic interaction cut-off distance which was set at 4.3 Å.

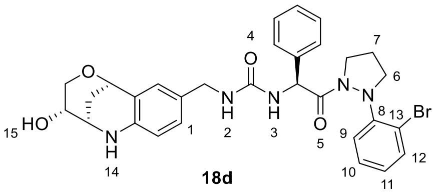

18d

Entry	Ligand Atoms	Residue	Distance (Å)	
Hydrophobic Interactions				
1	1	Ala101		4.05
2	1	Gln111		4.23
3	7	Phe113		3.28
4	7	Leu122		4.20
5	10	Leu122		3.74
π-stacking				
14	8–13	Phe60		4.98
Hydrogen Bonds			Distance H-A[a]	Distance D-A[b]
3	4	Arg55	2.13	2.88
4	11	Gln63	1.88	2.85
	15	Ser81	2.44	3.33
5	15	Arg82	3.29	4.00
6	5	Asn102	2.23	3.14
7	3	Asn102	2.13	2.95
8	2	Asn102	2.13	2.95
	14	Thr107	2.35	2.87
Water Bridges			Distance Lig-W[c]	Distance Prot-W[d]
9	11	Arg55	3.19	3.19
10	15	Ser81	3.38	3.16
	14	Gly109	3.93	2.84
12	14	Gln111	3.03	3.97
π-stacking				
14	8–13	Phe60		4.98

[a] Distance between the hydrogen of a hydrogen bond donor and the acceptor atom. [b] Distance between the heteroatom of a hydrogen bond donor and the acceptor atom. [c] Distance between the ligand atom and water in a water bridge interaction. [d] Distance between the protein atom and the water involved in a water bridge interaction

8. Supporting Methods

Computational Details

Structures were built and optimised in Spartan20 unless specified otherwise. Conformers were generated by a molecular mechanics conformer distribution search as implemented in Spartan20 followed by geometry optimisation at the ωB97X-D/6-31G* level in solvent DCM implemented using the CPCM method. Stated energies are free energies obtained from single point frequency calculations on the optimised structures. Simulated NMR spectra were calculated at the same level of theory.

Natural bond orbital (NBO) analysis of the simplified azaproline was performed by use of the NBO module as implemented in Gaussian16 following optimisation of the lowest energy conformer at the same level of theory. Orbital cube files were generated in Multiwfn[3] and rendered in VMD.[4]

Molecular docking studies were carried out using the molecular docking package GOLD and the interface Hermes as part of the GOLD suite.[5] CypD inhibitors were docked into the binding site of CypD using an obtained crystal structure of CypD co-crystallised with **4** (PDB: 4J5D). Genetic algorithm runs were carried out using default settings and scored using the ChemPLP scoring function. The docking protocol was validated by re-docking of the extracted ligand (average RMSD < 1.5 Å).

9. References

1. Shore, E. R., et al., *J. Med. Chem.* **2016,** *59* (6), 2596-2611.
2. Jorand-Lebrun, C., et al. Preparation of urea and amide compounds for the inhibition of cyclophilins. WO2017173048, 2017.
3. Adasme, M. F., et al., *Nuc. Acids Res.* **2021,** *49* (W1), W530-W534.
4. Humphrey, W., Dalke, A. & Schulten, K. VMD: visual molecular dynamics. *J. Mol. Graph.* **1996**, 14, 33-38 .
5. https://www.ccdc.cam.ac.uk/solutions/software/hermes/
